# Supplementary material for: Highly efficient color-tunable organic co-crystals unveiling polymorphism, isomerism, delayed fluorescence for optical waveguides and cell-imaging
Source: Nat Commun. 2023 Oct 20;14:6648. doi: 10.1038/s41467-023-42017-8 (PMC10589249; doi:10.1038/s41467-023-42017-8)
Supplement: Supplementary file 1 — Supplementary Information [file 41467_2023_42017_MOESM1_ESM.pdf]

# Supplementary Information

## Highly Efficient Color-Tunable Organic Co-crystals Unveiling Polymorphism, Isomerism, Delayed Fluorescence for Optical Waveguides and Cell-imaging

Debasish Barman[1], Mari Annadhasan[2], Anil Parsram Bidkar[3], Pachaiyappan Rajamalli[4], Debika Barman,[1] Siddhartha Sankar Ghosh[5,6*], Rajadurai Chandrasekar[2*] & Parameswar Krishnan Iyer[1,6*]

[1]Department of Chemistry, Indian Institute of Technology Guwahati, Guwahati-781039, India.

[2]School of Chemistry, and Centre for Nanotechnology University of Hyderabad, Gachibowli, Prof. C. R. Rao Road, Hyderabad-500046, India.

[3]Department of Radiology and Biomedical Imaging, University of California, San Francisco, San Francisco, California 94143, United States.

[4]Materials Research Centre, Indian Institute of Science Bangalore-560012, India.

[5]Department of Bioscience and Bioengineering IIT Guwahati, Guwahati, Assam, India.

[6]Centre for Nanotechnology, Indian Institute of Technology Guwahati, Guwahati-781039, India.

*e-mail: pki@iitg.ac.in

*e-mail: r.chandrasekar@uohyd.ac.in

*e-mail: sghosh@iitg.ac.in

## Supplementary Table of Contents


| Title | Page No |
|---|---|
| **Supplementary Fig. 1.** Fluorescence Microscope images of the prepared co-crystals. | **S5** |
| **Supplementary Fig. 2.** FTIR spectra of TAH crystals (green line), OFN (orange line) and TAHOFN (red line). | **S5** |
| **Supplementary Fig. 3.** FTIR spectra of TAH crystals (green line), TFPN (yellow line) and TAHTFPN_G (black line). | **S6** |
| **Supplementary Fig. 4.** FTIR spectra of TAH crystals (green line), TFPN (yellow line) and TAHTFPN_O (black line). | **S6** |

**Supplementary Fig. 5.** FTIR spectra of TAH crystals (green line), TCNB (yellow line) and TAHTCNB (black line). S7

**Supplementary Fig. 6.** Raman spectra of TAH (deep green line) OFN (orange line) and TAHOFN (red line). S7

**Supplementary Fig. 7.** Raman spectra of TAH (deep green line) TFPN (greenish yellow line) and TAHTFPN_G (black line). S8

**Supplementary Fig. 8.** Raman spectra of TAH (deep green line) TFPN (greenish yellow line) and TAHTFPN_O (black line). S8

**Supplementary Fig. 9.** Raman spectra of TAH (deep green line) TCNB (greenish yellow line) and TAHTCNB (black line). S9

**Supplementary Fig. 10.** Experimental PXRD pattern for TAH (green line), OFN (orange line) and TAHOFN with calculated PXRD (red lines). S9

**Supplementary Fig. 11.** Experimental PXRD pattern for TAH (green line), TFPN (yellow line) and TAHTFPN_G with calculated PXRD (black lines). S10

**Supplementary Fig. 12.** Experimental PXRD pattern for TAH (green line), TFPN (yellow line) and TAHTFPN_O with calculated PXRD (black lines). S10

**Supplementary Fig. 13.** Experimental PXRD pattern for TAH (green line), TCNB (yellow line) and TAHTCNB with calculated PXRD (black lines). S11

**Supplementary Fig. 14.** Thermal gravimetric analysis (TGA) study for TAH (green line), OFN (yellow line) and TAHOFN (black lines). S11

**Supplementary Fig. 15.** Thermal gravimetric analysis (TGA) study for TAH (green line), TFPN (yellow line) and TAHTFPN (black lines). S12

**Supplementary Fig. 16.** Thermal gravimetric analysis (TGA) study for TAH (green line), TFPN (yellow line) and TAHTFPN_O (black lines). S12

**Supplementary Fig. 17.** Thermal gravimetric analysis (TGA) study for TAH (green line), TCNB (yellow line) and TAHTCNB (black lines). S13

**Supplementary Fig. 18.** Differential scanning calorimetry (DSC) study for TAHOFN, TAHTFPN_G, TAHTFPN_O and TAHTCNB respectively. S13

**Supplementary Fig. 19.** Stacked $^1$H NMR spectra for TAHOFN, TAHTFPN and TAHTCNB co-crystals, recorded in DMSO-*d6* and $D_2O$ mixture at 298K. S14

**Supplementary Table 1-4.** Calculated attachment energies of different crystal facets of TAHOFN, TAHTFPN_G, TAHTFPN_O, TAHTCNB co-assembly. S14-S15

**Supplementary Table 5**. Crystallographic data and structure refinement parameters of TAHOFN, TAHTFPN_G, TAHTFPN_O and TAHTCNB co-crystals. S16

**Supplementary Fig. 20.** Twisted angle between two planes of planar TAH and central phenyl planes in difference interactive orientation to the guest molecules (OFN, TFPN and TCNB) S17

**Supplementary Fig. 21.** Unit-cell packing in the SC-XRD structure for TAHOFN, TAHTFPN_G, TAHTFPN_O, TAHTCNB showing close contact with mixed stack and segregated stack alignment between TAH and OFN, TFPN and TCNB cores. S17

**Supplementary Fig. 22.** Supramolecular bulk assembly of TAHOFN, TAHTFPN_G, TAHTFPN_O and cis-TAHTCNB S18

**Supplementary Table 6.** SCXRD-crystal structural analysis with non-covalent interactions and dihedral angle for all the afforded co-crystals. **S18**

**Supplementary Fig. 23-26.** 2D Finger print for surface to atom intermolecular interactions mapped on Hirshfeld surface in TAHOFN, TAHTFPN_G, TAHTFPN_O and TAHTCNB. **S19-S20**

**Supplementary Fig. 27.** Orientation of dipole moment (μ) and pitch angles between D-A obtained from TD-DFT and SC-XRD structures of TAHOFN, TAHTFPN_G, TAHTFPN_O and TAHTCNB. **S21**

**Supplementary Fig. 28.** Solid state UV-visible spectra of TAHOFN, TAHTFPN_G, TAHTFPN_O and TAHTCNB co-crystals. **S21**

**Supplementary Fig. 29.** Photophysical and morphological characterization of TAH donor. **S22**

**Supplementary Fig. 30.** FETEM images (inset: DLS graph for size distribution) of co-crystal nano aggregates formed in DMSO/$H_2O$ (99%, $f_w$) for TAHOFN, TAHTFPN_G and TAHTCNB **S22**

**Supplementary Fig. 31-33.** Zeta-potential recorded in $H_2O$ for TAHOFN, TAHTFPN_G and TAHTCNB at $10^{-5}$ M concentration. **S23-S24**

**Supplementary Fig. 34-37.** PLQY measurement for TAHOFN, TAHTFPN_G, TAHTFPN_O and TAHTCNB co-crystals. **S24-S26**

**Supplementary Table 7-10**. Time resolved PL Decay of TAHOFN, TAHTFPN_G, TAHTFPN_O and TAHTCNB co-crystal $\lambda_{Ex.}$ 405 nm and $\lambda_{Em.}$ 540, 545, 560 and 590 nm. **S26-S27**

**Supplementary Fig. 38.** a) Temperature dependent delayed emission spectra for TAHTCNB co-crystal in solid state. b) PL at RT and phosphorescence at 77K. c) Steady state PL at RT and 77K for TAHTCNB solid state (powder). **S28**

**Supplementary Table 11-13**. Fitting results for delayed decay at 300, 200 and 100K for TAHTCNB co-crystal. **S28-S29**

**Supplementary Fig. 39-42.** Cyclic voltammetry curves of TAH crystals (green line), OFN, TFPN, TCNB (orange/yellow line) and TAHOFN, TAHTFPN_G, TAHTCNB_O and TAHTCNB (red/black line). **S29-S31**

**Supplementary Table 14.** The energy levels calculated from cyclic voltammetry and DFT. **S32**

**Supplementary Fig. 43-46.** Natural transition orbitals (NTOs) for lowest excited singlet and triplet excited states for TAHOFN, TAHTFPN_G, TAHTFPN_O and TAHTCNB. **S32-S34**

**Supplementary Fig. 37.** Mechanism of weak fluorescence (a-PET & d-PET) for TAHOFN by DFT/TD-DFT calculated HOMO/LUMO energy band diagram for conformer TAH and OFN. **S34**

| | |
|---|---|
| **Supplementary Fig. 48.** Color-tunable fluorescence and CT mechanism for the polymorphic co-crystals of TAHTFPN_G and TAHTFPN_O by DFT/TD-DFT calculated HOMO/LUMO energy band diagram for the conformer TAH and TFPN. | **S35** |
| **Supplementary Fig. 49.** Red fluorescence and strong narrow band CT mechanism for the cis-isomeric co-crystals of TAHTCNB by DFT/TD-DFT calculated HOMO/LUMO energy band diagram for the conformer TAH and TCNB. | **S35** |
| **Supplementary Table 15-22.** Vertical energy transitions with major frontier orbital contribution for TAHOFN, TAHTFPN_G, TAHTFPN_O and TAHTCNB cocrystals at B3LYP/6-31G(d,p) level for singlet and triplet states. | **S36-S40** |
| **Supplementary Table 23.** SOC-matrix element (SOCME) calculated by using ORCA 4.5.0 software with ORCA_SOC module | **S41** |
| **Supplementary Fig. 50.** Electron spin resonance (ESR) spectra for TAH (yellow line), TAHOFN (off green line), TAHTFPN_G (green line) and TAHTCNB (red line). | **S41** |
| **Supplementary Fig. 51.** Chemical structures of OFN, TFPN and TCNB molecule, where x, y, and z are the bond length of carbon-carbon bond at neutral state. | **S42** |
| **Supplementary Fig. 52-54.** Spatially resolved spectra of TAHOFN, TAHTFPN and TAHTCNB crystal: Excitation position was kept constant and PL signals were collected at different positions. | **S42-S43** |
| **Supplementary Fig. 55-57.** Z-stacking analysis of the MCF-7, confirming the internalization and localization of the TAHOFN in the cytoplasm. (Scale bar: 5 μm). | **S44-S45** |
| **Supplementary Fig. 58.** Celluler internalization mechanism studies using co-crystals. | **S45** |
| **Supplementary Table 24.** A brief summary of the previous report on various optically waveguide active molecular crystals | **S46-S49** |
| **Supplementary Fig. 59-62.** An ellipsoid figure of TAHTCNB single crystal structure. | **S50-S51** |
| **Supplementary Table 25-28.** Cartesian coordinates of TAHOFN, TAHTFPN_G, TAHTFPN_O and TAHTCNB used for the TD-DFT calculation at B3LYP/6-31G (d,p) level. | **S52-S60** |
| **Supplementary Reference** | **S61** |

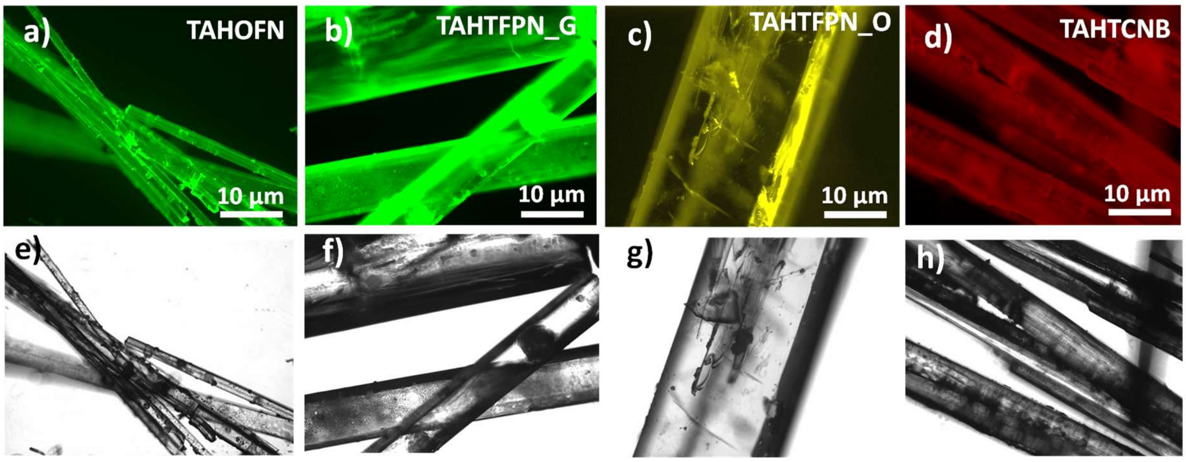


**Supplementary Fig. 1:** (a-d) Fluorescence microscopy images for TAHOFN, TAHTFPN_G, TAHTFPN_O and TAHTCNB co-crystals with the excitation wavelength 405 nm. (e-h) While at the bottom images are their corresponding bright field images (scale bar: 10 µm).

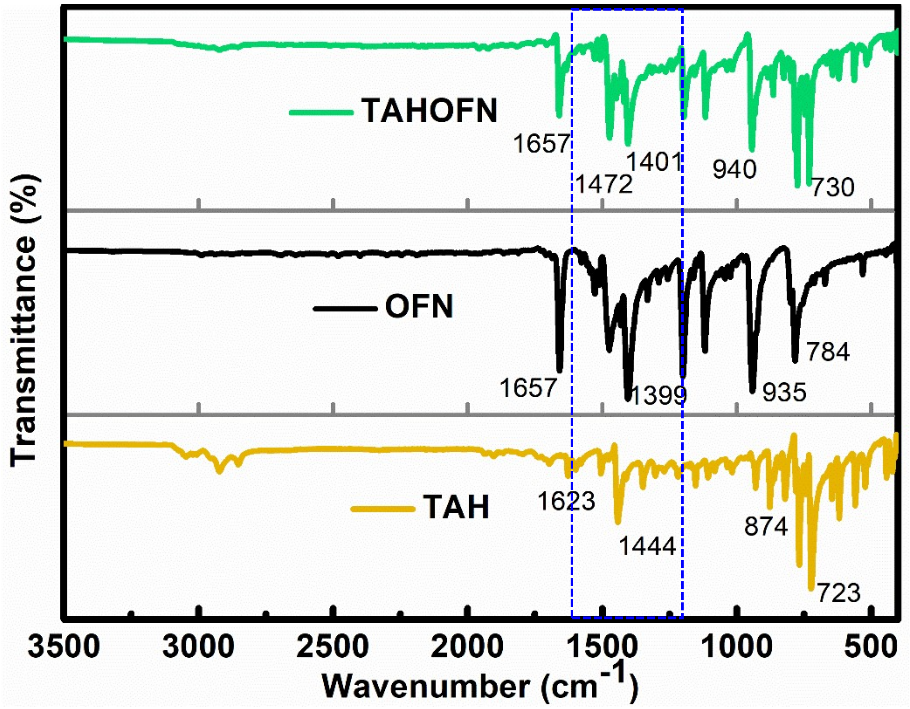


**Supplementary Fig. 2:** FTIR spectra of TAH crystals (yellow line), OFN (black line) and TAHOFN (green line). Here, the C-F stretching vibration peak in OFN at 1399 $cm^{-1}$ became strong and shifted to 1401 $cm^{-1}$, while C-H bending frequency of TAH peaks at 1444 $cm^{-1}$ shifted to 1472 $cm^{-1}$.

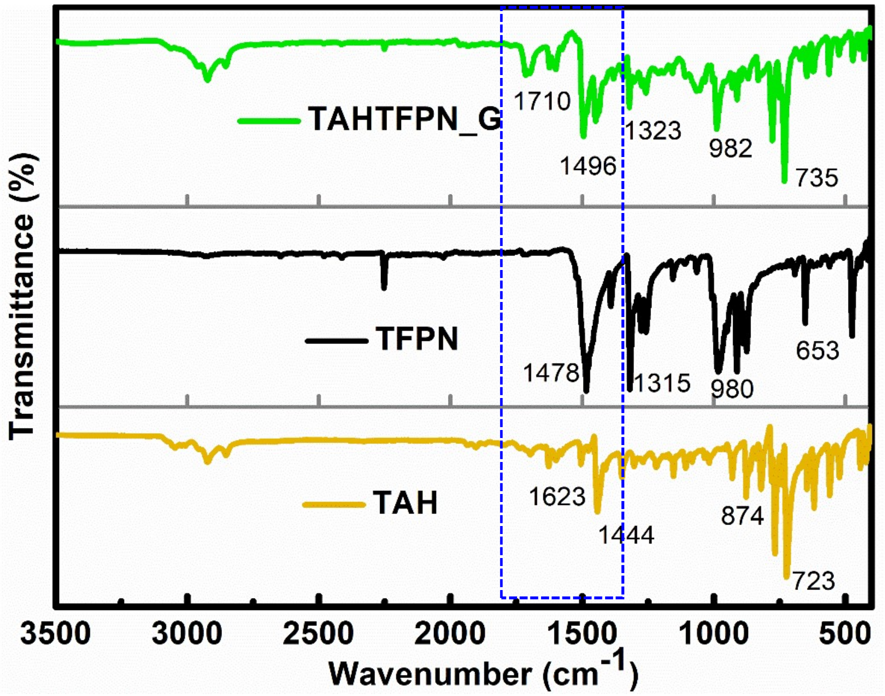


**Supplementary Fig. 3:** FTIR spectra of TAH crystals (yellow line), TFPN (black line) and TAHTFPN_G (green line). Co-crystal TAHTFPN_G, the peak of C-F stretching vibration in TFPN at 1315 cm$^{-1}$ became strong and shifted to 1323 cm$^{-1}$, while C-H bending frequency of TAH in co-crystal, peaks at 1472 cm$^{-1}$ shifted to 1496 cm$^{-1}$.

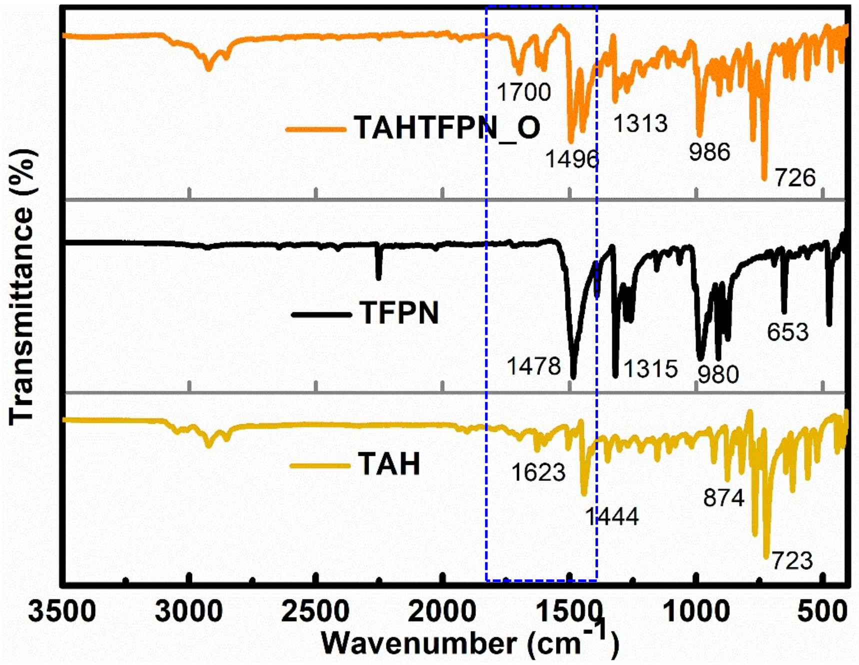


**Supplementary Fig. 4:** FTIR spectra of TAH crystals (yellow line), TFPN (black line) and TAHTFPN_O (orange line). Co-crystal TAHTFPN_O, the peak of C-F stretching vibration in TFPN at 1315 cm$^{-1}$ became strong and shifted to 1313 cm$^{-1}$ while C-H bending frequency of TAH in co-crystal, peaks at 1472 cm$^{-1}$ shifted to 1496 cm$^{-1}$.

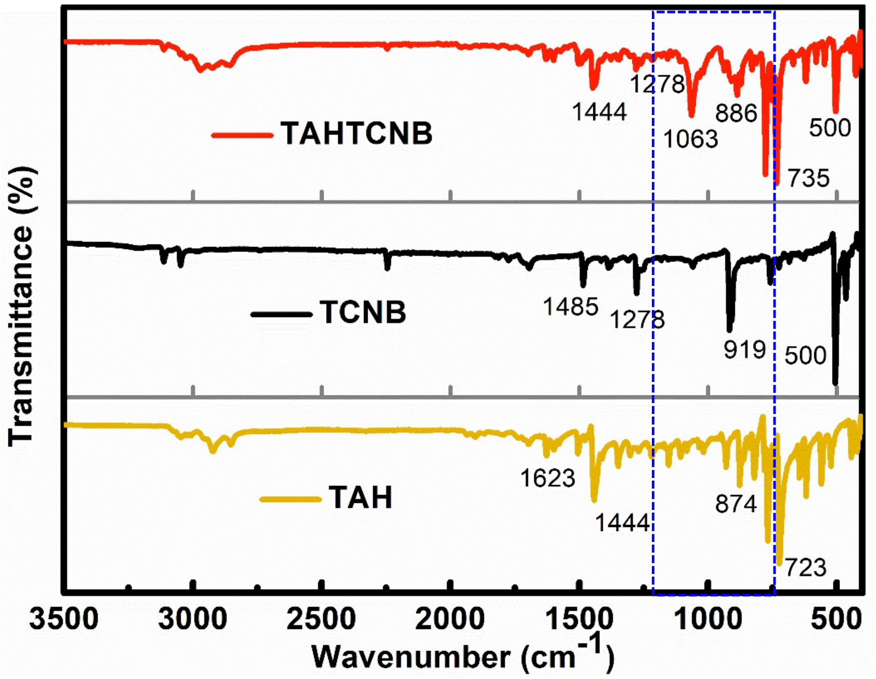


**Supplementary Fig. 5:** FTIR spectra of TAH crystals (yellow line), TCNB (black line) and TAHTCNB (red line).. Co-crystal TAHTCNB no significant CN stretching vibration shift occurred; however, C-H stretching frequency at 919 $cm^{-1}$ shifted to 886 $cm^{-1}$ and became weak. Further, a new peak also appeared at 1063 $cm^{-1,}$ which could be due to the strong CT-complexation.

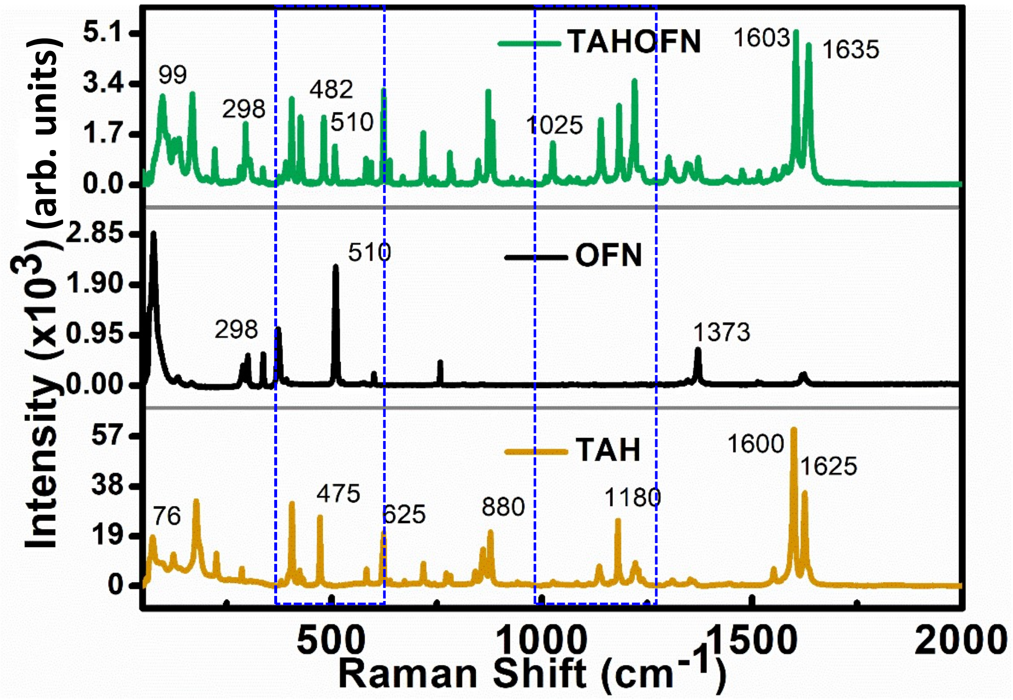


**Supplementary Fig. 6:** Raman spectra of TAH crystals (yellow line), OFN (black line) and TAHOFN (green line).

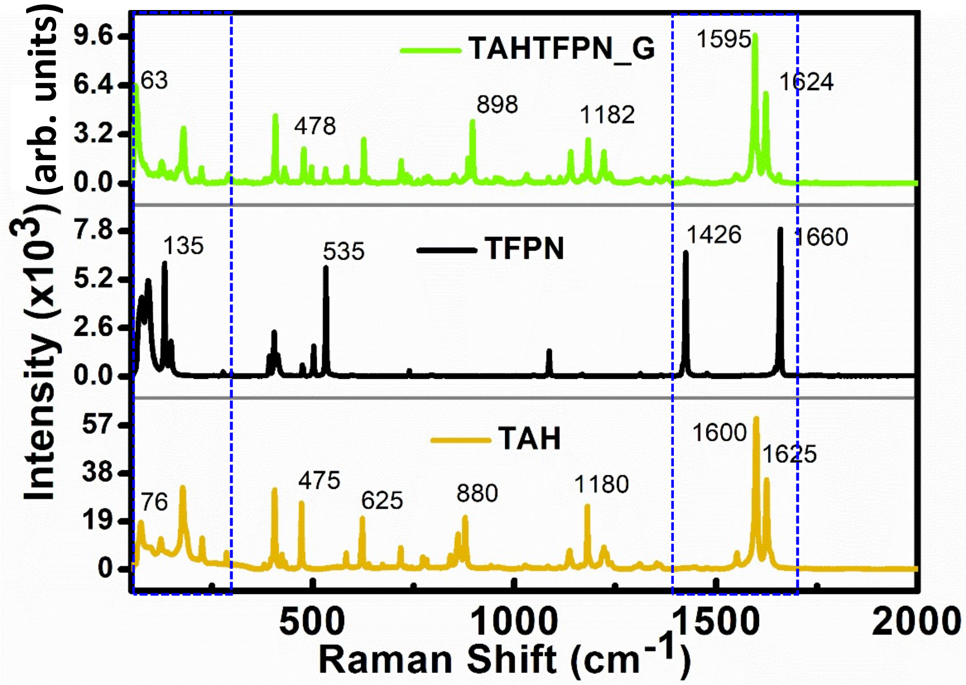


**Supplementary Fig. 7:** Raman spectra of TAH crystals (yellow line), TFPN (black line) and TAHTFPN_G (green line).

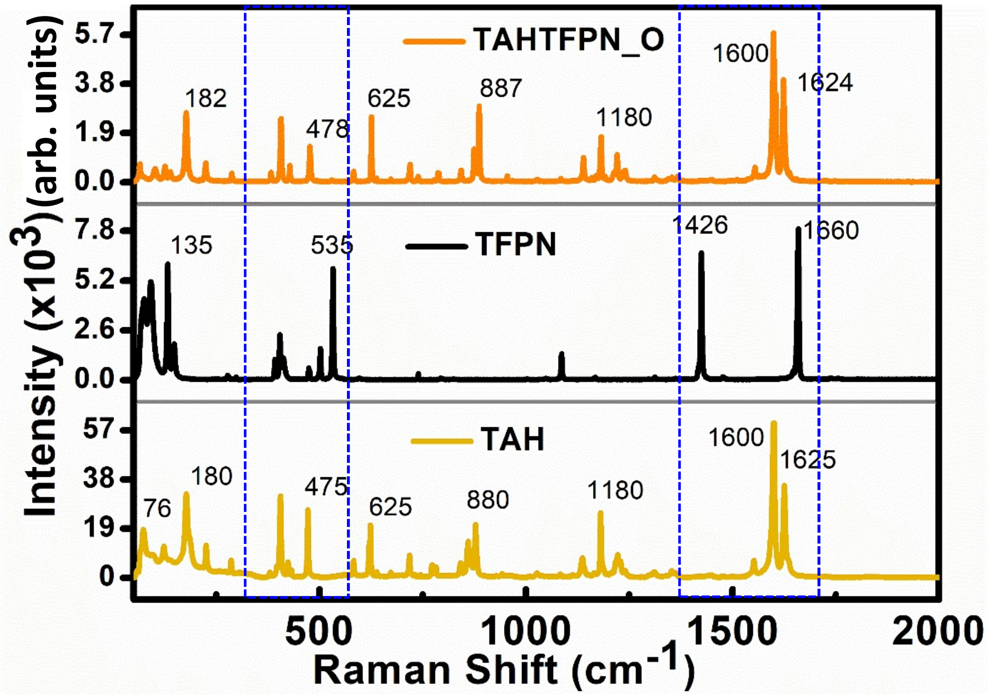


**Supplementary Fig. 8:** Raman spectra of TAH crystals (yellow line), TFPN (black line) and TAHTFPN_O (orange line).

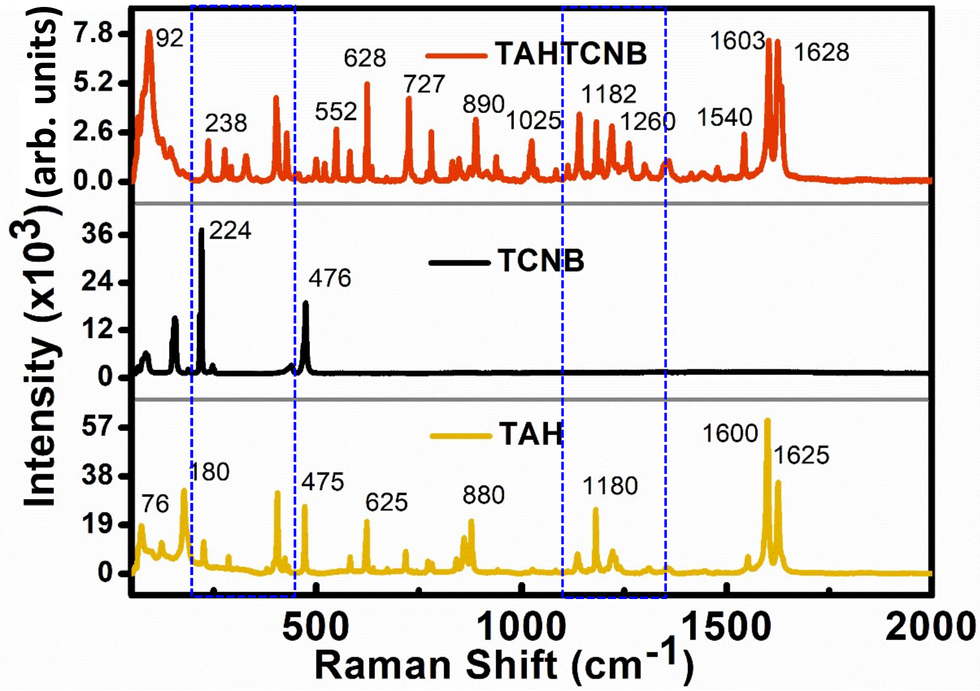


**Supplementary Fig. 9:** Raman spectra of TAH crystals (yellow line), TCNB (black line) and TAHTCNB (red line).

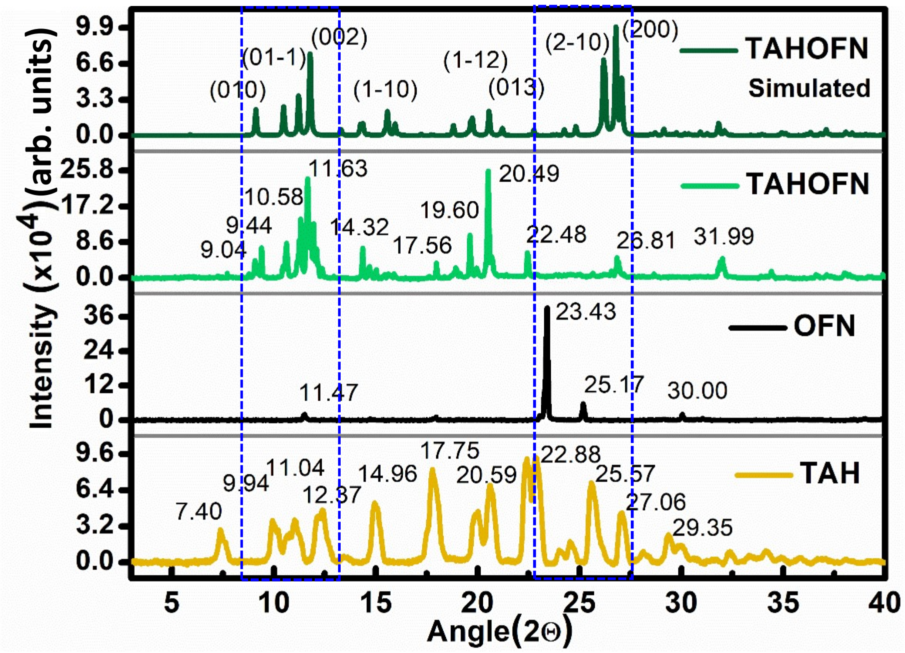


**Supplementary Fig. 10:** Experimental PXRD pattern for TAH crystals (yellow line), OFN (black line), experimental TAHOFN (green line) and simulated TAHOFN (dark green line). Appearance of several new characteristic peaks in TAHOFN co-crystals (9.44°, 11.63°, 14.32°, 19.60°, 22.48°, 31.99°), whereas the absence of several original peaks from pure TAH (7.40°, 12.37°, 17.75°, 22.88°, 27.06°), OFN (23.43°, 25.17°, 30.00°).

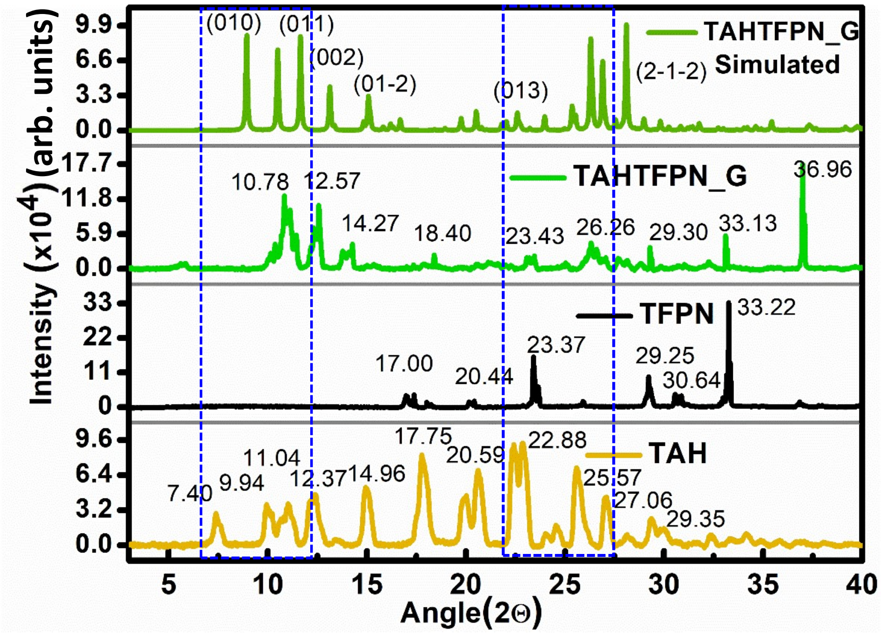


**Supplementary Fig. 11:** Experimental PXRD pattern for TAH crystals (yellow line), TFPN (black line), experimental TAHTFPN_G (green line) and simulated TAHTFPN_G (yellow green line). Appearance of several new characteristic peaks in TAHTFPN_G co-crystals 10.78°, 12.57°, 18.40°, 26.26°, 36.96° and the absence of several original peaks from pure TAH (7.40°, 12.37°, 17.75°, 22.88°, 27.06°) and TFPN (17.00°, 23.37°, 33.22°).

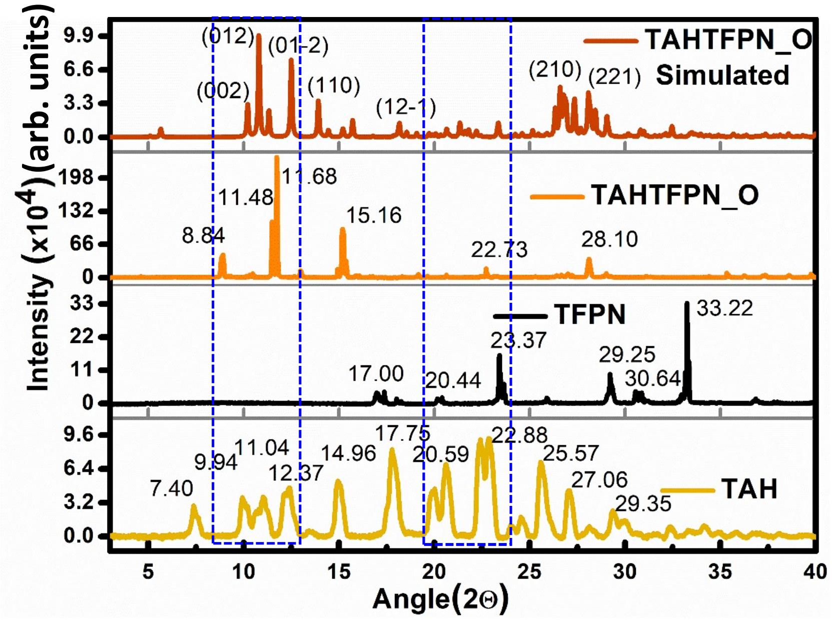


**Supplementary Fig. 12:** Experimental PXRD pattern for TAH crystals (yellow line), TFPN (black line), experimental TAHTFPN_O (orange line) and simulated TAHTFPN_O (dark yellow line). Appearance of several new characteristic peaks in TAHTFPN_O co-crystals at 8.84°, 11.48°, 11.68°, 15.16°, 22.73°, 28.10° and the absence of several original peaks from pure TAH (7.40°, 12.37°, 17.75°, 22.88°, 27.06°), and TFPN (17.00°, 23.37°, 33.22°).

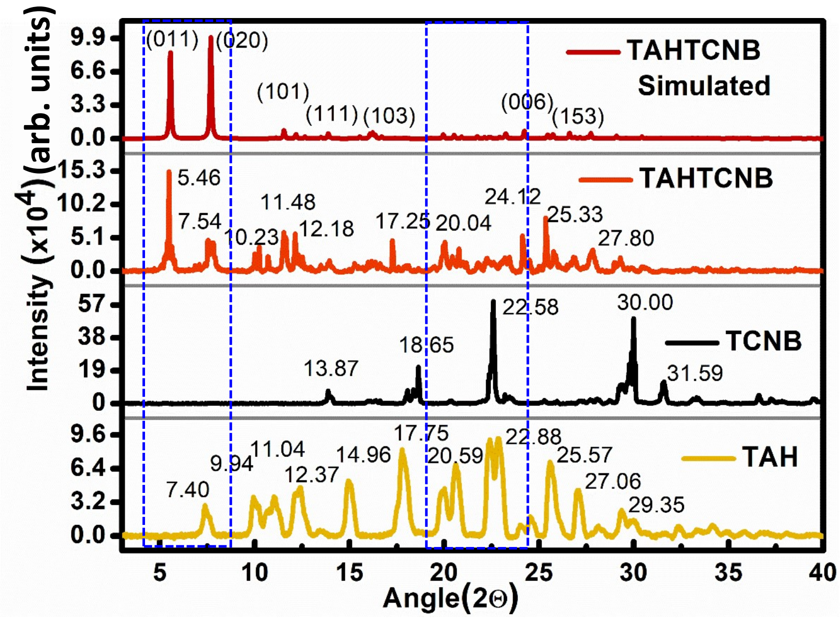


**Supplementary Fig. 13:** Experimental PXRD pattern for TAH crystals (yellow line), TCNB (black line), experimental TAHTCNB (red line) and simulated TAHTCNB (dark red line). Appearance of several new characteristic peaks in TAHTCNB co-crystals of 5.46°, 7.54°, 11.48°, 17.25°, 24.12°. 27.80° and the absence of several original peaks from pure TAH (7.40°, 12.37°, 17.75°, 22.88°, 27.06°), and TCNB (18.65°, 22.58°, 30.00°).

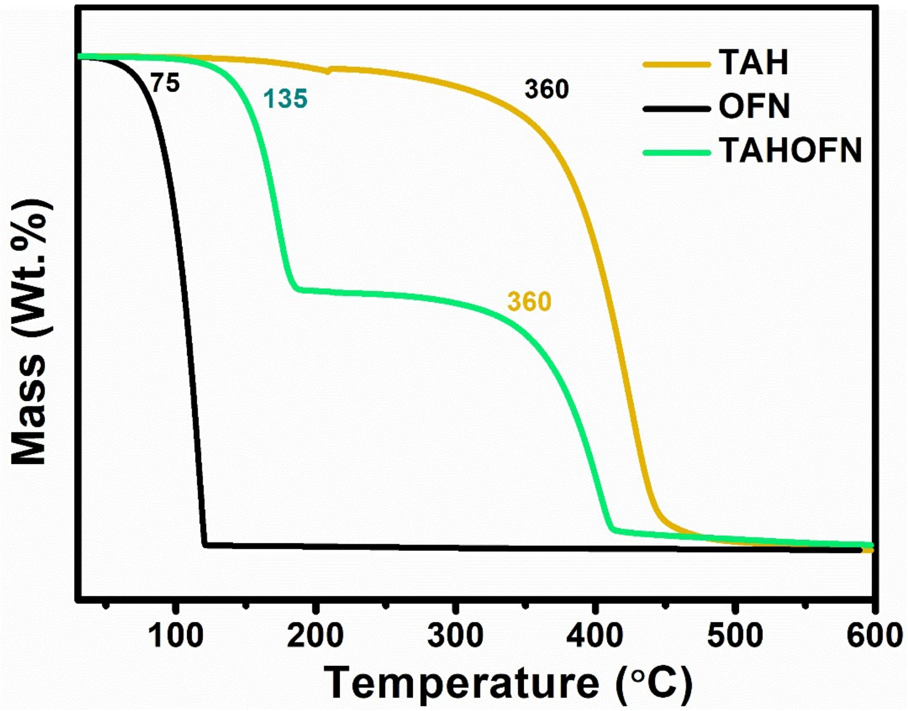


**Supplementary Fig. 14:** Thermal gravimetric analysis (TGA) for TAH (yellow line), OFN (black line) and TAHOFN (green line).

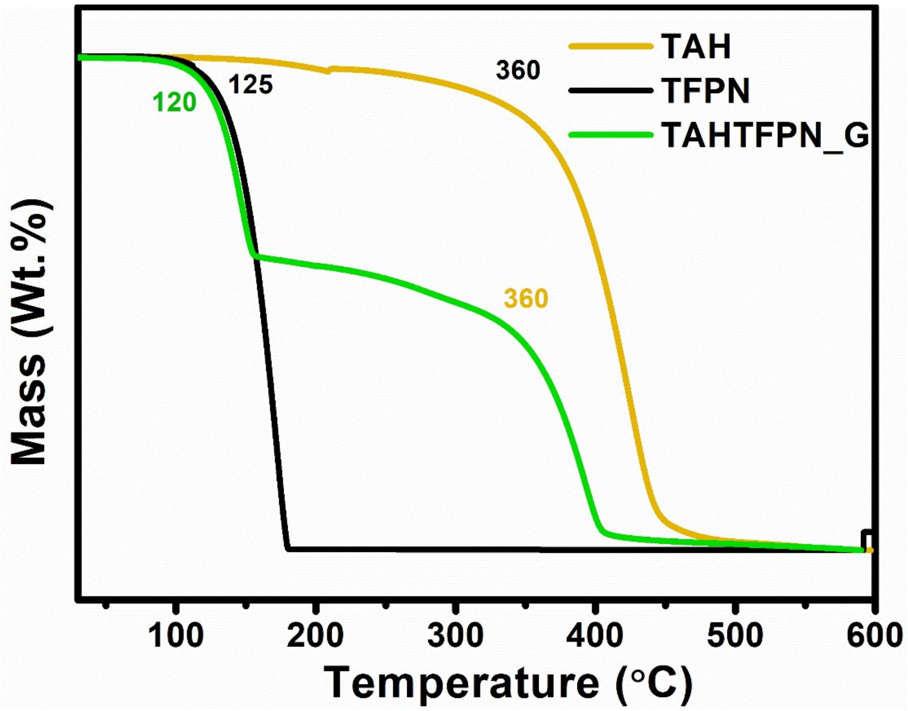


**Supplementary Fig. 15:** Thermal gravimetric analysis (TGA) for TAH (yellow line), TFPN (black line) and TAHTFPN_G (green line).

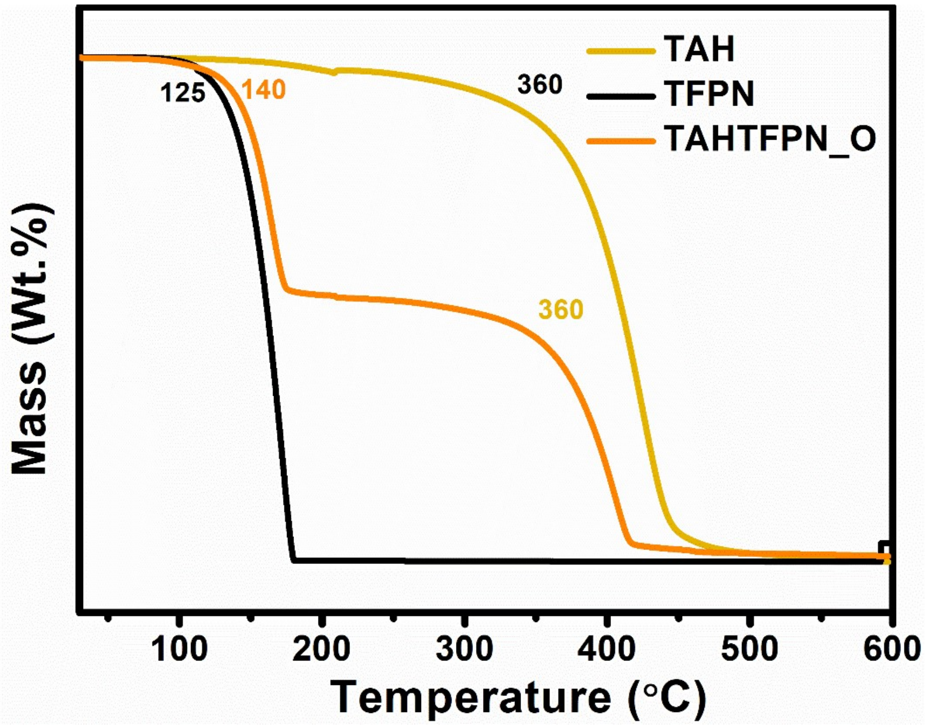


**Supplementary Fig. 16:** Thermal gravimetric analysis (TGA) for TAH (yellow line), TFPN (black line) and TAHTFPN_O (orange line).

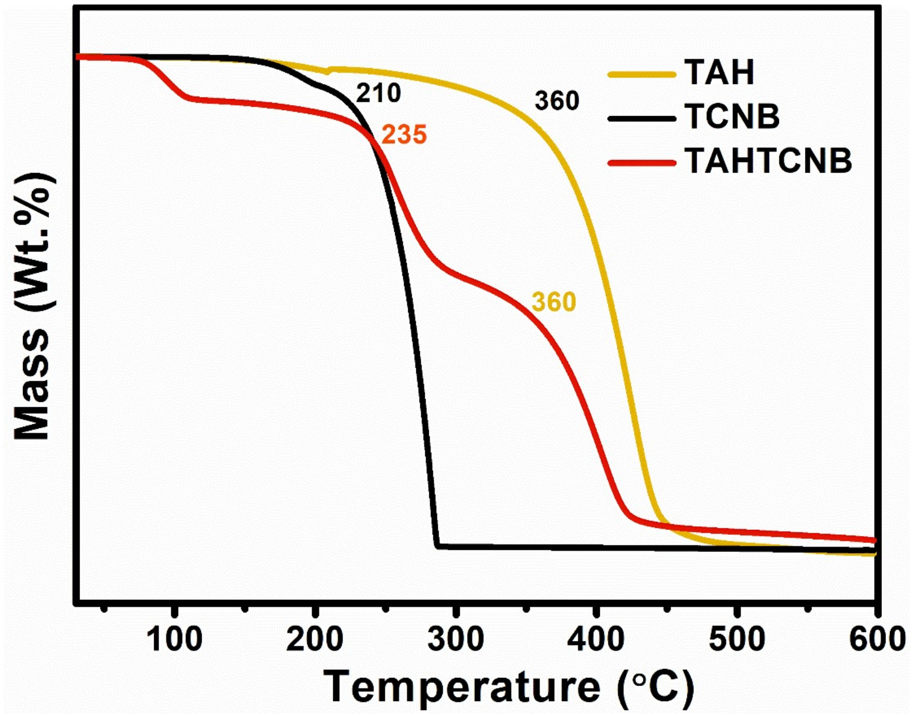


**Supplementary Fig. 17:** Thermal gravimetric analysis (TGA) for TAH (yellow line), TCNB (black line) and TAHTCNB (red line).

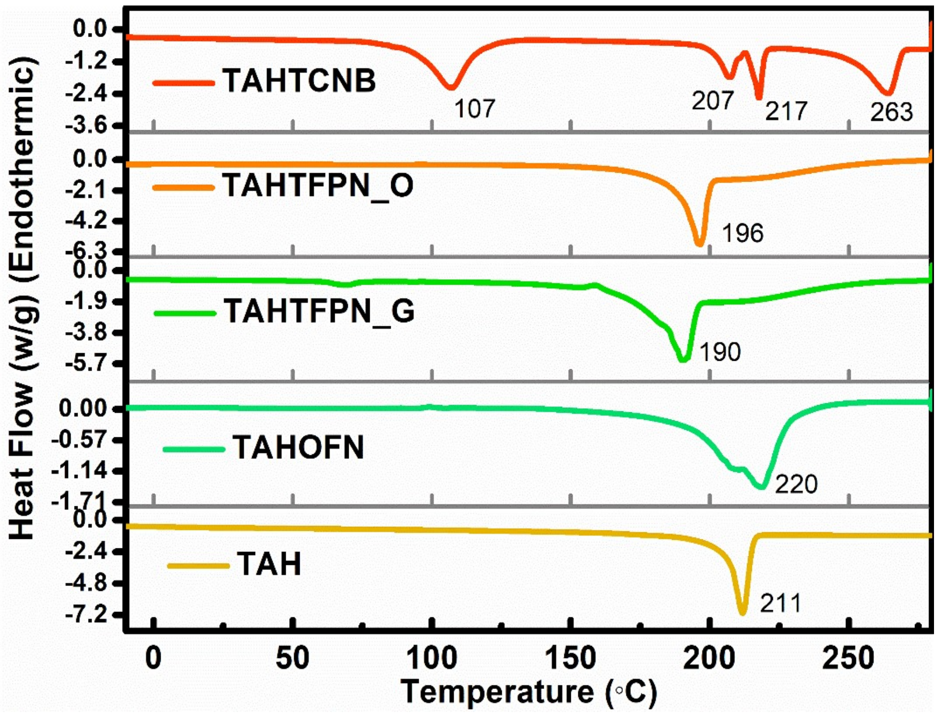


**Supplementary Fig. 18:** Differential scanning calorimetry (DSC) study for TAH (yellow line), TAHOFN (off green line), TAHTFPN_G (green line), TAHTFPN_O (orange line) and TAHTCNB (red line).

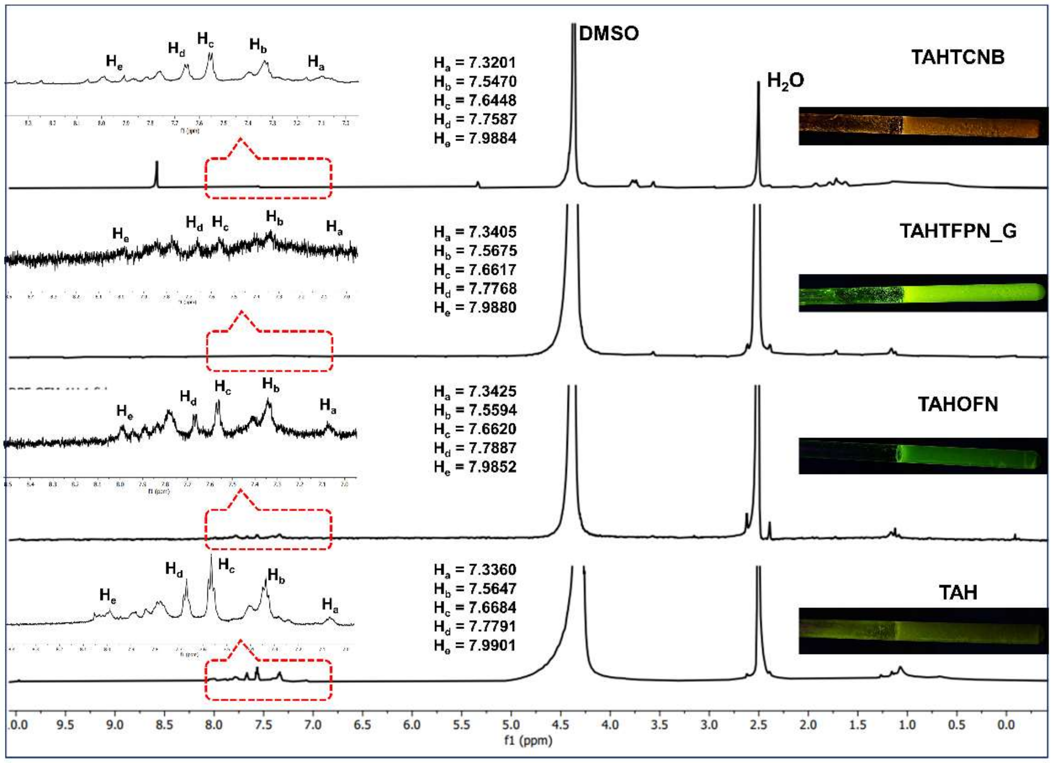


**Supplementary Fig. 19:** Stacked $^1$H NMR spectra for TAHOFN, TAHTFPN and TAHTCNB co-crystals, recorded in DMSO-*d6* and $D_2O$ mixture at 298K. (Inset: images of NMR tubes containing co-crystals taken under 365 nm UV lamp)

**Supplementary Table 1.** Calculated attachment energies of different crystal facets of TAHOFN co-crystal.[1]

| hkl | Dhkl/Å | Eatt(Total)/kcal mol$^{-1}$ | Eatt(vdW)/kcal mol$^{-1}$ | % Total facet area |
|---|---|---|---|---|
| { 0 0 1} | 15.0063956 | -15.18404728 | -13.11742205 | 54.04771092 |
| { 0 1 0} | 9.68802304 | -36.38145248 | -32.67298273 | 15.00237552 |
| { 0 1 1} | 7.8767392 | -32.25494214 | -29.52227144 | 9.5240967 |
| { 1 0 0} | 6.65484113 | -47.72987075 | -42.12068768 | 5.6110776 |
| { 1 0 -1} | 6.58258643 | -45.92078761 | -39.57714372 | 9.48065437 |

**Supplementary Table 2.** Calculated attachment energies of different crystal facets of TAHTFPN_G co-crystal.[1]

| hkl | Dhkl/Å | Eatt(Total)/kcal mol$^{-1}$ | Eatt(vdW)/kcal mol$^{-1}$ | % Total facet area |
|---|---|---|---|---|
| { 0 0 1} | 13.47278056 | -18.0184494 | -15.36076162 | 47.0257642 |
| { 0 1 0} | 9.89148587 | -36.2122139 | -31.10511264 | 17.28027921 |
| { 0 1 1} | 7.59166391 | -30.89557461 | -27.07515935 | 12.45274387 |
| { 1 0 0} | 6.97242549 | -45.30159773 | -36.49290066 | 11.83982621 |
| { 1 0 -1} | 6.6237172 | -44.90672448 | -36.92410463 | 8.8037205 |

**Supplementary Table 3**. Calculated attachment energies of different crystal facets of TAHTFPN_O co-crystal.[1]

| hkl | Dhkl/Å | Eatt(Total)/kcal mol$^{-1}$ | Eatt(vdW)/kcal mol$^{-1}$ | % Total facet area |
|---|---|---|---|---|
| { 0 0 1} | 17.30647421 | -43.66871097 | -32.54464608 | 36.04655729 |
| { 0 1 0 } | 15.60484159 | -33.75079862 | -31.07773417 | 46.67710968 |
| { 1 0 0 } | 6.76187634 | -91.92433018 | -75.2539478 | 16.69519411 |
| { 1 1 1} | 6.12567428 | -104.9041868 | -82.94611497 | 0.58113891 |

**Supplementary Table 4.** Calculated attachment energies of different crystal facets of TAHTCNB co-crystal.[1]

| hkl | Dhkl/Å | Eatt(Total)/kcal mol$^{-1}$ | Eatt(vdW)/kcal mol$^{-1}$ | % Total facet area |
|---|---|---|---|---|
| { 0 1 1} | 15.89762085 | -33.06173254 | -26.3911915 | 85.99938491 |
| { 1 0 1} | 7.65609364 | -109.5026191 | -69.24039722 | 14.00061509 |

**Supplementary Table 5**. Crystallographic data and structure refinement parameters of TAHOFN, TAHTFPN_G, TAHTFPN_O and TAHTCNB co-crystals.

| **Compound** | **TAHOFN** | **TAHTFPN_G** | **TAHTFPN_O** | **TAHTCNB** |
|---|---|---|---|---|
| Empirical formula | $C_{44}H_{22}F_8$ | $C_{42}H_{24}F_4N_2$ | $C_{42}H_{24}F_4N_2$ | $C_{44}H_{24}N_4$ |
| CCDC NO | 2109333 | 2109336 | 2109335 | 2109337 |
| Temperature/K | 293 (2) | 293 (2) | 293 (2) | 293 (2) |
| Crystal system | triclinic | triclinic | triclinic | orthorhombic |
| Space group | P -1 | P -1 | P -1 | P n m a |
| a/Å | 6.9672(6) | 7.0777(7) | 6.777(4) | 8.1658(6) |
| b/Å | 9.9719(12) | 9.9789(12) | 15.869(11) | 22.9815(16) |
| c/Å | 15.3125(11) | 13.6987(13) | 17.567(9) | 22.0149(19) |
| α/° | 91.687(8) | 95.475(9) | 80.15(5) | 90 |
| β/° | 100.664(7) | 98.390(8) | 88.60(4) | 90 |
| γ/° | 103.036(9) | 94.383(9) | 86.25(5) | 90 |
| Volume/Å$^3$ | 1015.72(17) | 948.77(18) | 1857.2(19) | 4131.4(6) |
| Z | 2 | 2 | 2 | 4 |
| $\rho_{calc}$mg/mm$^3$ | 1.595 | 1.454 | 1.514 | 0.979 |
| m/mm$^{-1}$ | 0.144 | 0.114 | 1.022 | 0.058 |
| F(000) | 490 | 422 | 860 | 1264.4830 |
| Index ranges | $-8 \leq h \leq 7, -11 \leq k \leq 11, -18 \leq l \leq 18$ | $-9 \leq h \leq 8, -11 \leq k \leq 13, -18 \leq l \leq 18$ | $-7 \leq h \leq 7, -17 \leq k \leq 17, -19 \leq l \leq 19$ | $-6 \leq h \leq 9, -27 \leq k \leq 13, -17 \leq l \leq 26$ |
| Reflections collected | 6600 | 7707 | 44322 | 10016 |
| Independent reflections | 3593[R(int) = 0.0726] | 4310[R(int) = 0.0799] | 5355[R(int) = 0.1135] | 3732 |
| Data/restraints/parameters | 3593/0/316 | 4310/0/280 | 5355/0/559 | 3732/0/217 |
| Goodness-of-fit on F$^2$ | 1.060 | 1.081 | 1.238 | 1.0477 |
| Final R indexes [I>=2σ (I)] | R1 = 0.0692, wR2 = 0.1610 | R1 = 0.0847, wR2 = 0.2073 | R1 = 0.1426, wR2 = 0.2831 | R1 = 0.1207, wR2 = 0.3363 |
| Final R indexes [all data] | R1 = 0.1424, wR2 = 0.2120 | R1 = 0.1307, wR2 = 0.2466 | R1 = 0.1520, wR2 = 0.2888 | R1 = 0.1796, wR2 = 0.3792 |

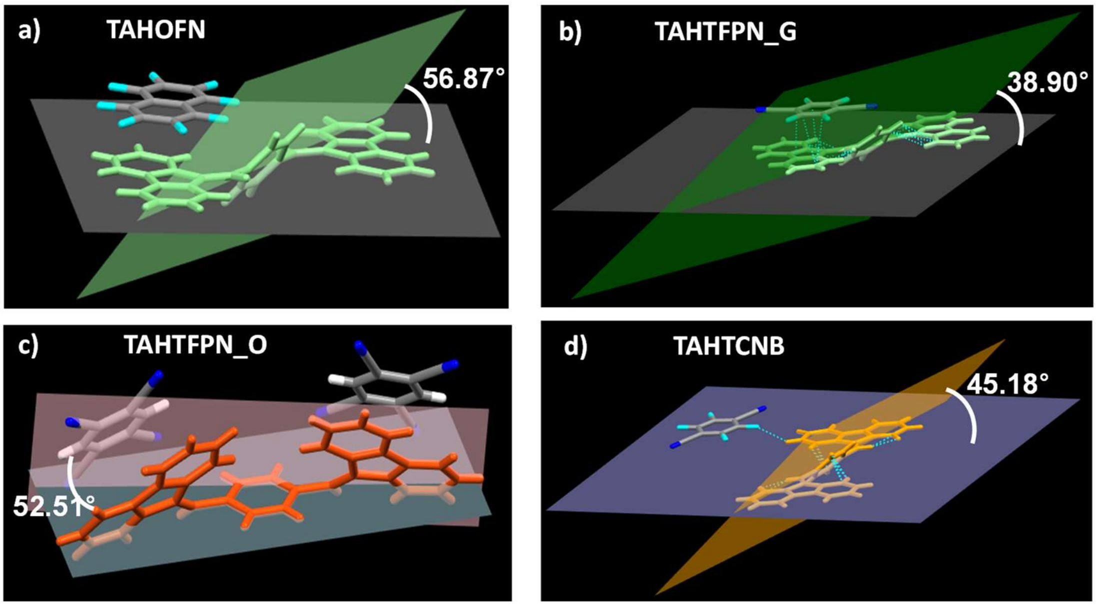


**Supplementary Fig. 20:** Twisted angle between two planes of planar TAH and central phenyl planes in difference interactive orientation to the guest molecules (OFN, TFPN and TCNB) resulted in variable conformations of TAH, revealing polymorphism features of all the afforded co-crystals of (a) TAHOFN, (b) TAHTFPN_G, (c) TAHTFPN_O, (d) TAHTCNB.

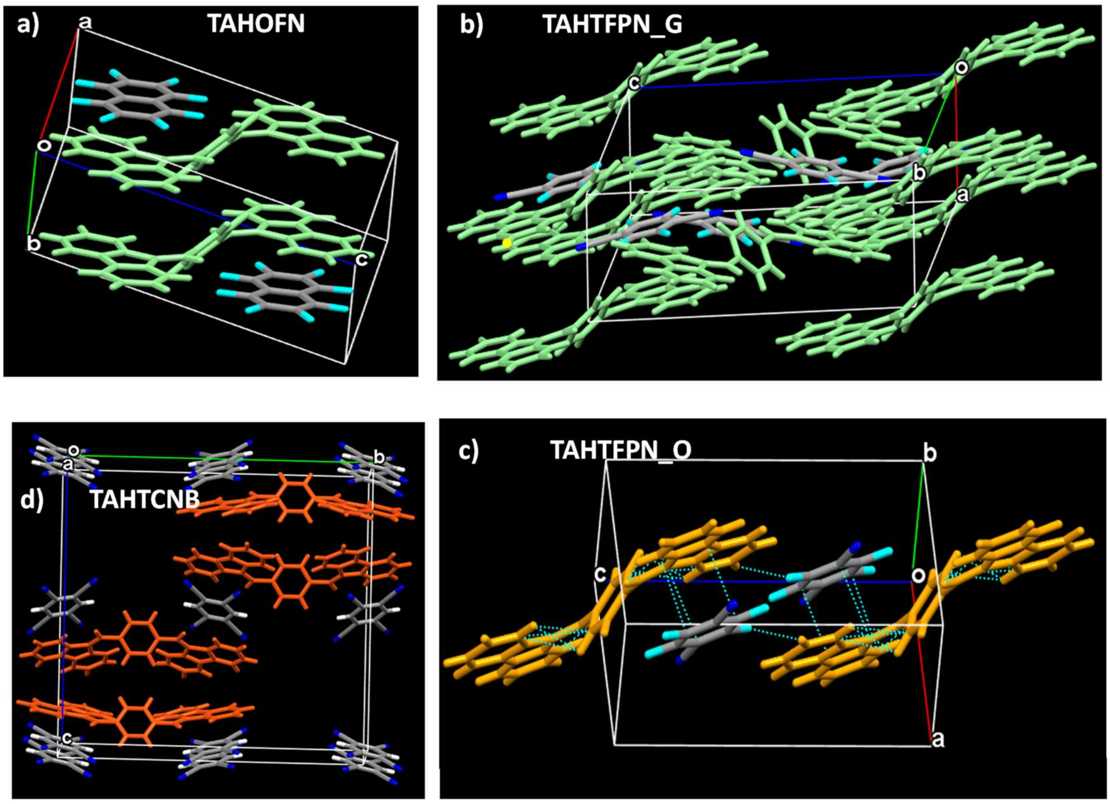


**Supplementary Fig. 21:** Unit cell packing in the SC-XRD structure for (a) TAHOFN, (b) TAHTFPN_G, (c) TAHTFPN_O, (d) TAHTCNB showing close contact with mixed stack and segregated stack alignment between TAH and OFN, TFPN and TCNB cores.

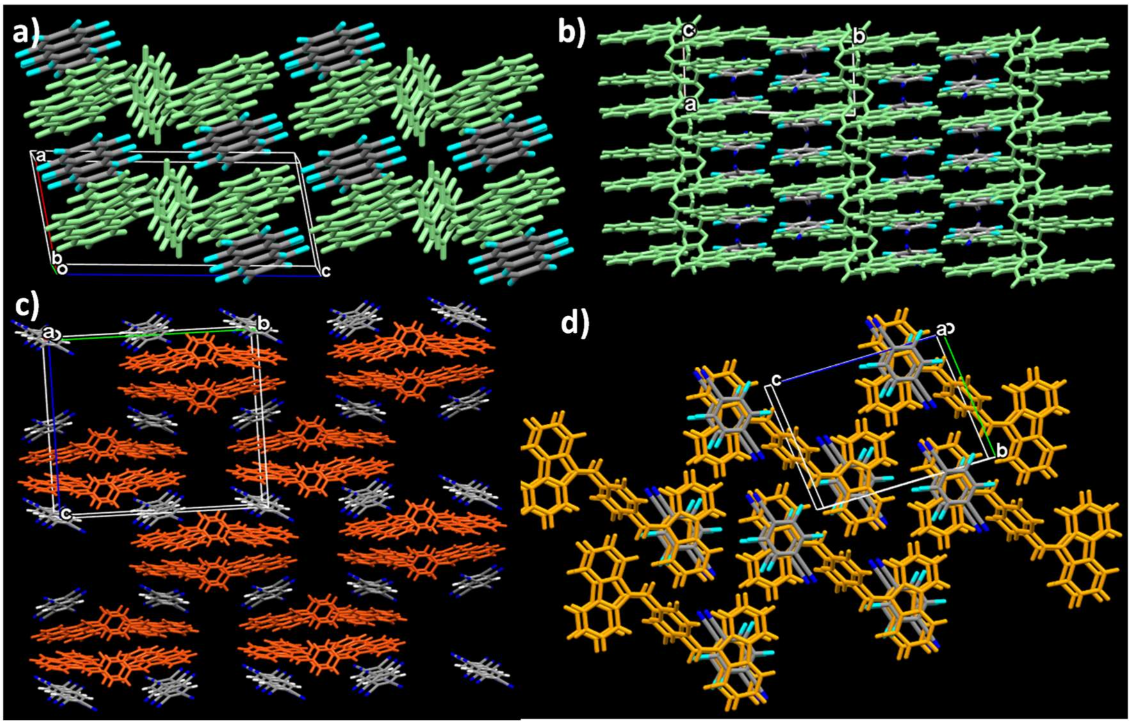


**Supplementary Fig. 22: (a-d)** Supramolecular rigid 3D network assembly via charge transfer interactions of (a) TAHOFN, (b) TAHTFPN_G, (c) TAHTFPN_O and (d) cis-TAHTCNB co-crystals respectively. Difference interactive orientation to the guest molecules resulted in variable conformations of TAH, revealing polymorphism features of all the afforded co-crystals.

**Supplementary Table 6.** SCXRD-crystal structural analysis with non-covalent interactions and dihedral angle for all the afforded co-crystals.

| **Single Crystals** | **Inter-molecular Interactions Close contact distance (Å)** | | | | | **Intramolecular Interactions Close contact distance (Å)** | | | **Dihedral Angle (Degree)** | |
|---|---|---|---|---|---|---|---|---|---|---|
| | **CH-F** | **CH-N** | **CH-π** | **D-Aπ-π** | **D-Dπ-π** | **F-F** | **CH-N** | **CH-π** | **Inter D-A** | **Intra In TAH** |
| **TAHOFN** | 2.54 | - | - | 3.40 | 6.96 | 2.56 | 2.663 | 2.37<br>2.83 | 88.7 | 50.10 |
| **TAHTFPN_G** | - | 2.35 | 2.39 | 3.54 | 7.07 | - | - | 2.74<br>2.81 | 80.10 | 40.92 |
| **TAHTFPN_O** | 2.60 | 2.58 | 3.37 | 3.65 | 6.77 | - | - | 2.55<br>2.72 | 76.91 | 39.30 |
| **TAHTCNB** | - | 2.59 | 3.38 | 3.73 | 7.47 | - | - | 2.82<br>2.83 | 76.03 | 46.10 |

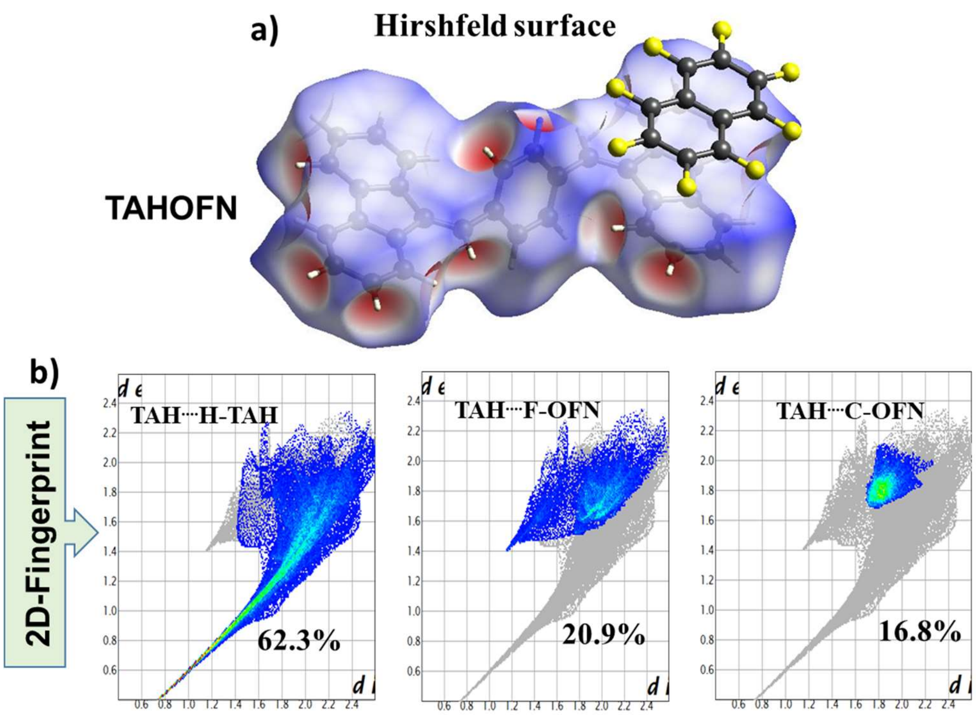


**Supplementary Fig. 23:** a) Hirshfeld surface images of TAHOFN co-crystal (major surface mapped on TAH). b) 2D Finger print for surface to atom intermolecular interactions mapped on Hirshfeld surface in TAHOFN.[2,3]

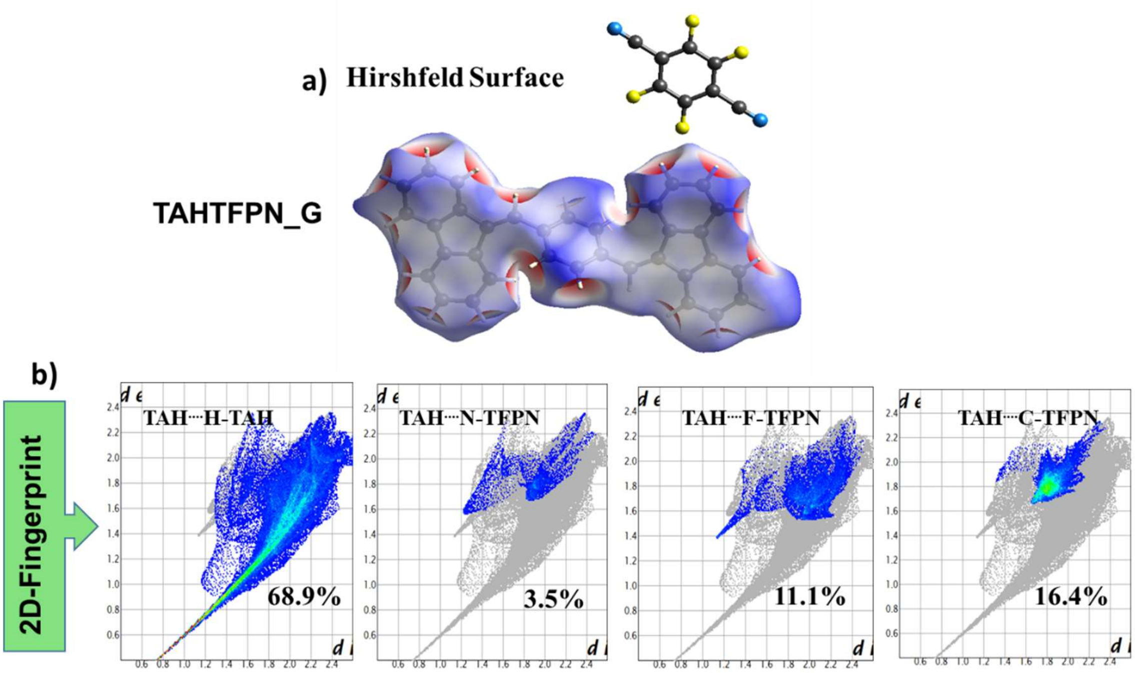


**Supplementary Fig. 24:** a) Hirshfeld surface images of TAHTFPN_G co-crystal (major surface mapped on TAH). b) 2D Finger print for surface to atom intermolecular interactions mapped on Hirshfeld surface in TAHTFPN_G.[2,3]

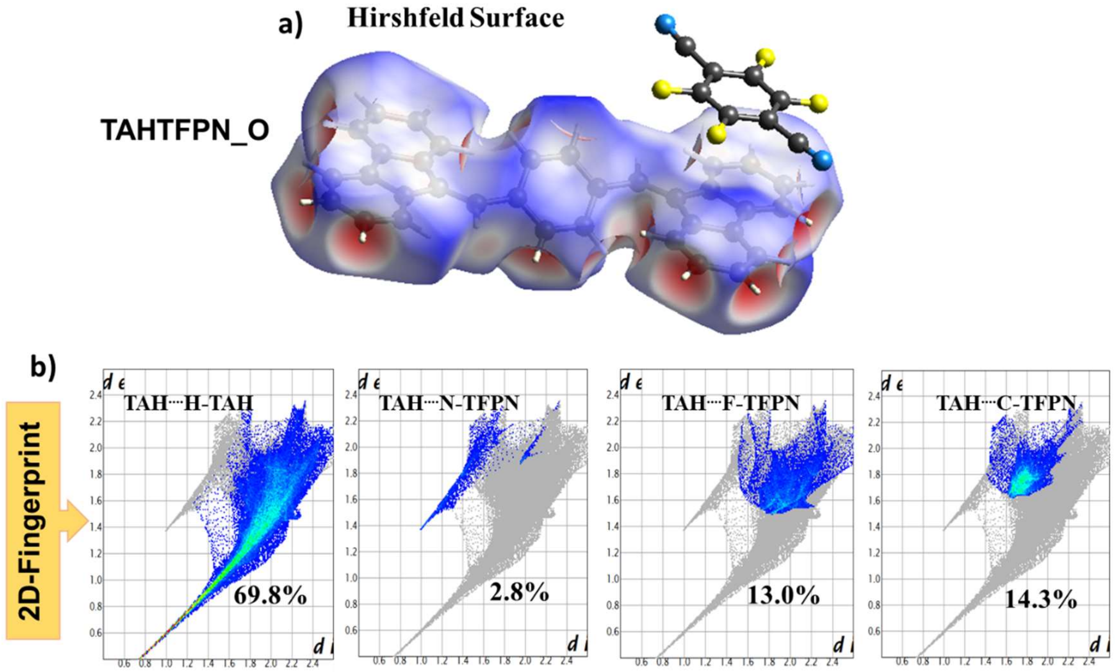


**Supplementary Fig. 25:** a) Hirshfeld surface images of TAHTFPN_O co-crystal (major surface mapped on TAH). b) 2D Finger print for surface to atom intermolecular interactions mapped on Hirshfeld surface in TAHTFPN_O.[2,3]

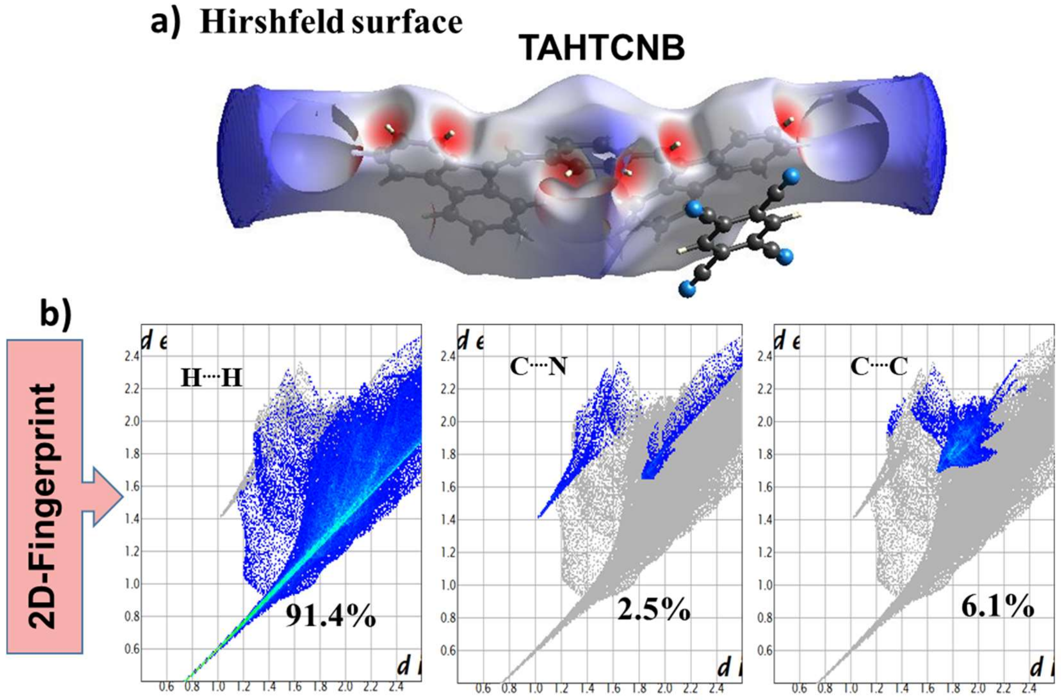


**Supplementary Fig. 26:** a) Hirshfeld surface images of TAHTCNB co-crystal (major surface mapped on TAH). b) 2D Finger print for surface to atom intermolecular interactions mapped on Hirshfeld surface in TAHTCNB.[2,3]

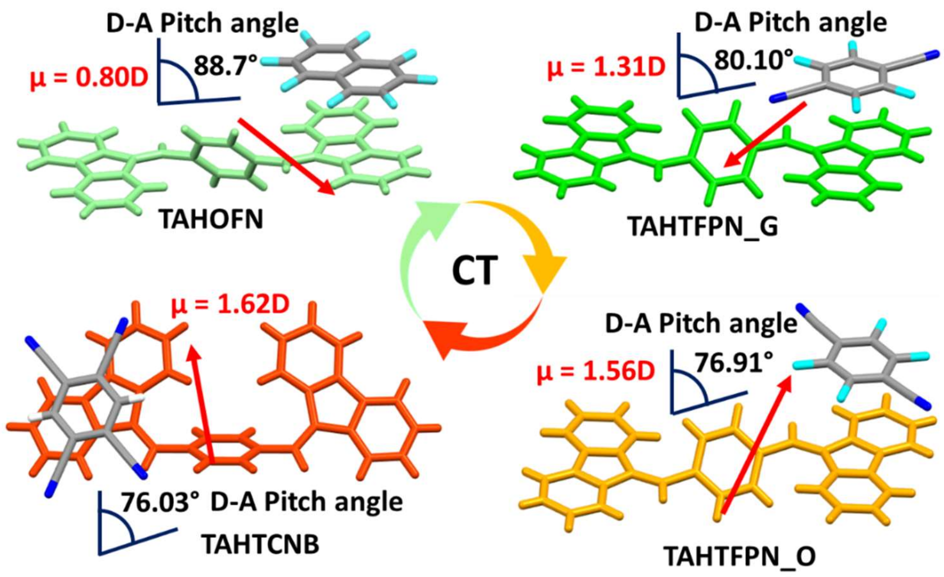


**Supplementary Fig. 27:** Orientation of dipole moment (μ) and pitch angles between D-A obtained from TD-DFT and SC-XRD structures of TAHOFN, TAHTFPN_G, TAHTFPN_O and TAHTCNB.

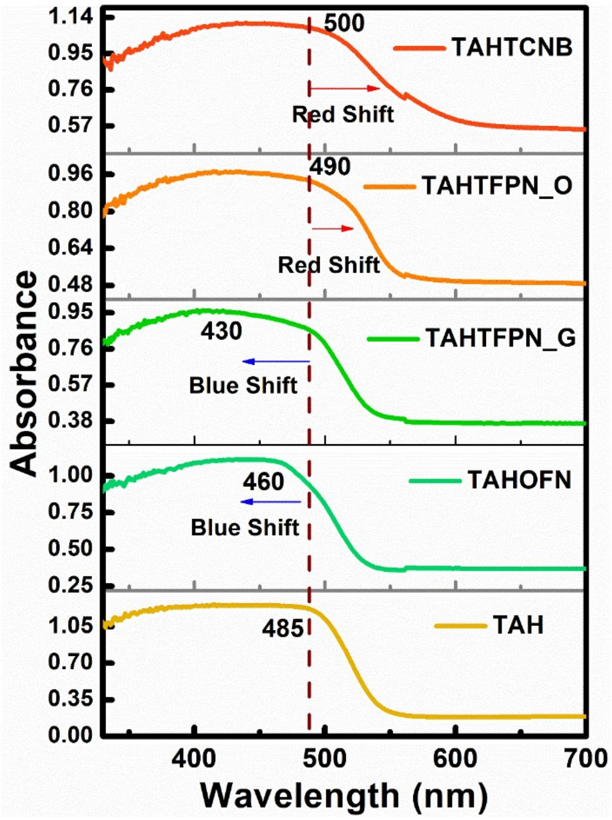


**Supplementary Fig. 28:** Diffuse reflection absorption spectra of TAH (yellow line), TAHOFN (off green line), TAHTFPN_G (green line), TAHTFPN_O (orange line) and TAHTCNB (red line). The absorption spectra at condensed state of the luminogens exhibited single broad absorption band in water at 400 nm and in THF at 364 nm (d).

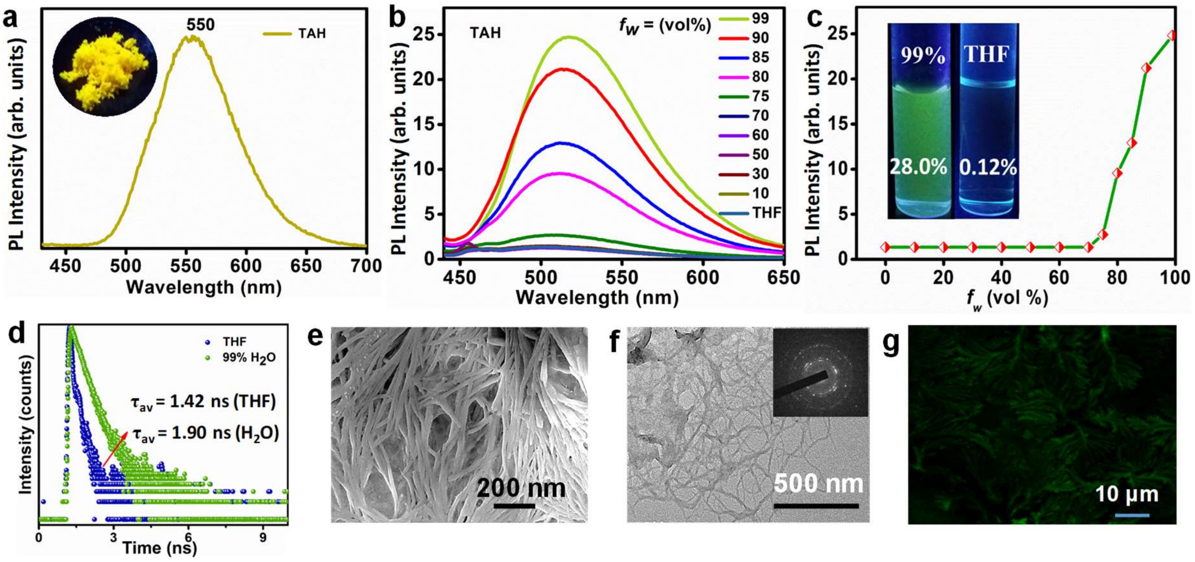


**Supplementary Fig. 29:** Photophysical morphological characterization of TAH: (a) Photoluminescence spectra for solid pristine TAH. (b) Aggregation–induced emission at different fractions of THF and water. (c) Relative intensity at various fractions of THF/water (inset aggregation images taken under 365 nm UV lamp and absolute PLQY). (d) TRPL decay in THF and water. (e-g) FESEM, FETEM and fluorescence images of TAH nano-ribbons formed in water. (Adapted with permission from ref 42 cited in the main MS @Copyright 2021 American Chemical Society).

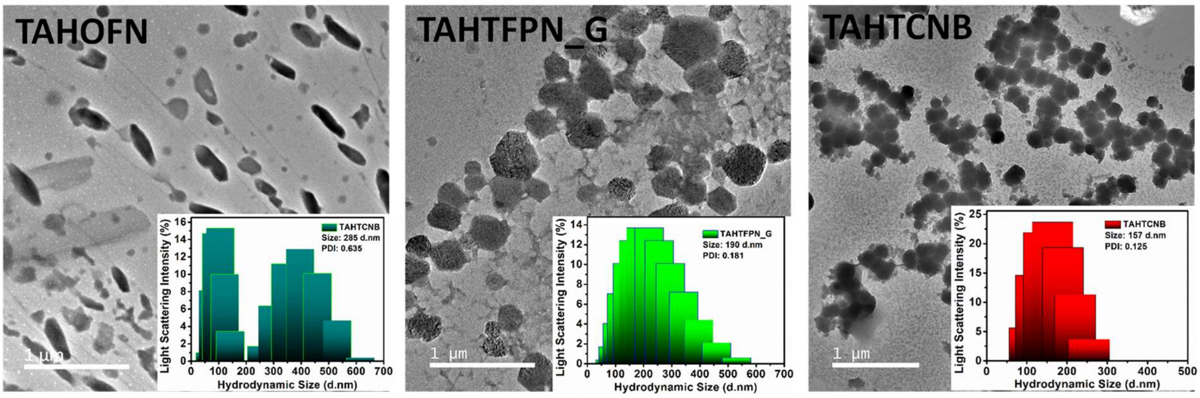


**Supplementary Fig. 30:** FETEM images (inset: DLS graph for size distribution) of co-crystal nano aggregates formed in DMSO/$H_2O$ (99%, $f_w$) for TAHOFN, TAHTFPN_G and TAHTCNB.

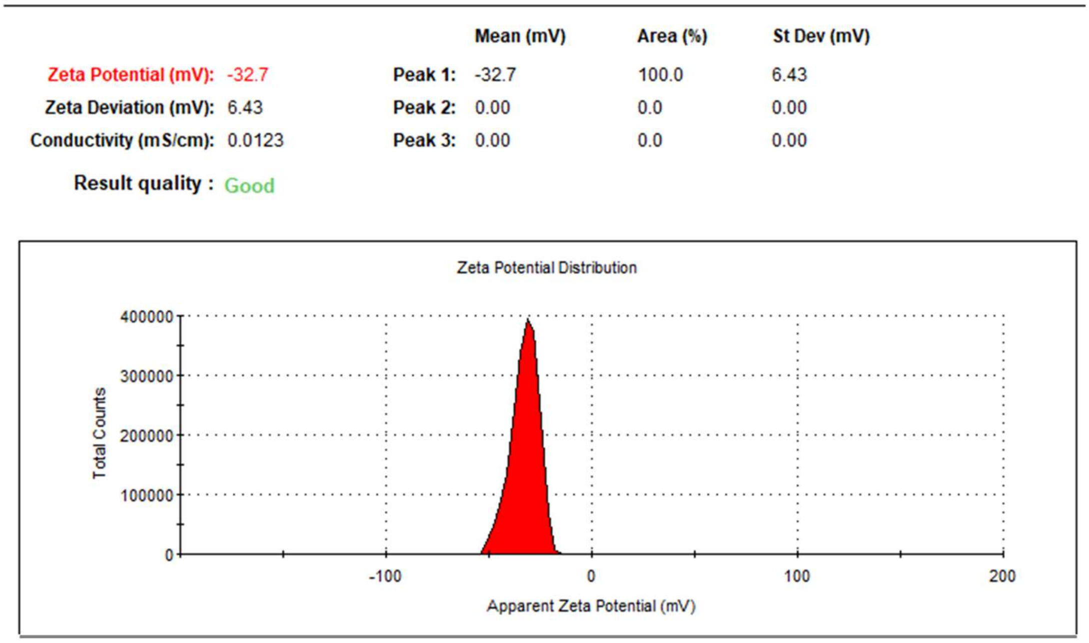


**Supplementary Fig. 31:** Zeta-potential recorded in $H_2O$ for TAHOFN at $10^{-5}$ M concentration.

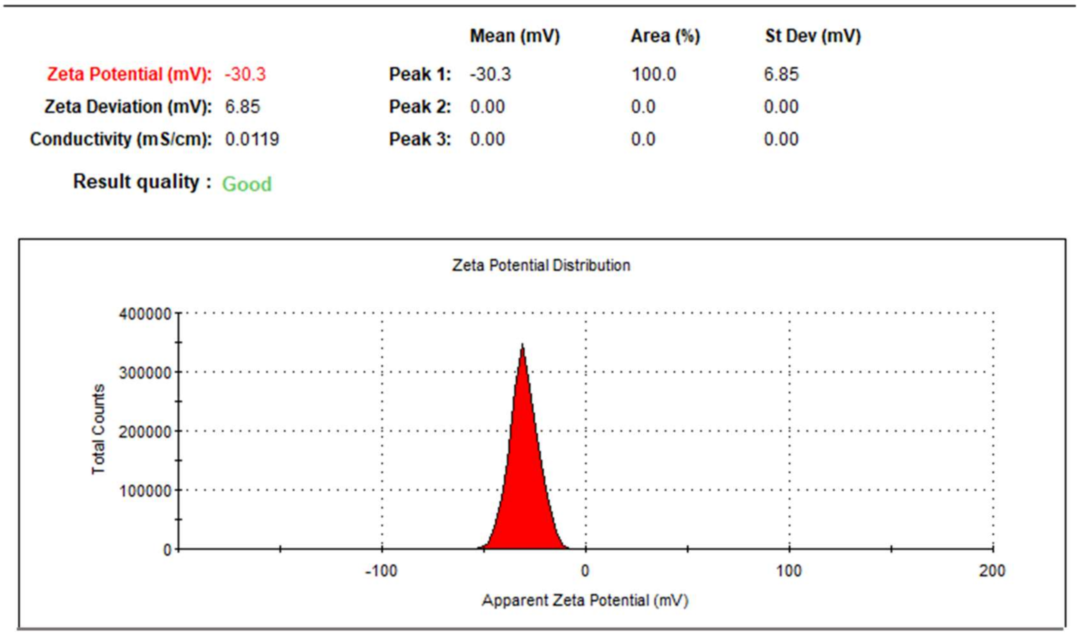


**Supplementary Fig. 32:** Zeta-potential recorded in $H_2O$ for TAHTFPN_G at $10^{-5}$ M concentration.

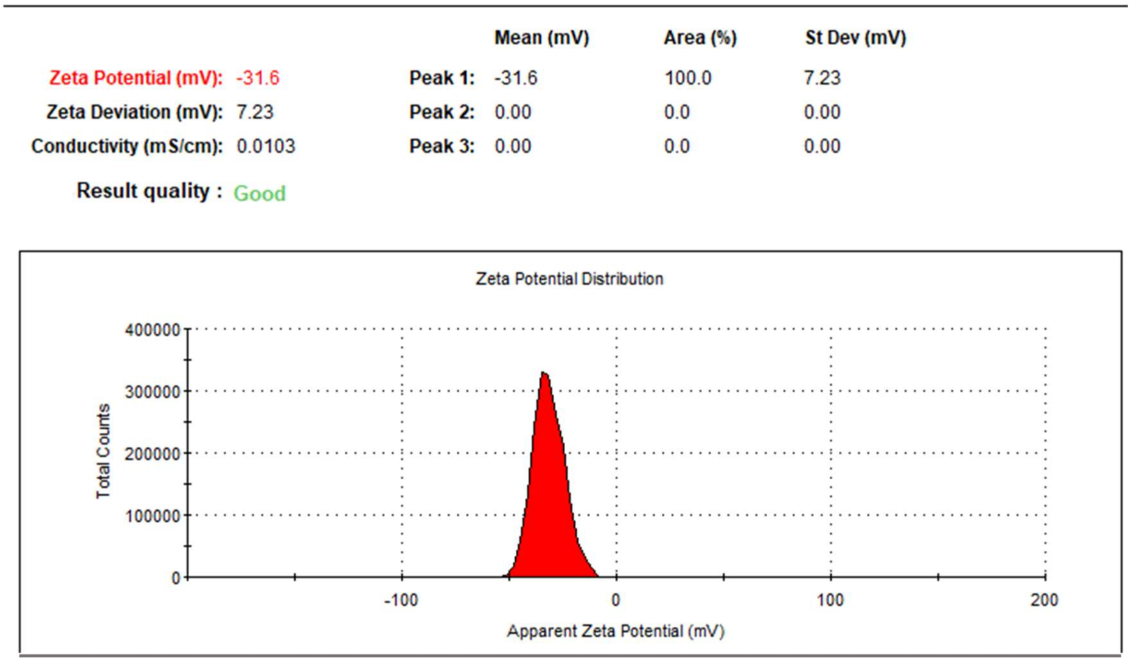


**Supplementary Fig. 33:** Zeta-potential recorded in $H_2O$ for TAHTCNB at $10^{-5}$ M concentration.

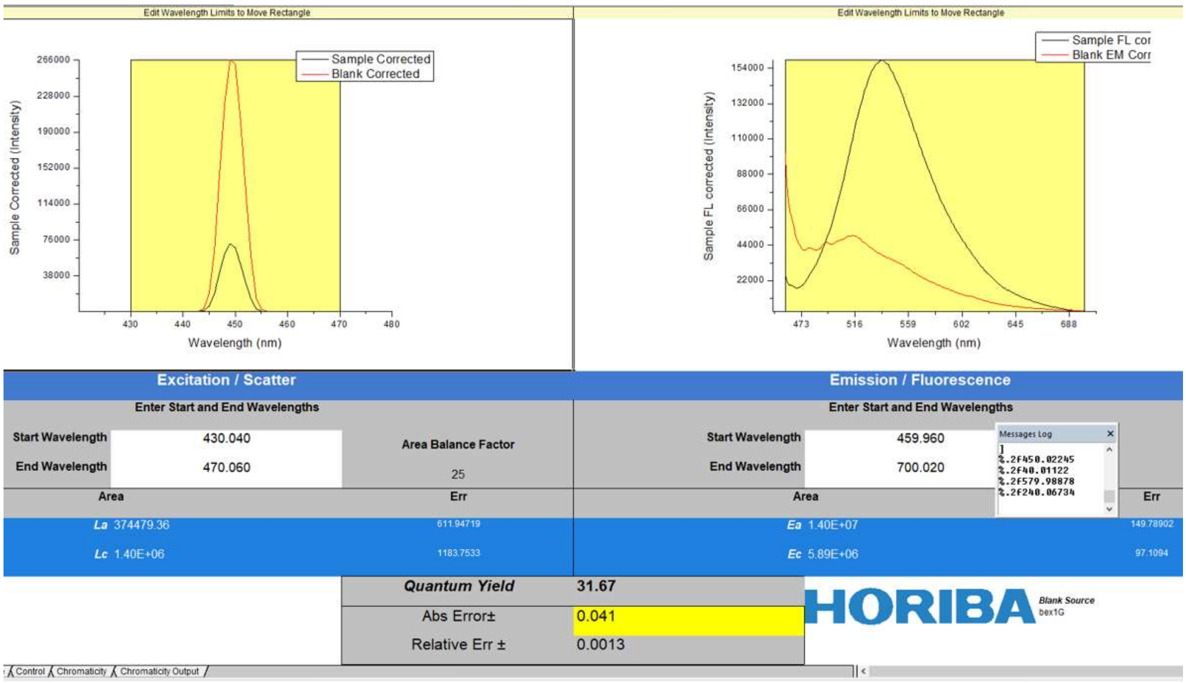


**Supplementary Fig. 34:** PLQY of TAHOFN co-crystal in solid state measured in the absolute method using integrating sphere.

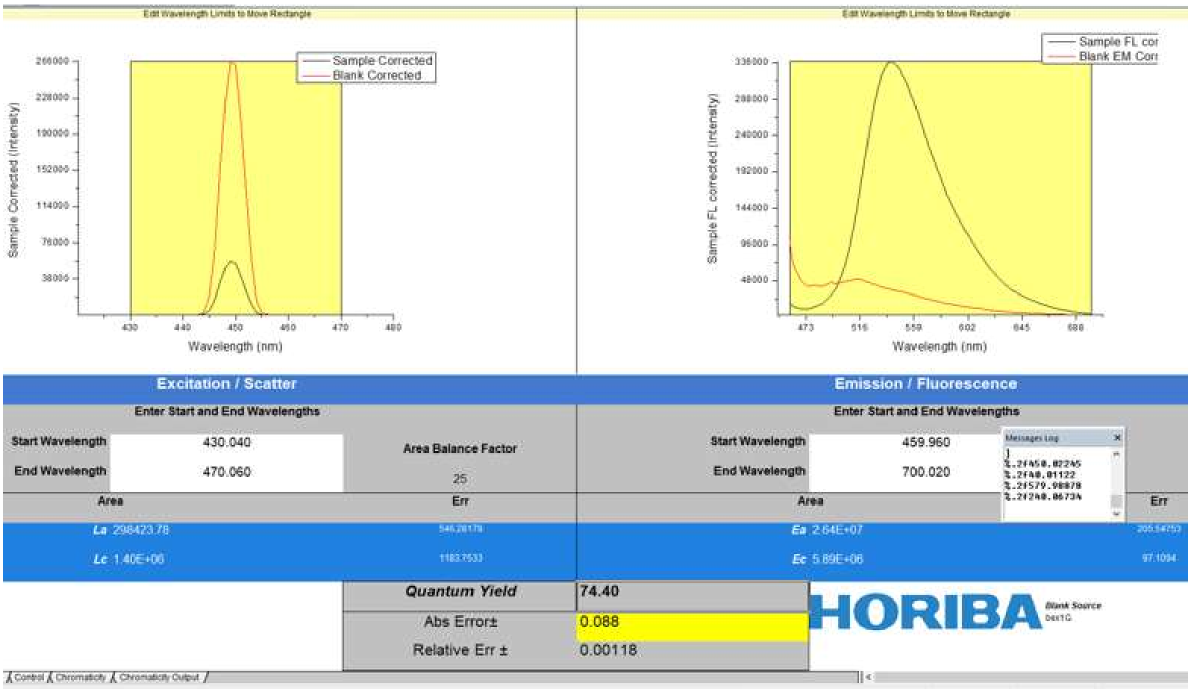


**Supplementary Fig. 35:** PLQY of TAHTFPN_G co-crystal in solid state measured in the absolute method using integrating sphere.

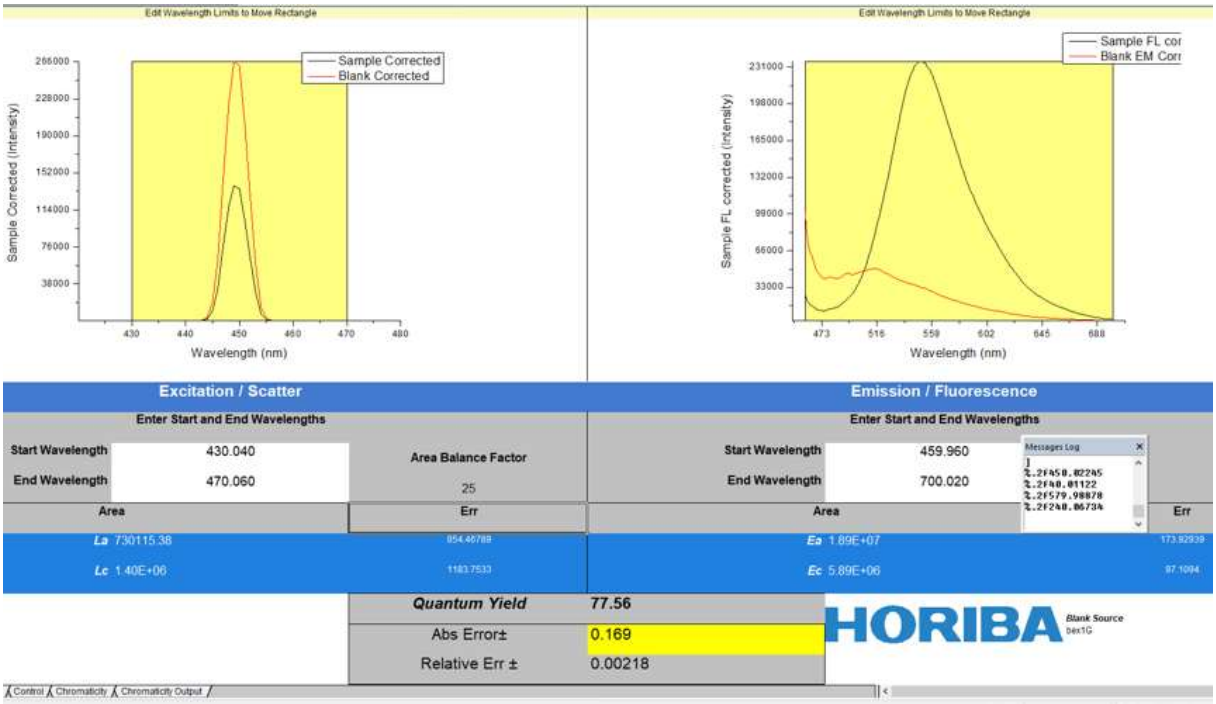


**Supplementary Fig. 36:** PLQY of TAHTFPN_O co-crystal in solid state measured in the absolute method using integrating sphere.

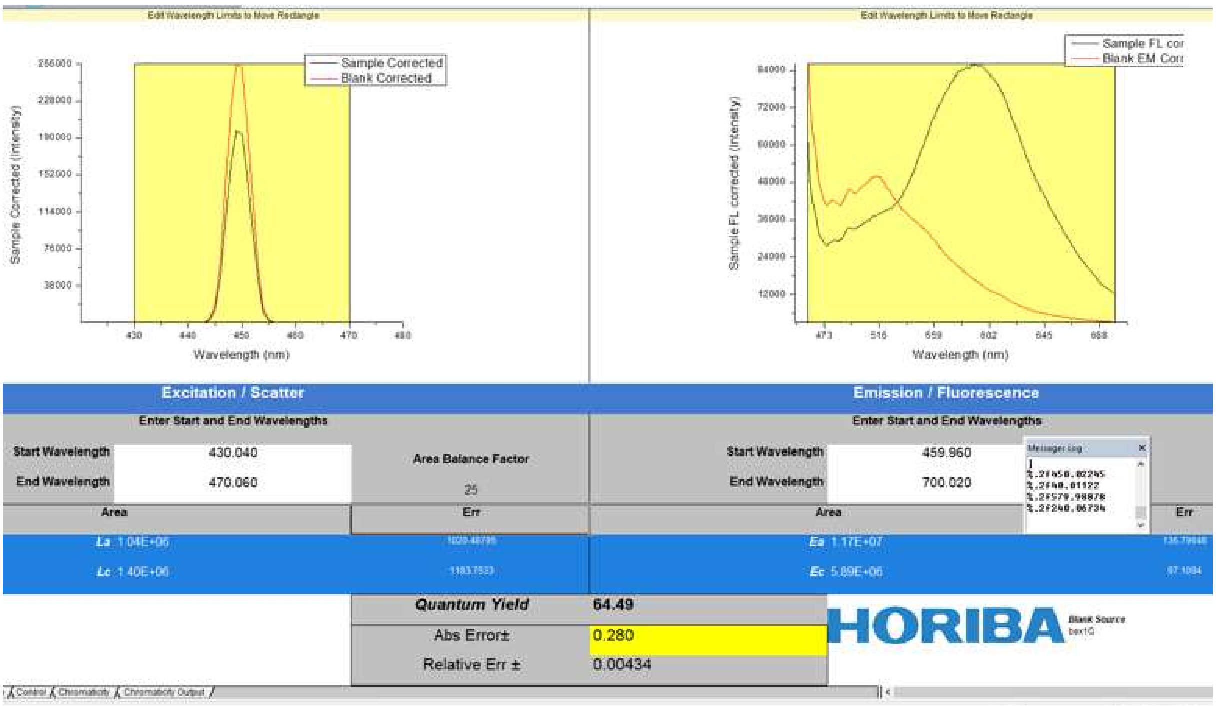


**Supplementary Fig. 37:** PLQY of TAHTCNB co-crystal in solid state measured in the absolute method using integrating sphere.

**Supplementary Table 7**. Time resolved PL Decay of TAHOFN co-crystal Ex. 405 nm and Em. 540 nm.

❖ **Exponential Components Analysis (Reconvolution)**

Fitting range : [112; 510] channels

$\chi^2$ : 0.997

| | $B_i$ | $\Delta B_i$ | $f_i$ (%) | $\Delta f_i$ (%) | $\tau_i$ (ns) | $\Delta\tau_i$ (ns) |
|---|---|---|---|---|---|---|
| 1 | 0.1570 | 0.0018 | 100.000 | 3.524 | 0.505 | 0.012 |

Shift : 0.307 ns (± 0.419 ns)

Decay Background : 1.142 (± 0.139 )

IRF background : 0

**Supplementary Table 8**. Time resolved PL Decay of TAHTFPN_G co-crystal Ex. 405 nm and Em. 545 nm.

❖ **Exponential Components Analysis (Reconvolution)**

Fitting range : [112; 640] channels
$\chi^2$ : 1.009

| | $B_i$ | $\Delta B_i$ | $f_i$ (%) | $\Delta f_i$ (%) | $\tau_i$ (ns) | $\Delta\tau_i$ (ns) |
|---|---|---|---|---|---|---|
| 1 | 0.1347 | 0.0015 | 100.000 | 1.574 | 0.967 | 0.004 |

Shift : 0.236 ns (± 0.643 ns)
Decay Background : 1.096 (± 0.139 )
IRF background : 0

**Supplementary Table 9**. Time resolved PL Decay of TAHTFPN_O co-crystal Ex. 405 nm and Em. 560 nm.

❖ **Exponential Components Analysis (Reconvolution)**

Fitting range : [113; 1500] channels
$\chi^2$ : 1.003

| | $B_i$ | $\Delta B_i$ | $f_i$ (%) | $\Delta f_i$ (%) | $\tau_i$ (ns) | $\Delta\tau_i$ (ns) |
|---|---|---|---|---|---|---|
| 1 | 0.1388 | 0.0022 | 100.000 | 1.876 | 1.064 | 0.003 |

Shift : 0.122 ns (± 0.873 ns)
Decay Background : 1.023 (± 0.062 )
IRF background : 0

**Supplementary Table 10**. Time resolved PL Decay of TAHTCNB co-crystal Ex. 405 nm and Em. 590 nm.

❖ **Exponential Components Analysis (Reconvolution)**

Fitting range : [113; 3900] channels
$\chi^2$ : 1.034

| | $B_i$ | $\Delta B_i$ | $f_i$ (%) | $\Delta f_i$ (%) | $\tau_i$ (ns) | $\Delta\tau_i$ (ns) |
|---|---|---|---|---|---|---|
| 1 | 0.0763 | 0.0052 | 2.970 | 0.858 | 0.570 | 0.126 |
| 2 | 0.0115 | 0.0006 | 4.142 | 0.229 | 5.253 | 0.018 |
| 3 | 0.0433 | 0.0004 | 92.888 | 0.830 | 31.432 | 0.0004 |

Shift : 0.098 ns (± 3.634 ns)
Decay Background : 8.010 (± 1.265 )
IRF background : 0

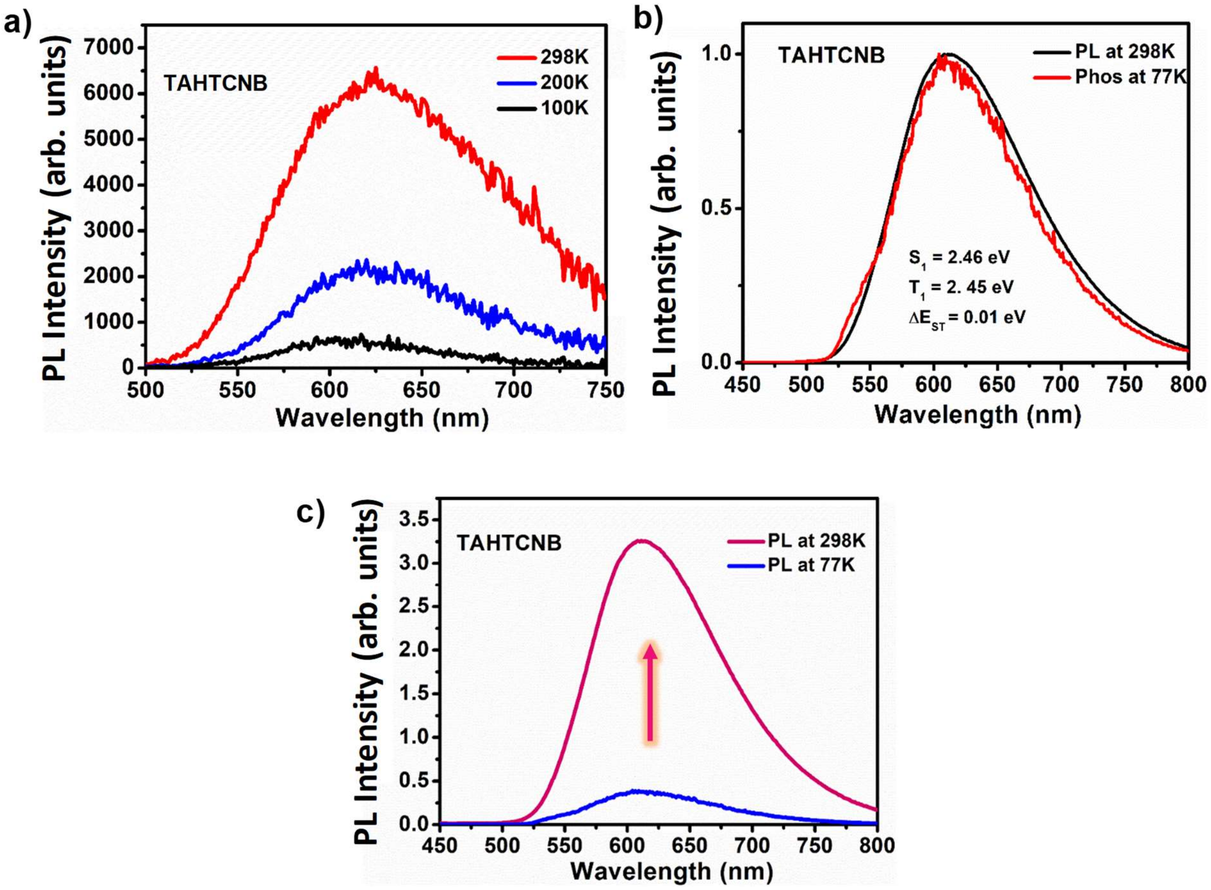


**Supplementary Fig. 38:** a) Temperature dependent delayed emission spectra for TAHTCNB co-crystal in solid state. b) PL at RT and phosphorescence at 77K. c) Steady state PL at RT and 77K for TAHTCNB solid state (powder).

**Supplementary Table 11**. Fitting results for delayed decay at 300K for TAHTCNB co-crystal.

Fit Range (ch) : 185 to 3999

| Parameter | Value | Std. Dev. | Rel % |
|---|---|---|---|
| $\tau_1$ | 8.962E-007 s | 6.0113E-009 s | |
| $\tau_2$ | 9.491E-006 s | 4.8427E-008 s | |
| B1 | 9114.886 | 57.2142 | 47.84 |
| B2 | 938.583 | 7.7630 | 52.16 |
| A | 6.201 | | |
| $\chi^2$ | 1.323 | | |

**Supplementary Table 12**. Fitting results for delayed decay at 200K for TAHTCNB co-crystal.

Fit Range (ch) : 182 to 3999

| Parameter | Value | Std. Dev. | Rel % |
|---|---|---|---|
| $\tau_1$ | 1.096E-006 s | 6.1522E-009 s | |
| $\tau_2$ | 9.409E-006 s | 3.6525E-008 s | |
| B1 | 10989.283 | 55.5040 | 49.77 |
| B2 | 1291.460 | 9.3091 | 50.23 |
| A | 0.537 | | |
| $\chi^2$ | 1.109 | | |

**Supplementary Table 13**. Fitting results for delayed decay at 100K for TAHTCNB co-crystal.

Fit Range (ch) : 182 to 3999

| Parameter | Value | Std. Dev. | Rel % |
|---|---|---|---|
| $\tau_1$ | 1.012E-006 s | 5.5946E-009 s | |
| $\tau_2$ | 9.728E-006 s | 4.3891E-008 s | |
| B1 | 10797.647 | 56.6338 | 53.67 |
| B2 | 969.972 | 7.7391 | 46.33 |
| A | 1.118 | | |
| $\chi^2$ | 1.102 | | |

**Supplementary methods for Electrochemical properties**

To predict the HOMO and LUMO energy levels, the cyclic voltammetry (CV) studies were performed at room temperature by drop casting the luminogens on the working electrodes from THF solution. Supplementary Fig. 39-42 explains the CV curves and the onset values that are obtained and the corresponding energy levels are tabulated in Supplementary Table S14. Both the luminogens displayed only oxidation curves and by substituting the onset values of oxidation into $E_{HOMO}$ = -[($E_{ox}$-$E_{1/2}$ (ferrocene)) + 4.8 eV] the HOMO energy levels were obtained. The LUMO of the monomers were estimated from $E_{HOMO}$ = -[($E_{ox}$-$E_{1/2}$ (ferrocene)) - 4.8 eV].

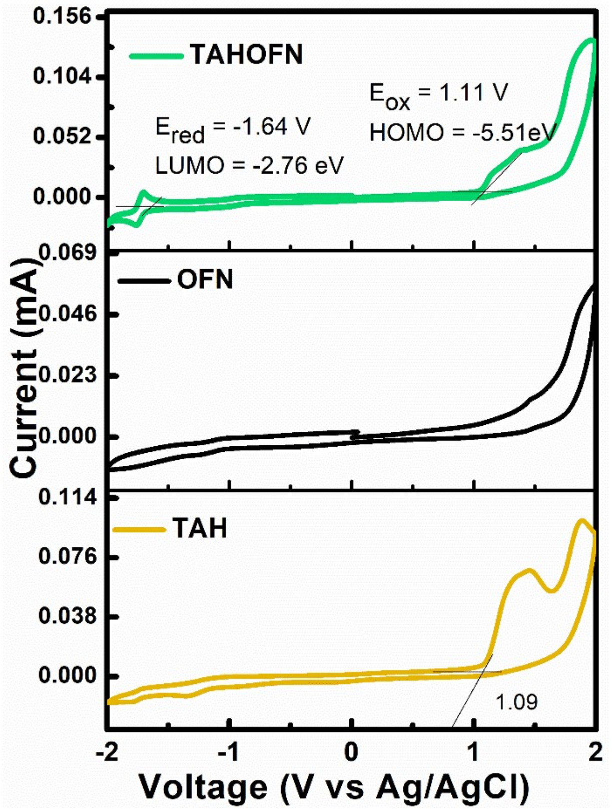


**Supplementary Fig. 39:** Cyclic voltammetry (CV) curves of TAH crystals (yellow line), OFN (black line) and TAHOFN (off-green line).

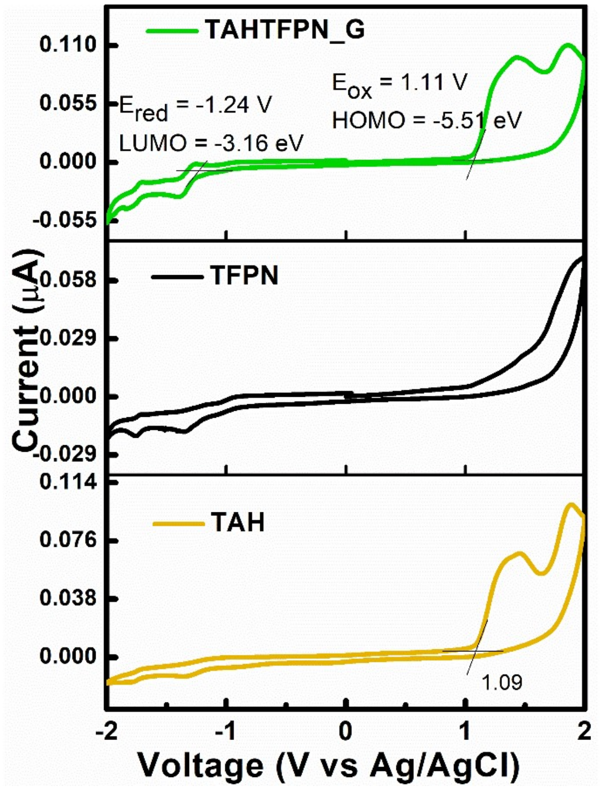


**Supplementary Fig. 40:** Cyclic voltammetry (CV) curves of TAH crystals (yellow line), TFPN (black line) and TAHTFPN_G (green line).

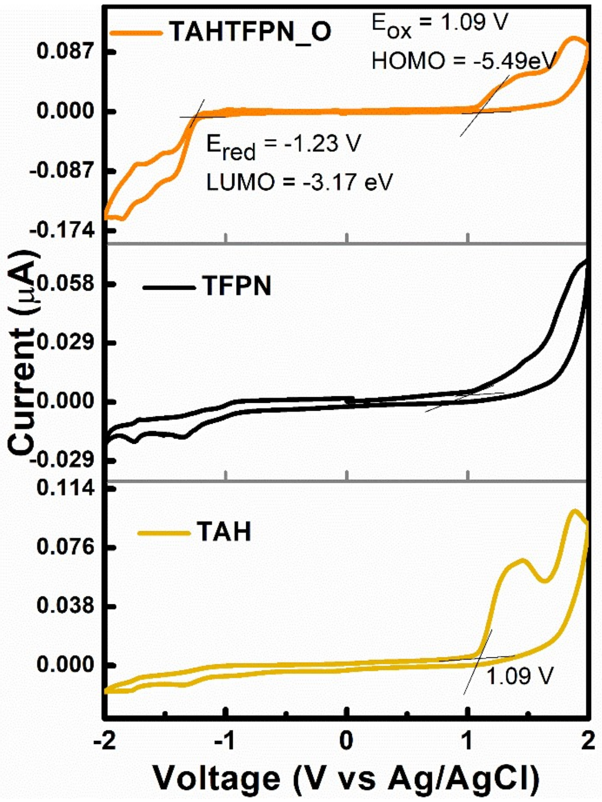


**Supplementary Fig. 41:** Cyclic voltammetry (CV) curves of TAH crystals (yellow line), TFPN (black line) and TAHTFPN_O (orange line).

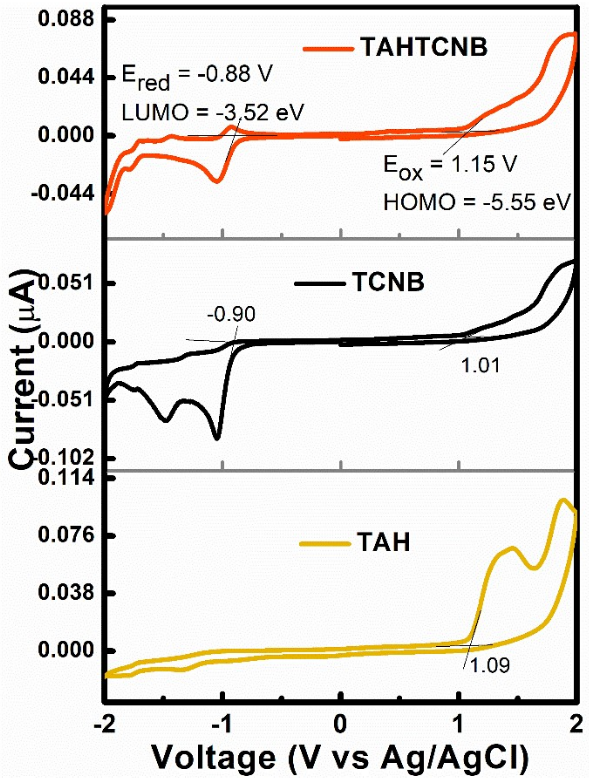


**Supplementary Fig. 42:** Cyclic voltammetry (CV) curves of TAH crystals (yellow line), TCNB (black line) and TAHTCNB (red line).

**Supplementary Table S14.** The energy levels calculated from cyclic voltammetry and DFT.

| Compound | Experimental data | | | | | | DFT data (eV) | | |
|---|---|---|---|---|---|---|---|---|---|
| | $E_{ox}$/V | $E_{red}$/V | $E_{HOMO}$ /eV | $E_{LUMO}$ /eV | $E_{opt}$ /eV | H-L Gap eV | HOMO | LUMO | H-L Gap |
| **TAH** | 1.09 | - | -5.49 | -2.96 | 2.53 | 2.53 | -5.47 | -2.17 | 3.29 |
| **TAHOFN** | 1.11 | -1.64 | -5.51 | -2.76 | - | 2.75 | -5.51 | -2.76 | 2.75 |
| **TAHTFPN_G** | 1.11 | -1.24 | -5.51 | -3.16 | - | 2.35 | -5.58 | -2.69 | 2.89 |
| **TAHTFPN_O** | 1.09 | -1.23 | -5.49 | -3.17 | - | 2.32 | -5.48 | -2.80 | 2.68 |
| **TAHTCNB** | 1.15 | -0.88 | -5.55 | -3.52 | - | 2.03 | -5.55 | -3.52 | 2.033 |

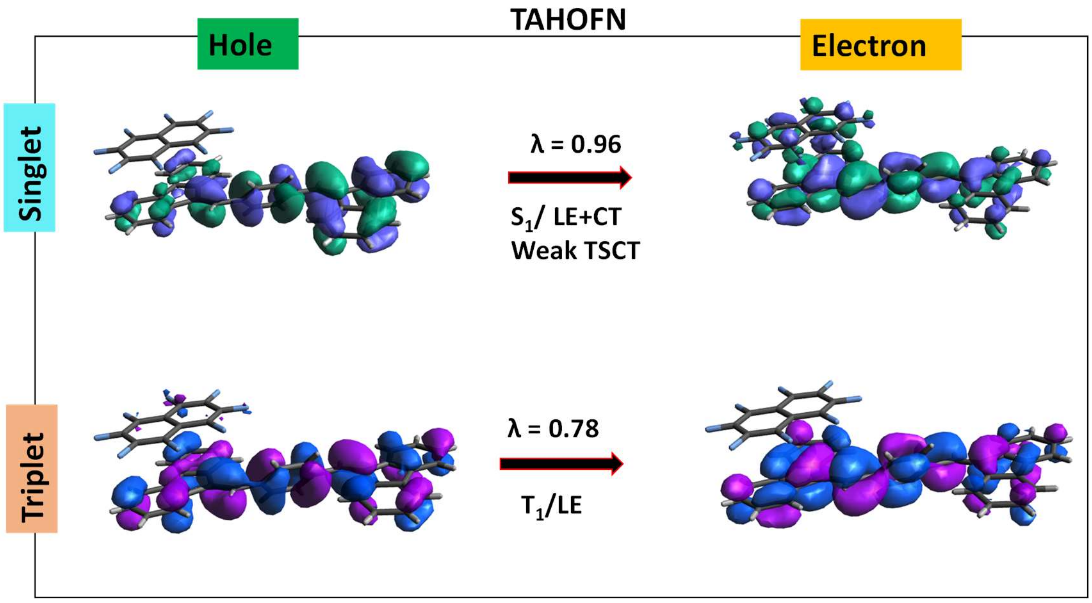


**Supplementary Fig. 43:** Natural transition orbitals (NTOs)[4] for lowest excited singlet and triplet excited states in TAHOFN.

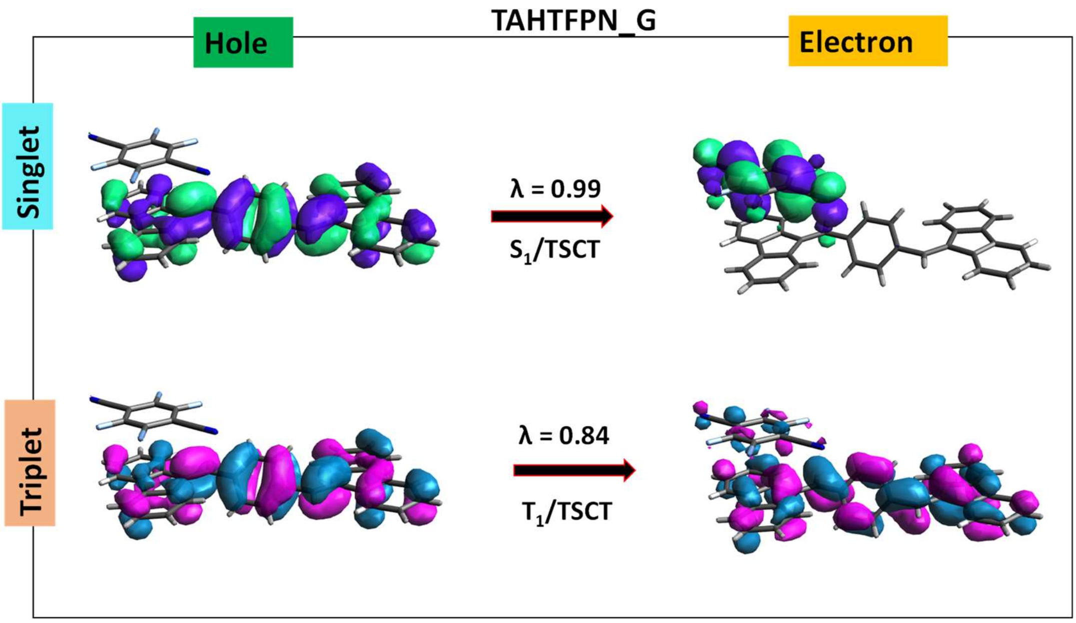


**Supplementary Fig. 44:** Natural transition orbitals (NTOs)[4] for lowest excited singlet and triplet excited states in TAHTFPN_G.

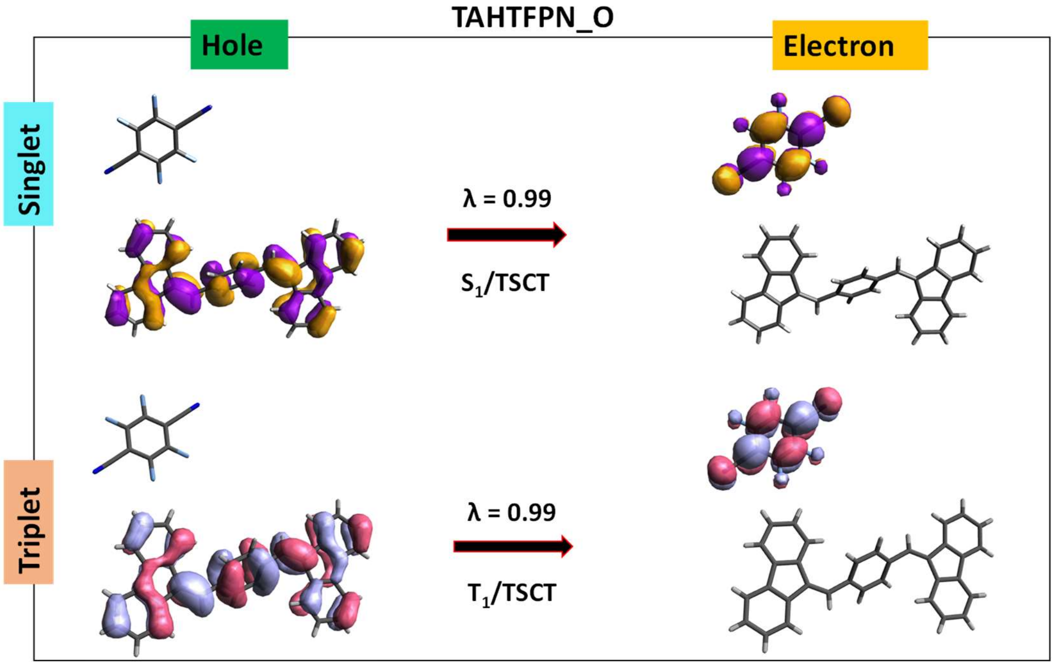


**Supplementary Fig. 45:** Natural transition orbitals (NTOs)[4] for lowest excited singlet and triplet excited states in TAHTFPN_O.

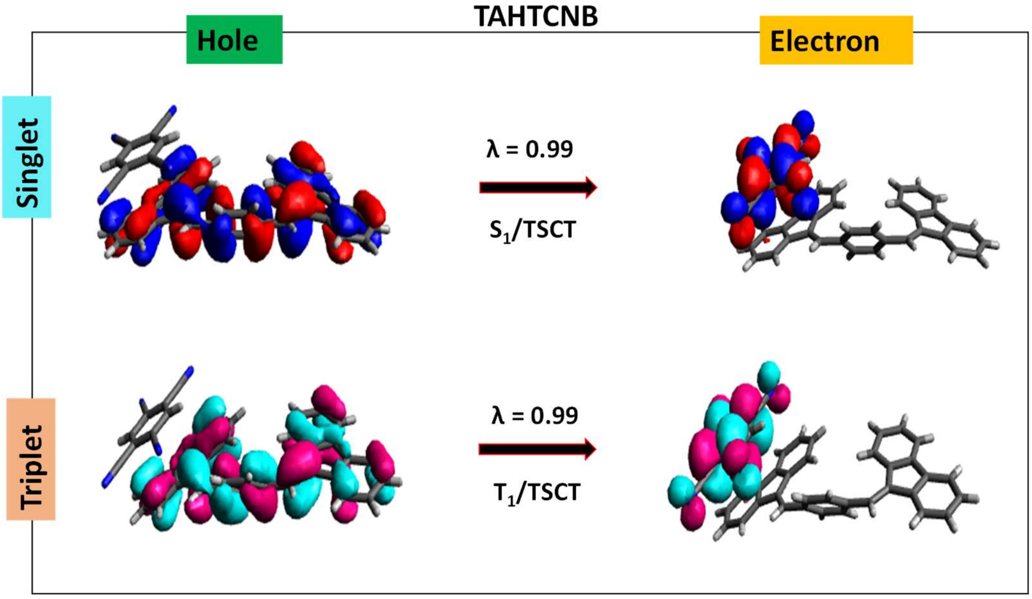


**Supplementary Fig. 46:** Natural transition orbitals (NTOs)[4] for lowest excited singlet and triplet excited states in TAHTCNB.

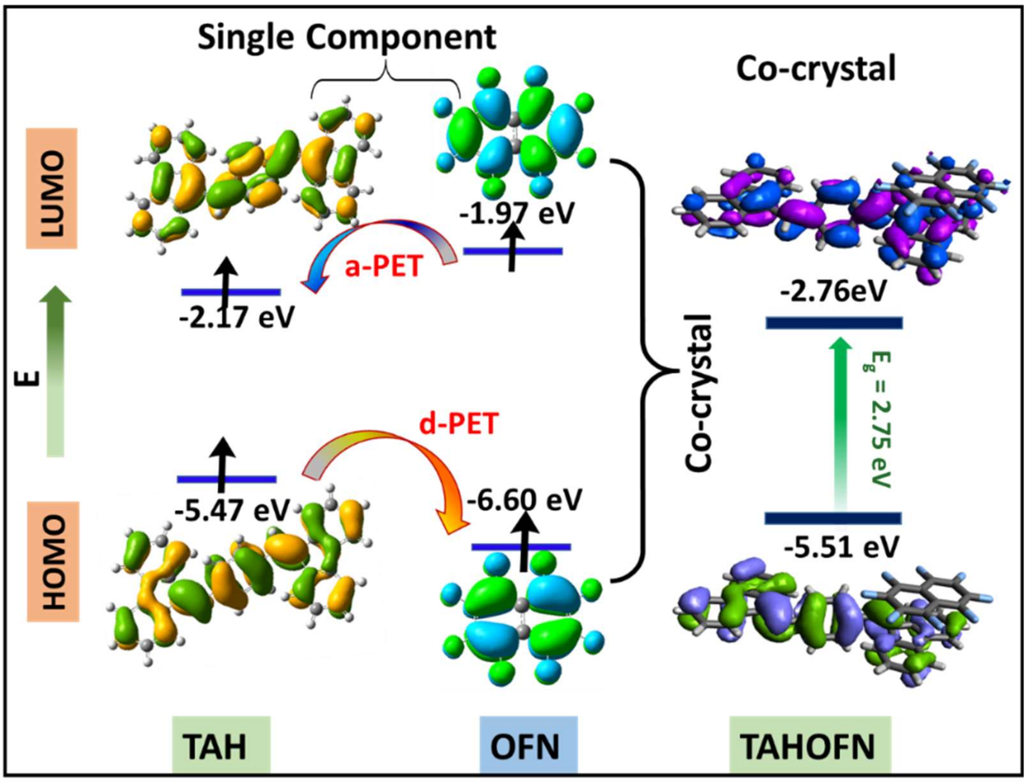


**Supplementary Fig. 47:** Mechanism of weak fluorescence (a-PET & d-PET) for TAHOFN by DFT/TD-DFT[4] calculated HOMO/LUMO energy band diagram for conformer TAH and OFN.

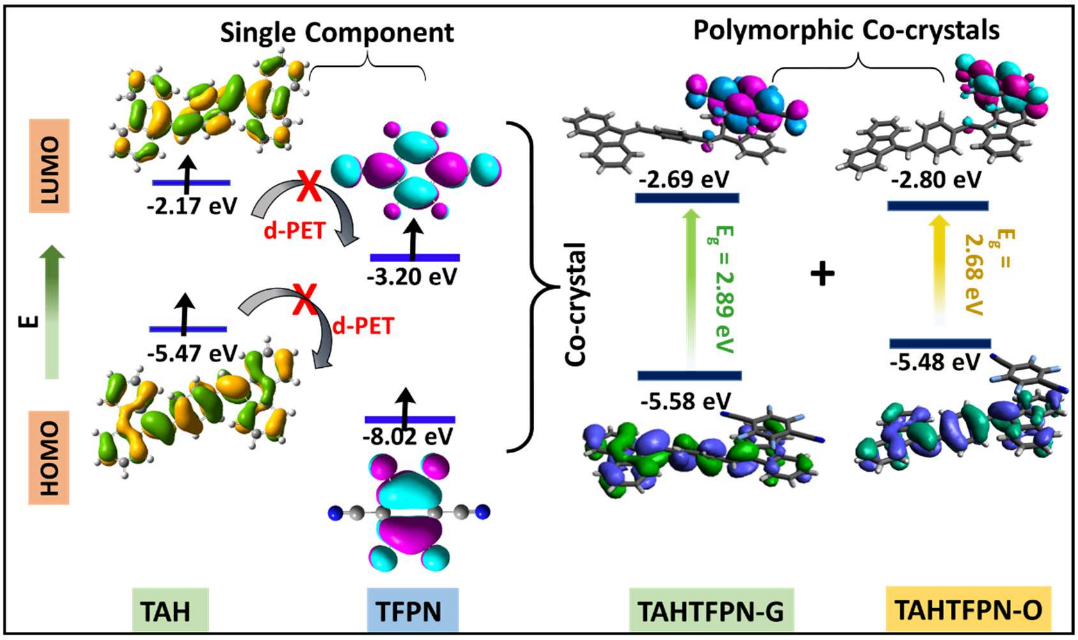


**Supplementary Fig. 48:** Color-tunable fluorescence and CT mechanism for the polymorphic co-crystals of TAHTFPN_G and TAHTFPN_O by DFT/TD-DFT[4] calculated HOMO/LUMO energy band diagram for the conformer TAH and TFPN.

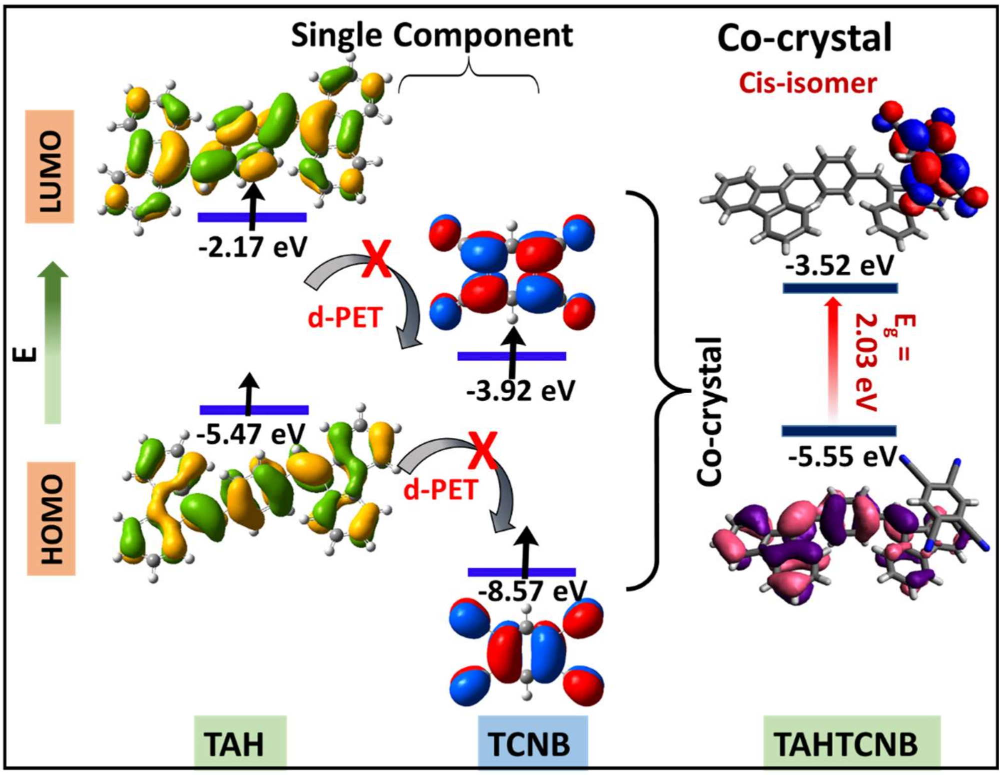


**Supplementary Fig. 49:** Red fluorescence and strong narrow band CT mechanism for the cis-isomeric co-crystals of TAHTCNB by DFT/TD-DFT[4] calculated HOMO/LUMO energy band diagram for the conformer TAH and TCNB.

**Supplementary Table 15.** Vertical energy transitions with major frontier orbital contribution for TAHOFN cocrystal at B3LYP/6-31G(d,p) level for singlet states[4].

| State | Energy (eV) | Energy ($cm^{-1}$) | Wavelength (nm) | Osc. Strength (*f*) | Symmetry | Major contributions |
|---|---|---|---|---|---|---|
| $S_1$ | 3.19 | 25741.18 | 388 | 0.5471 | H-1→LUMO | 22% |
| $S_2$ | 3.21 | 25943.63 | 385 | 0.3236 | H-1→LUMO | 64% |
| $S_3$ | 3.30 | 26652.59 | 375 | 0.0058 | H-2→LUMO | 93% |
| $S_4$ | 3.59 | 29025.47 | 344 | 0.0674 | H-3→LUMO | 23% |
| $S_5$ | 3.61 | 29162.59 | 342 | 0.0344 | H-4→LUMO | 14% |
| $S_6$ | 3.71 | 29990.92 | 333 | 0.0564 | H-4→LUMO | 52% |
| $S_7$ | 3.75 | 30253.05 | 330 | 0.0012 | H-2→L+1 | 86% |
| $S_8$ | 3.76 | 30356.29 | 329 | 0.0054 | H-2→L+1 | 13% |
| $S_9$ | 3.99 | 32217.01 | 310 | 0.0003 | H-1→L+1 | 35% |
| $S_{10}$ | 4.03 | 32531.57 | 307 | 0.001 | H-4→LUMO | 17% |

**Supplementary Table 16.** Vertical energy transitions with major frontier orbital contribution for TAHOFN cocrystal at B3LYP/6-31G (d,p) level for triplet states[4].

| State | Energy (eV) | Energy ($cm^{-1}$) | Wavelength (nm) | Osc. Strength (*f*) | Symmetry | Major contributions |
|---|---|---|---|---|---|---|
| T1 | 2.23 | 18025.68 | 554 | 0 | HOMO→LUMO | 72% |
| T2 | 2.47 | 19940.45 | 501 | 0 | H-4→LUMO | 16% |
| T3 | 2.65 | 21430.96 | 466 | 0 | H-1→L+1 | 33% |
| T4 | 2.72 | 21985.06 | 454 | 0 | H-1→LUMO | 44% |
| T5 | 2.77 | 22404.47 | 446 | 0 | H-2→LUMO | 62% |
| T6 | 3.43 | 27675.3 | 361 | 0 | H-8→LUMO | 13% |

| | | | | | | |
|---|---|---|---|---|---|---|
| **T7** | 3.46 | 27953.56 | 357 | 0 | H-1→LUMO | 37% |
| **T8** | 3.49 | 28214.89 | 354 | 0 | H-2→LUMO | 24% |
| **T9** | 3.57 | 28827.06 | 346 | 0 | HOMO→L+1 | 58% |
| **T10** | 3.62 | 29209.37 | 342 | 0 | H-4→LUMO | 37% |

**Supplementary Table 17.** Vertical energy transitions with major frontier orbital contribution for TAHTFPN_G cocrystal at B3LYP/6-31G(d,p) level for singlet states[4].

| State | Energy (eV) | Energy ($cm^{-1}$) | Wavelength (nm) | Osc. Strength (*f*) | Symmetry | Major contributions |
|---|---|---|---|---|---|---|
| $S_1$ | 2.45 | 19812.20 | 504 | 0.061 | HOMO→LUMO | 96% |
| $S_2$ | 2.76 | 22304.45 | 448 | 0.0043 | H-2→LUMO | 99% |
| $S_3$ | 2.82 | 22811.77 | 438 | 0.0001 | H-1→LUMO | 98% |
| $S_4$ | 3.07 | 24777.35 | 403 | 0.8762 | HOMO→L+1 | 91% |
| $S_5$ | 3.10 | 25037.06 | 399 | 0.0585 | H-3→LUMO | 88% |
| $S_6$ | 3.14 | 25351.61 | 394 | 0.0735 | H-1→L+1 | 87% |
| $S_7$ | 3.32 | 26823.58 | 372 | 0.0051 | H-2→L+1 | 95% |
| $S_8$ | 3.67 | 29618.29 | 337 | 0.1259 | H-3→L+1 | 53% |
| $S_9$ | 3.69 | 29770.73 | 335 | 0.0049 | H-5→LUMO | 96% |
| $S_{10}$ | 3.78 | 30561.15 | 327 | 0.0009 | H-4→LUMO | 98% |

**Supplementary Table 18.** Vertical energy transitions with major frontier orbital contribution for TAHTFPN_G cocrystal at B3LYP/6-31G (d,p) level for triplet states[4].

| State | Energy (eV) | Energy ($cm^{-1}$) | Wavelength (nm) | Osc. Strength (*f*) | Symmetry | Major contributions |
|---|---|---|---|---|---|---|
| **T1** | 2.09 | 16873.92 | 592 | 0 | HOMO→LUMO | 13% |
| **T2** | 2.42 | 19562.97 | 511 | 0 | HOMO→LUMO | 57% |
| **T3** | 2.51 | 20267.09 | 493 | 0 | H-3→L+1 | 38% |
| **T4** | 2.68 | 21689.86 | 461 | 0 | H-1→LUMO | 17% |
| **T5** | 2.71 | 21862.46 | 457 | 0 | H-2→LUMO | 74% |
| **T6** | 2.83 | 22872.27 | 437 | 0 | H-2→LUMO | 25% |
| **T7** | 2.86 | 23068.26 | 433 | 0 | H-1→LUMO | 82% |
| **T8** | 3.08 | 24855.58 | 402 | 0 | H-3→LUMO | 86% |
| **T9** | 3.32 | 26809.86 | 372 | 0 | H-11→LUMO | 46% |
| **T10** | 3.37 | 27222.01 | 367 | 0 | H-11→LUMO | 12% |

**Supplementary Table 19.** Vertical energy transitions with major frontier orbital contribution for TAHTFPN_O cocrystal at B3LYP/6-31G(d,p) level for singlet states[4].

| State | Energy (eV) | Energy ($cm^{-1}$) | Wavelength (nm) | Osc. Strength (*f*) | Symmetry | Major contributions |
|---|---|---|---|---|---|---|
| **$S_1$** | 2.26 | 18270.07 | 547 | 0.0445 | HOMO→LUMO | 96% |
| **$S_2$** | 2.57 | 20767.16 | 481 | 0.0157 | H-2→LUMO | 98% |
| **$S_3$** | 2.68 | 21673.73 | 461 | 0.0164 | H-1→LUMO | 84% |
| **$S_4$** | 2.78 | 22429.47 | 445 | 0.9245 | HOMO→L+1 | 82% |
| **$S_5$** | 2.84 | 22967.44 | 435 | 0.2535 | H-1→L+1 | 75% |
| **$S_6$** | 2.97 | 23964.35 | 417 | 0.0055 | H-2→L+1 | 96% |
| **$S_7$** | 2.98 | 24103.88 | 414 | 0.0126 | H-3→LUMO | 94% |
| **$S_8$** | 3.37 | 27228.47 | 367 | 0.0558 | H-3→L+1 | 58% |

| | | | | | | |
|---|---|---|---|---|---|---|
| $S_9$ | 3.66 | 29580.38 | 338 | 0.0021 | H-1→L+2 | 81% |
| $S_{10}$ | 3.67 | 29649.75 | 337 | 0.0045 | H-5→LUMO | 91% |

**Supplementary Table 20.** Vertical energy transitions with major frontier orbital contribution for TAHTFPN_O cocrystal at B3LYP/6-31G (d,p) level for triplet states[4].

| State | Energy (eV) | Energy ($cm^{-1}$) | Wavelength (nm) | Osc. Strength (*f*) | Symmetry | Major contributions |
|---|---|---|---|---|---|---|
| **T1** | 1.72 | 13934.84 | 717 | 0 | HOMO→L+1 | 77% |
| **T2** | 2.17 | 17575.63 | 568 | 0 | H-3→L+1 | 35% |
| **T3** | 2.28 | 18464.45 | 541 | 0 | H-3→L+1 | 12% |
| **T4** | 2.42 | 19558.94 | 511 | 0 | H-1→L+1 | 60% |
| **T5** | 2.47 | 19937.22 | 501 | 0 | H-2→LUMO | 57% |
| **T6** | 2.57 | 20778.46 | 481 | 0 | H-2→LUMO | 40% |
| **T7** | 2.72 | 22003.61 | 454 | 0 | H-1→LUMO | 91% |
| **T8** | 2.97 | 23986.12 | 416 | 0 | H-3→LUMO | 88% |
| **T9** | 3.24 | 26138.82 | 382 | 0 | H-3→L+1 | 27% |
| **T10** | 3.27 | 26436.43 | 378 | 0 | H-11→LUMO | 54% |

**Supplementary Table 21.** Vertical energy transitions with major frontier orbital contribution for TAHTCNB cocrystal at B3LYP/6-31G(d,p) level for singlet states[4].

| State | Energy (eV) | Energy ($cm^{-1}$) | Wavelength (nm) | Osc. Strength (*f*) | Symmetry | Major contributions |
|---|---|---|---|---|---|---|
| $S_1$ | 1.95 | 15740.72 | 635 | 0.0047 | HOMO→LUMO | 93% |
| $S_2$ | 2.19 | 17703.87 | 564 | 0.0174 | H-2→LUMO | 80% |
| $S_3$ | 2.20 | 17759.52 | 563 | 0.0049 | H-2→LUMO | 19% |

| | | | | | | |
|---|---|---|---|---|---|---|
| **S4** | 2.47 | 19933.99 | 501 | 0.0078 | H-3→LUMO | 92% |
| **S5** | 2.62 | 21180.93 | 472 | 0.0293 | HOMO→L+1 | 92% |
| **S6** | 2.83 | 22868.24 | 437 | 0.0005 | H-2→L+1 | 99% |
| **S7** | 2.88 | 23253.77 | 430 | 0.00 | H-1→L+1 | 98% |
| **S8** | 3.10 | 25066.1 | 398 | 0.0012 | H-6→LUMO | 25% |
| **S9** | 3.14 | 25360.49 | 394 | 0.0289 | H-3→L+1 | 84% |
| **S10** | 3.17 | 25627.46 | 390 | 0.0005 | H-6→LUMO | 13% |

**Supplementary Table 22.** Vertical energy transitions with major frontier orbital contribution for TAHTCNB cocrystal at B3LYP/6-31G (d,p) level for triplet states[4].

| State | Energy (eV) | Energy ($cm^{-1}$) | Wavelength (nm) | Osc. Strength (*f*) | Symmetry | Major contributions |
|---|---|---|---|---|---|---|
| **T1** | 1.93 | 15599.57 | 641 | 0 | HOMO→LUMO | 91% |
| **T2** | 2.13 | 17233.65 | 580 | 0 | H-2→LUMO | 99% |
| **T3** | 2.20 | 17748.23 | 563 | 0 | H-1→LUMO | 98% |
| **T4** | 2.23 | 18033.75 | 554 | 0 | H-3→L+3 | 10% |
| **T5** | 2.45 | 19787.2 | 505 | 0 | H-3→LUMO | 89% |
| **T6** | 2.48 | 20079.17 | 498 | 0 | H-3→L+2 | 35% |
| **T7** | 2.64 | 21342.24 | 468 | 0 | H-3→L+2 | 10% |
| **T8** | 2.73 | 22033.45 | 453 | 0 | H-1→L+2 | 38% |
| **T9** | 2.74 | 22157.66 | 451 | 0 | H-2→L+1 | 56% |
| **T10** | 2.89 | 23311.04 | 428 | 0 | H-10→LUMO | 12% |

**Supplementary Table 23.** SOC-matrix element (SOCME) calculated by using ORCA 4.5.0 software with ORCA_SOC module[5].

| | SOCME_TAHOFN ($cm^{-1}$) | | SOCME_TAHTFPN-G ($cm^{-1}$) | | SOCME_TAHOFN-O ($cm^{-1}$) | | SOCME_TAHTCNB ($cm^{-1}$) | |
|---|---|---|---|---|---|---|---|---|
| State | $\langle S_0\|H_{SO}\|T_m\rangle^a$ | $\langle S_1\|H_{SO}\|T_m\rangle^a$ | $\langle S_0\|H_{SO}\|T_m\rangle^a$ | $\langle S_1\|H_{SO}\|T_m\rangle^a$ | $\langle S_0\|H_{SO}\|T_m\rangle^a$ | $\langle S_1\|H_{SO}\|T_m\rangle^a$ | $\langle S_0\|H_{SO}\|T_m\rangle^a$ | $\langle S_1\|H_{SO}\|T_m\rangle^a$ |
| T1 | 0.0424 | 0.036 | 0.092 | 0.269 | 0.045 | 0.125 | 0.162 | **0.01** |
| T2 | 0.902 | 0.051 | 0.06 | 0.150 | 0.420 | 0.021 | 0.446 | **0.04** |
| T3 | 0.189 | 0.080 | 0.739 | 0.276 | 0.837 | 0.112 | 0.014 | **0.06** |

$^a\sqrt{\Sigma\langle S_n|H_{SO}|T_m\ (MS=0,\pm1)\rangle^2}$

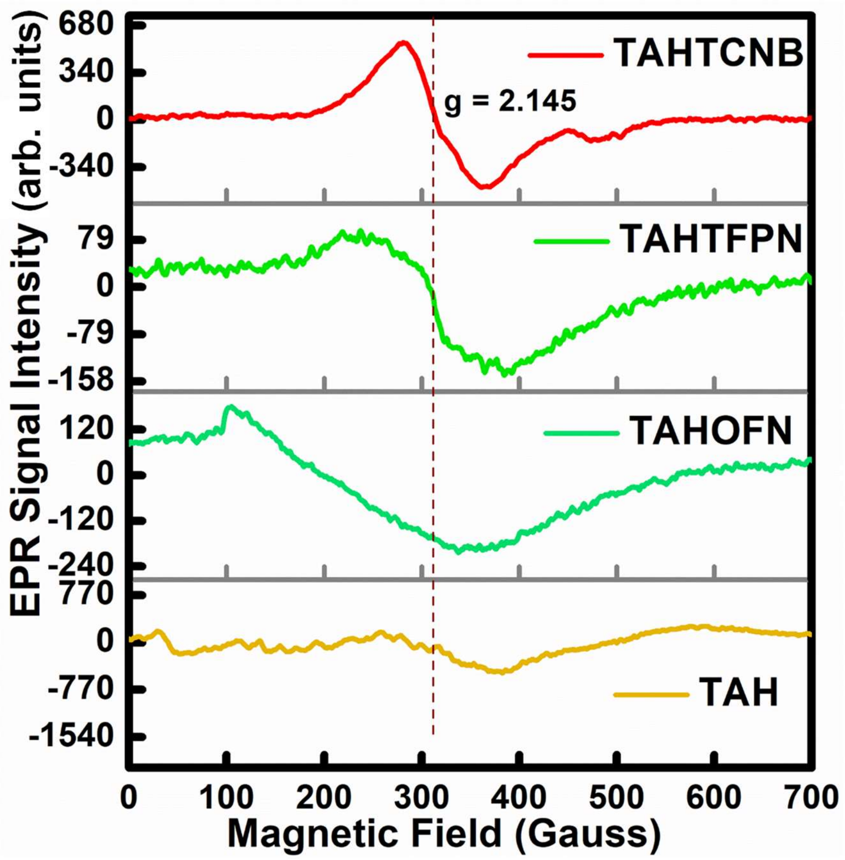


**Supplementary Fig. 50:** Electron spin resonance (ESR) spectra for TAH (yellow line), TAHOFN (off green line), TAHTFPN_G (green line) and TAHTCNB (red line).

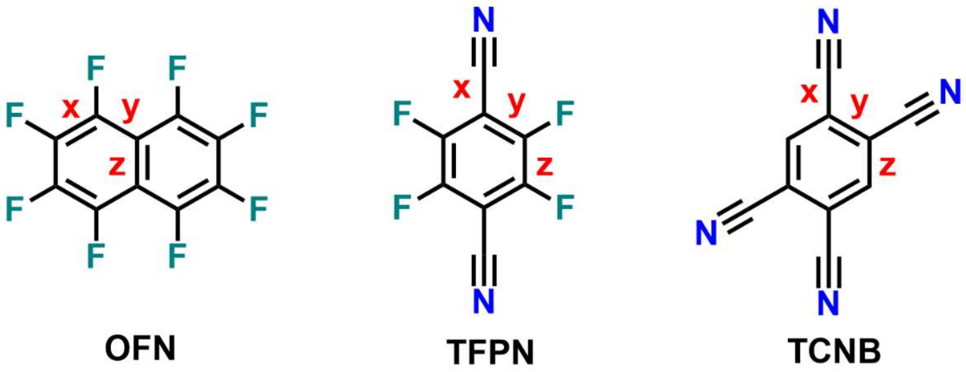


**Supplementary Fig. 51:** Chemical structures of OFN, TFPN and TCNB molecule, where, x, y, and z are the bond length of carbon-carbon bond at neutral state.

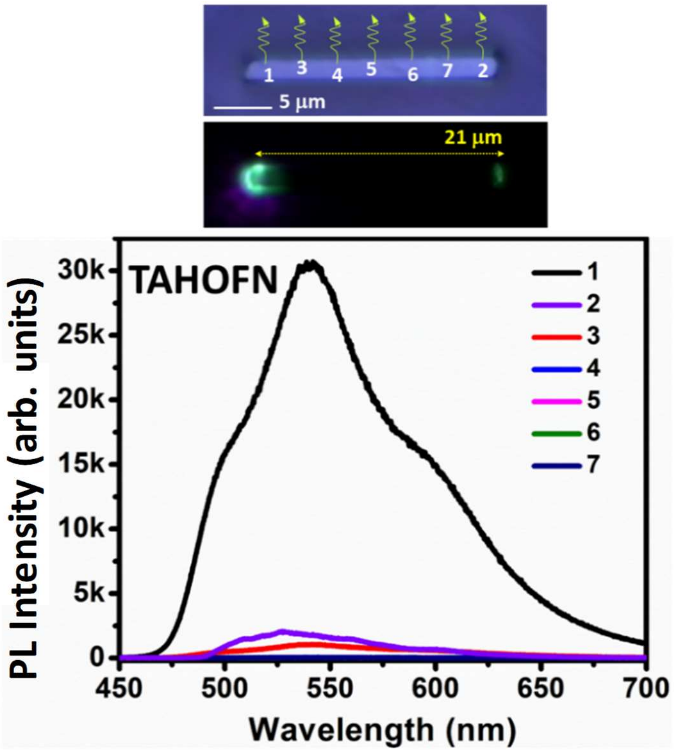


**Supplementary Fig. 52:** Spatially resolved spectra of TAHOFN crystal: Excitation position was kept constant and PL signals were collected at different positions**.**

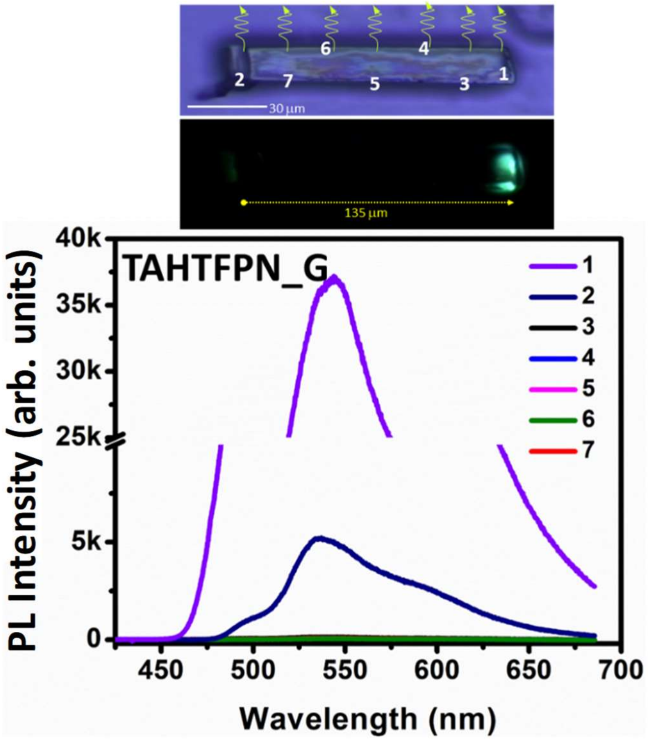


**Supplementary Fig. 53:** Spatially resolved spectra of TAHTFPN_G crystal: Excitation position was kept constant and PL signals were collected at different positions.

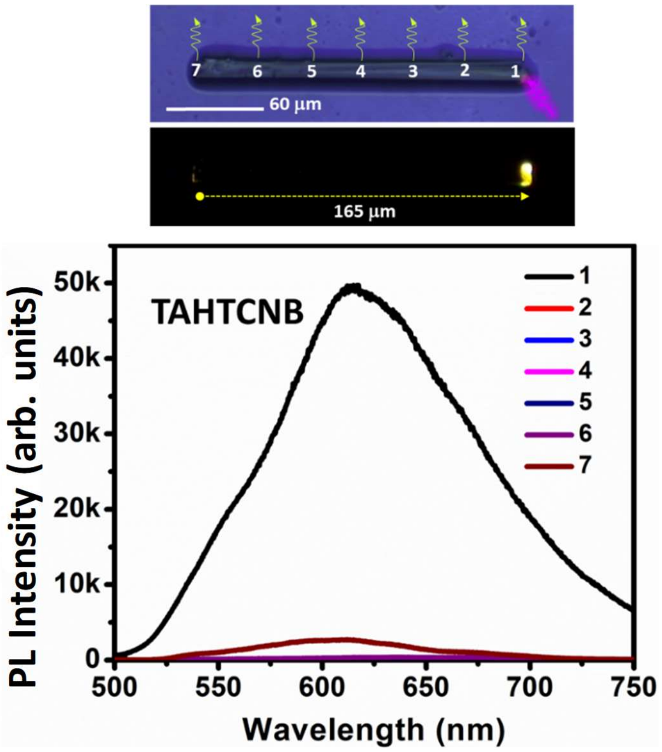


**Supplementary Fig. 54:** Spatially resolved spectra of TAHTCNB crystal: Excitation position was kept constant and PL signals were collected at different positions.

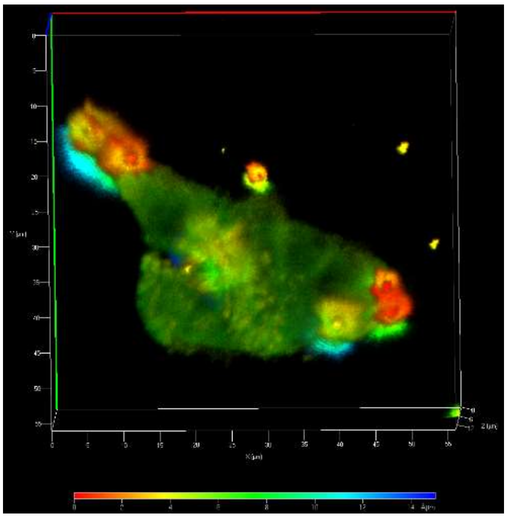

**Supplementary Fig. 55:** Z-stacking analysis of the MCF-7, confirming the internalization and localization of the TAHOFN in the cytoplasm. (Scale bar: 5 μm).

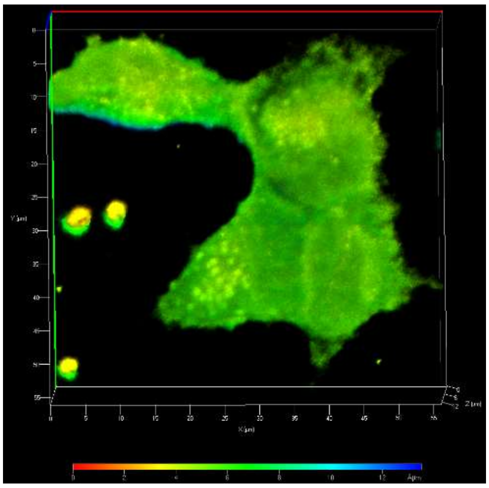

**Supplementary Fig. 56:** Z-stacking analysis of the MCF-7, confirming the internalization and localization of the TAHTFPN_G in the cytoplasm. (Scale bar: 5 μm).

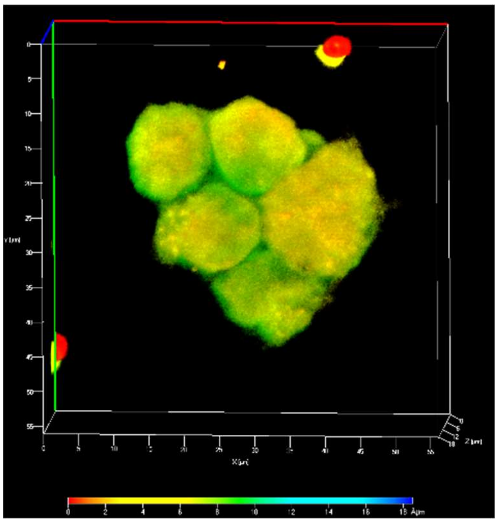

**Supplementary Fig. 57:** Z-stacking analysis of the MCF-7, confirming the internalization and localization of the TAHTCNB in the cytoplasm. (Scale bar: 5 μm).

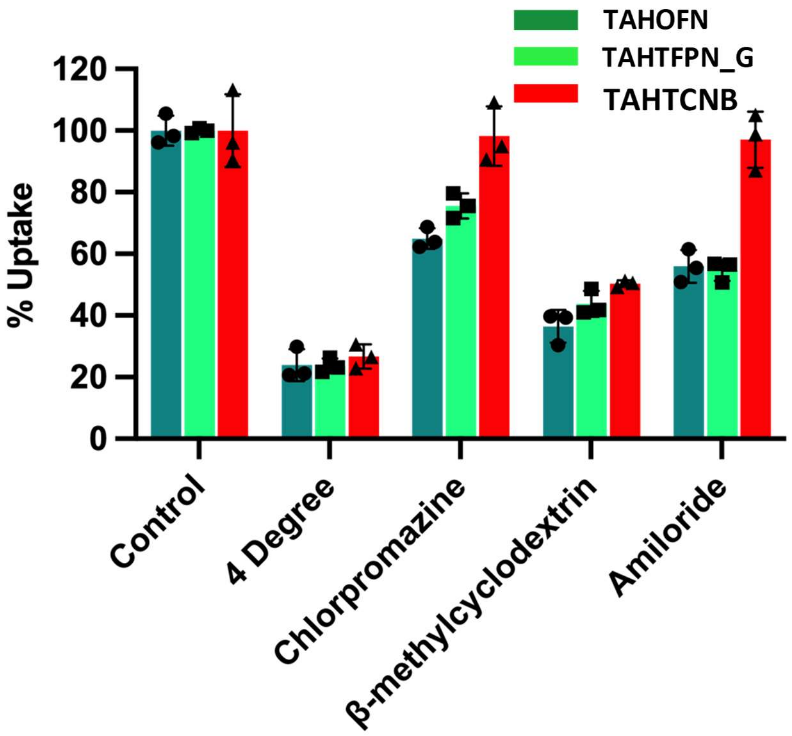


**Supplementary Fig. 58:** Celluler internalization mechanism studies using co-crystals. Data are shown as the mean ± SD (n=3). The experimental results shown in this figure was independently replicated three times, demonstrating consistent outcomes.

**Supplementary Table 24:** A brief summary of the previous report on various optically waveguide active molecular crystals.

| Name | Emission peak / nm | Fluorescent lifetime / ns | Delayed Fluorescent lifetime / µs | PLQY | Loss co-efficient | Reference |
|---|---|---|---|---|---|---|
| 2-acetyl-6dimethylaminonaphthalene (ADN) | 450 | - | - | - | 0.069 dB/µm | *Angew. Chem.*, **2013**, *125*, 8875-8879 |
| 2-acetyl-6-methylaminonaphthalene (AMN) | 540 | - | - | - | 0.076 dB/µm and 0.457 dB/µm along different directions | *Angew. Chem.*, **2013**, *125*, 8875-8879 |
| 2,2′-((5,5′-(3,7-dicyano-2,6 bis(dihexylamino) benzo[1,2 b:4,5-b′]difuran 4,8- diyl)bis(thiophe-ne-5,2-diyl)) bis(methanylylidene))dimalononitrile (BDFTM) | 676 | 2.02 | - | 16% | 0.06 dB/µm | *Adv. Funct. Mater.* **2014**, *24*, 4250-4258 |
| One-dimensional (1D) ribbon-like perylene crystals | 580 | - | - | 5% | 0.072 dB/µm | *J. Mater. Chem. C,* **2014**, *2*, 9695-9700 |
| Two-dimensional (2D) square-like perylene crystals | 580 | - | - | 5% | 0.099 and 0.101 dB/µm along different directions | *J. Mater. Chem. C,* **2014**, *2*, 9695-9700 |
| BDTVA (a 9,10-distyrylanthracene derivative) | 509 | 1.96 | - | 50% | 0.000275 dB/µm | *ACS Photonics*, **2015**, *2*, 313-318 |
| The cocrystal of trans-1,2 bis(4pyridyl)ethylene and 1,2,4,5tetracyanobenzene | 436 | 6.13 | - | 19% | 0.26 dB/µm (isotropic 2D waveguide) | *Angew. Chem. Int. Ed.,* **2015**, *54*, 6785 6789 |
| The cocrystal of 1,2-di(4pyridyl)ethylene and 1,3,5-trifluoro2,4,6-triiodobenzene | 440 | 1.66 | - | 26.1% | 0.19 dB/µm | *J. Am. Chem. Soc.,* **2015**, *137*, 1103811046 |

| | | | | | | |
|---|---|---|---|---|---|---|
| A solvate composed of a carbazole and cyano-substituted tetraphenylethylene derivative cocrystalized with DMF | 458 | 2.45 | - | 41% | 0.00804 dB/μm | *Small*, **2016**, *47*, 6554-6561 |
| A solvate composed of a carbazole and cyano-substituted tetraphenylethylene derivative cocrystalized with $CHCl_3$ | 481 | 1.93 | - | 65% | 0.00708 dB/μm | *Small*, **2016**, *47*, 6554-6561 |
| A solvate composed of a carbazole and cyano-substituted tetraphenylethylene derivative cocrystalized with $CH_2Cl_2$ | 522 | 3.01 | - | 40% | 0.00301 dB/μm | *Small*, **2016**, *47*, 6554-6561 |
| The cocrystal of 4-(1-naphthylvinyl) pyridine and 1,2,4,5tetracyanobenzene | about 550 | 7.5 | - | 6.8% | 0.040-0.11 dB/μm | *Adv. Mater.,* **2016**, *28*, 5954-5962 |
| PyB (composed of two chromophores of a pyrene unit and a rhodamine B moiety) | 492 | 6.73 | - | 3.52% | 0.00669 dB/μm at 440 nm and 0.00351 dB/μm at 497 nm | *ACS Appl. Mater. Interfaces,* **2017**, *9*, 8910-8918 |
| BCZ (a carbazole-containing βdiketonate derivative) | 567 | - | - | 37% | 0.033 dB/μm | *Adv. Funct. Mater.,* **2017**, *27*, 1700332 |
| 2,5-dihydro-3,6-bis(octylamino) terephthalate doped with 3,6-bis(octylamino) terephthalate | 572 | 12.7 | - | 56% | 0.000272 dB/μm at 576 nm and 0.000196 dB/μm at 615 nm | *Adv. Mater.,* **2018**, *30*, 1800814 |
| The cocrystal of 4,4’-((1*E*,1’*E* (2,5dimethoxy-1,4-phenylene)bis(ethene 2,1-diyl))dipyridine and 1,4diiodotetrafluorobenzene | 525 | - | - | - | 0.0145 dB/μm at 525 nm for microtubes; 0.0341 dB/μm for microrods | *J. Mater. Chem. C,* **2018**, *6*, 9594-9598 |
| 7-(diethylamino)coumarin-3- aldehyde | 564 | 8.25 | - | 7.5% | 0.0058 dB/μm | *Mater. Chem. Front.,* **2018**, *2,* 910-916 |
| (*E*)-1-(4-(dimethylamino) phenyl)iminomethyl-2-hydroxyl-naphthalene | 615 | - | - | 43% | 0.00027 dB/μm for the straight, | *Angew. Chem. Int. Ed.,* **2018**, *57*, 8448 - 8452 |

| | | | | | | |
|---|---|---|---|---|---|---|
| | | | | | 0.000274 dB/μm for the bent state | |
| dimethyl 2,5-bis((2hydroxyethyl)amino)terephthalate | 641 | 6.1 | - | 8% | 0.000351 dB/μm for the straight state; 0.000376 dB/μm for the bent state | *J. Phys. Chem. Lett.*, **2019**, *10*, 1437-1442 |
| 2,5-dimethoxybenzene-1,4dicarboaldehyde | 499 | 4.0 | - | 42% | 0.00120 dB/μm | *ChemPlusChem*, **2019**, *84*, 247-251 |
| 1,4-trimethylsilylethynyl anthracene (TMS ANT) | 538 | | - | - | about 0.002 dB/μm | *Chem. Mater.* **2019**, *31*, 1775-1783 |
| 1,2,3,4-Tetrafluoro-5,8bis(trimethylsilylethynyl)anthracene (F4 TMS ANT) | 559 | - | - | - | about 0.0013 dB/μm | *Chem. Mater.* **2019**, *31*, 1775-1783 |
| The 1D cocrystal of fluoranthene and 1,2,4,5-tetracyanobenzene | about 550 | 80 | - | 74% | 0.0181 dB/μm along [100] direction | *Nat. Commun.*, **2019**, *10*, 761 |
| The 2D cocrystal of fluoranthene and 1,2,4,5-tetracyanobenzene | about 550 | 80 | - | 74% | 0.0084, 0.0139 and 0.0147 dB/μm | *Nat. Commun.*, **2019**, *10*, 761 |
| 1,4-bis((*E*)-4-(1,2,2triphenylvinyl)styryl)-2,5dimethoxybenzene (TPDSB) | 526 | 1.70 | - | 85.6% | 0.012-0.020 dB/μm | *J. Phys. Chem. Lett.*, **2019**, *10*, 679-684 |
| Zn-IMDC (IMDC, 4,5-imidazoledicarboxylic acid) | 485 | - | 354.5 ms | 2.37% | 30–40 dB $cm^{-1}$ | *Sci. Bull.* **2022**, *67*, 2076-2084 |
| (4,4′-bis(2,5-dimethylstyryl)biphenyl, Bdb) | 460 | 1.56 | - | 82.49% | - | *Sci China Chem*, **2022**, *65*, 408–417. |
| The 1D cocrystal of 9-anthracene carboxylic acid and 1,2,4,5tetracyanobenzene (9AC-TCNB) | 583 and 652 | $\tau_1$ = 2.38 ns (40.63%) and $\tau_2$ = 6.71 ns (59.31%) | - | - | 0.006706 dB/μm at 583 nm; 0.006602 | *Adv. Opt. Mater.*, **2020**, *8*, 1901280 |

| | | | | | | |
|---|---|---|---|---|---|---|
| | | at 583 nm; τ = 5.87 ns (96.69%) at 652 nm | | | dB/μm at 652 nm | |
| The 2D cocrystal of 9-anthracene carboxylic acid and 1,2,4,5tetracyanobenzene (9AC-TCNB) | 650 | - | - | - | 0.0068306 dB/μm at 650 nm | *Adv. Opt. Mater.*, **2020**, *8*, 1901280 |
| One of polymorphic 2D cocrystals composed of 4-(4-dimethylaminostyryl)quinolone and 1,4-diiodotetrafluorobenzene (*M*DFCs) | 515 | 0.24 | - | 12% | 0.0140 dB/μm, 0.0520 dB/μm and 0.0540 dB/μm along different directions | *Angew. Chem. Int. Ed.*, **2020**, *59*, 44564463. |
| One of polymorphic 2D cocrystals composed of 4-(4-dimethylaminostyryl)quinolone and 1,4-diiodotetrafluorobenzene (*T*DFCs) | 565 | 1.91 | - | 11% | 0.104 dB/μm, 0.115 dB/μm and 0.108 dB/μm | *Angew. Chem. Int. Ed.*, **2020**, *59*, 44564463. |
| Tri-phenylene (TP)-2,3,5,6-tetrafluoro-7,7,8,8-tetracyanoquinodimethane (F4TCNQ) CT organic complex | 770 | 11.3 ns | - | 5.4% | 0.060 dB $\mu m^{-1}$ | *Adv.Mater.***2022**, *34,* 2107169. |
| Trans-1,2-Diphenylethylene and 1,2,4,5-tetracyanobenzene | 580 | 31.5 ns | $\tau_1$ = 0.69 ms and $\tau_2$ = 2.94 ms | 13.77% | - | *Angew. Chem. Int. Ed.*, **2019**, *58*, 11311 –11316. |
| Calix[3]acridan (C[3]A) and 1,2-dicyanobenzene (DCB) | 500 | 152 ns | 5.2 μs | 70% | - | *Angew. Chem. Int. Ed.*, **2022**, *61*, e202117872. |
| Through-space cocrystals of twisted aromatic hydrocarbon (TAH) donor and octafluoronaphthalene (OFN), tetrafluoropterepthanitrile (TFPN) and 1,2,4,5-tetracyanobenzene (TCNB) denoted as TAHOFN, TAHTFPN_G, TAHTFPN_O and TAHTCNB | 540<br>545<br>560<br>590 | 0.50 ns<br>0.87 ns<br>1.06 ns<br>38.81 ns | 5.38 μs | 31.6%<br>74.4%<br>77.5%<br>64.4% | OWG loss coefficient: 0.44 dB/μm, 0.0742 dB/μm 0.0837 dB/μm | ***Present work*** |

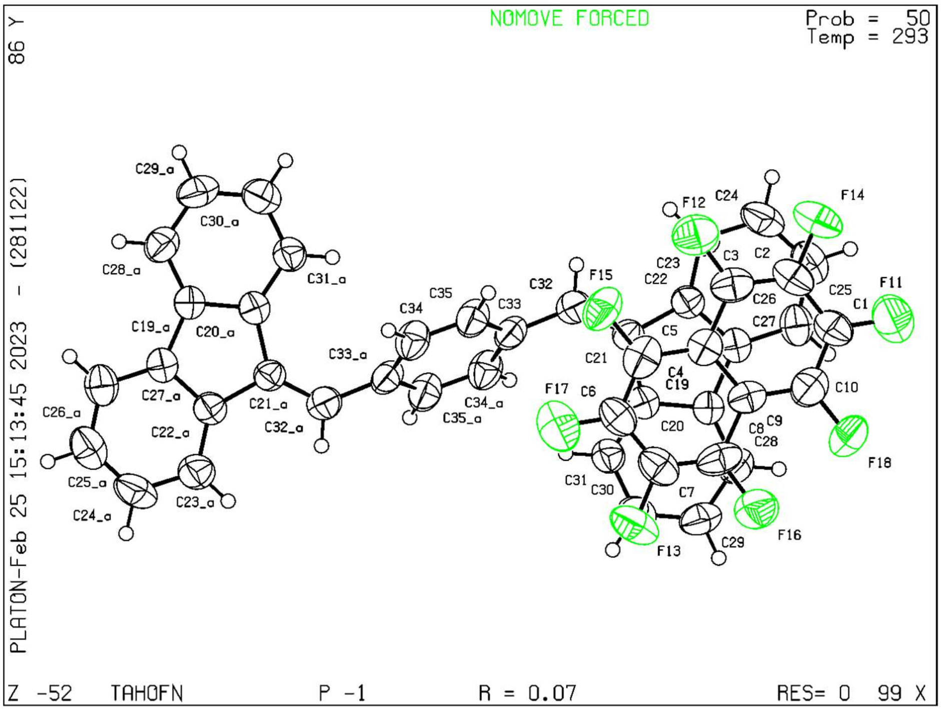


**Supplementary Fig. 59:** An ellipsoid figure of TAHOFN single crystal structure.

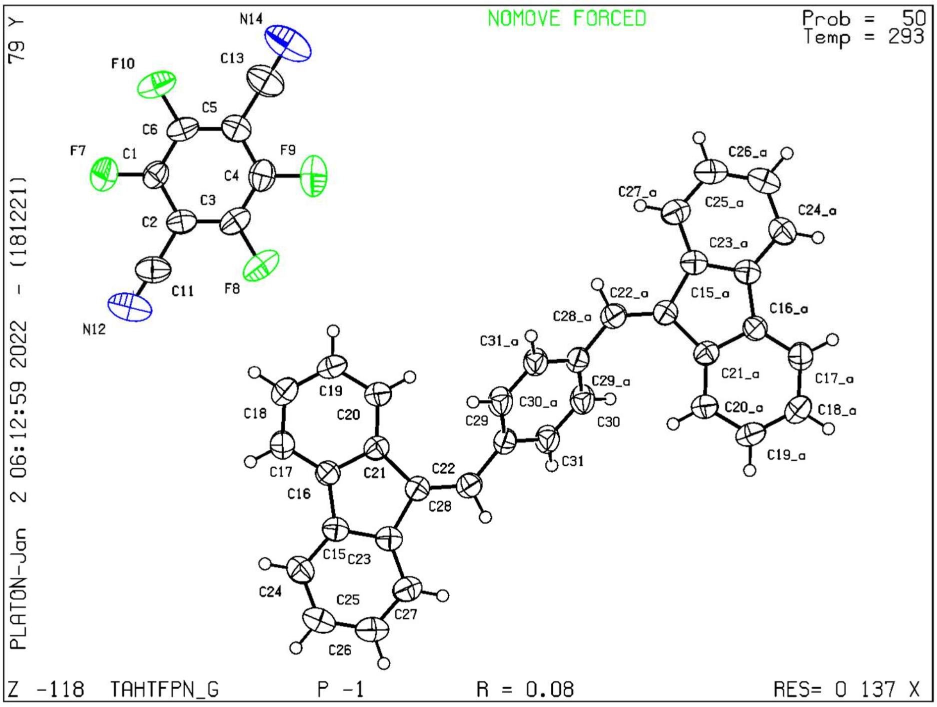


**Supplementary Fig. 60:** An ellipsoid figure of TAHTFPN_G single crystal structure.

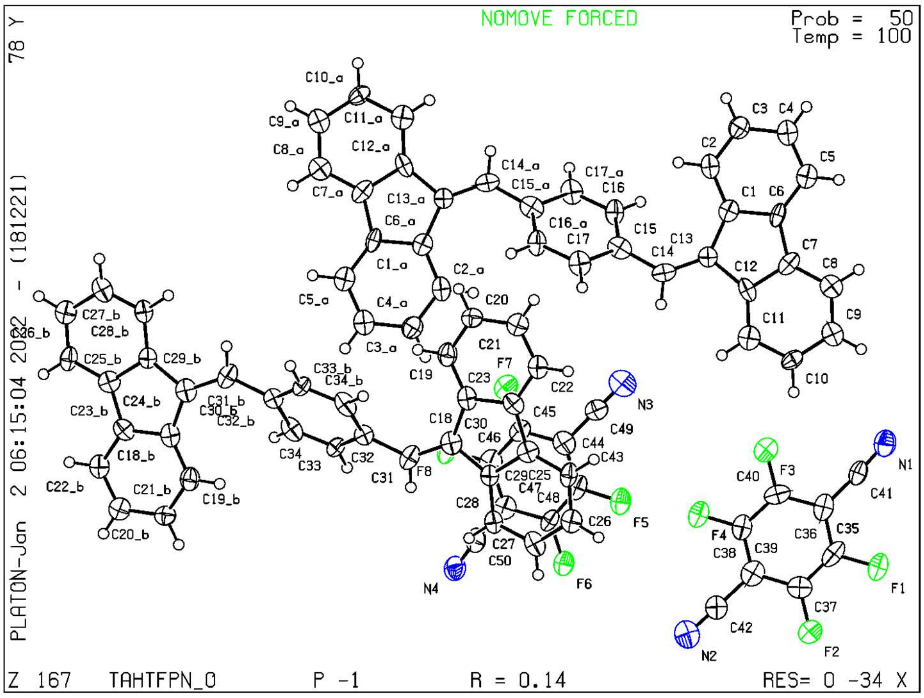


**Supplementary Fig. 61:** An ellipsoid figure of TAHTFPN_O single crystal structure.

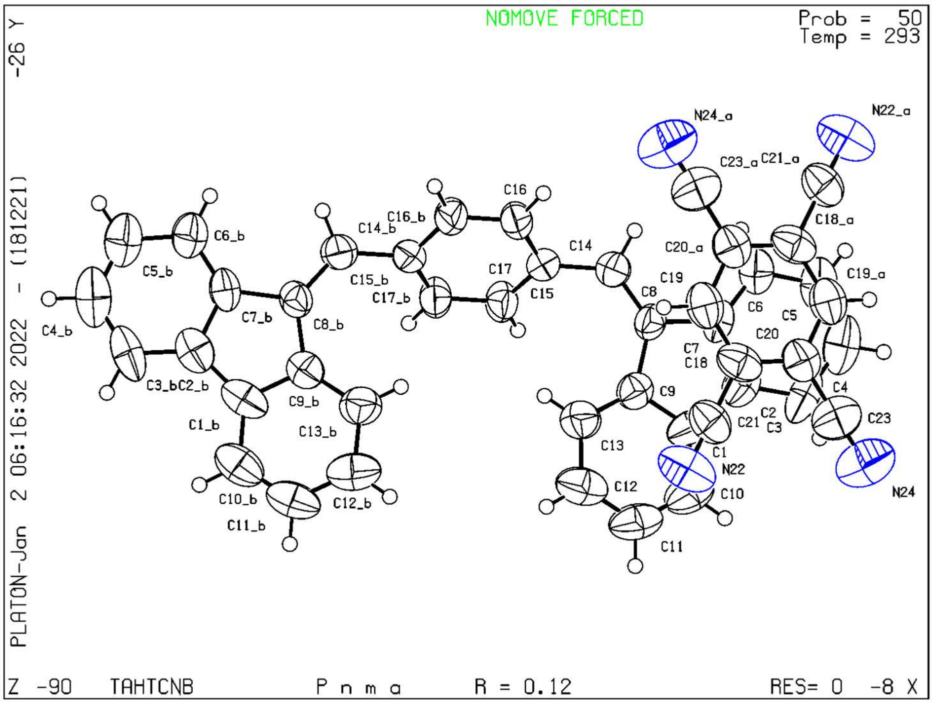


**Supplementary Fig. 62:** An ellipsoid figure of TAHTCNB single crystal structure.

**Supplementary Table S25.** Cartesian coordinates of TAHOFN used for the TD-DFT calculation at B3LYP/6-31G (d,p) level.

| | | | |
|---|---|---|---|
| F | 1.20431400 | -0.97743800 | -2.11241400 |
| F | 3.17325900 | -2.59372200 | -1.84605200 |
| F | 5.78097100 | -2.96327900 | -1.35622500 |
| F | 4.48512800 | 3.23772600 | -1.08012000 |
| F | 1.88924100 | 3.62637900 | -1.54609700 |
| F | 6.43361500 | 1.63635100 | -0.71945600 |
| F | 0.27026300 | 1.53800200 | -2.02086400 |
| F | 7.37254500 | -0.88129700 | -0.79234400 |
| C | 3.38144400 | -0.19845600 | -1.58978300 |
| C | 4.28018400 | 0.85274300 | -1.30548200 |
| C | 1.53600900 | 1.31496100 | -1.80169800 |
| C | 3.96029000 | -1.53478200 | -1.60066900 |
| C | 5.25771000 | -1.72345500 | -1.34427000 |
| C | 6.11256700 | -0.65615300 | -1.06160500 |
| C | 2.05610600 | 0.04086000 | -1.84881400 |
| C | 2.40432900 | 2.36254800 | -1.54657900 |
| C | 5.60412100 | 0.61150900 | -1.03561400 |
| C | 3.69183600 | 2.18319200 | -1.31080400 |
| C | 1.90426300 | 0.74738500 | 1.72997200 |
| C | 3.27785000 | 0.83658900 | 2.02533100 |
| C | 3.85482700 | -0.50649300 | 1.99803100 |
| C | 1.57732900 | -0.68081600 | 1.47135700 |
| C | 2.84147500 | -1.40859700 | 1.65406300 |
| C | 1.15606700 | 1.90702300 | 1.63700800 |
| H | 0.25140300 | 1.86347100 | 1.42573000 |
| C | 0.42259700 | -1.24641900 | 1.07892900 |
| H | 0.48866800 | -2.14514400 | 0.84812300 |
| C | -0.91838100 | -0.67964100 | 0.95746400 |
| C | -2.95772600 | -0.42329100 | -0.30447200 |
| H | -3.43619800 | -0.57974400 | -1.08714700 |
| C | 5.14746100 | -0.95876700 | 2.23196600 |

| | | | |
|---|---|---|---|
| H | 5.82219200 | -0.35805200 | 2.45579500 |
| C | -1.67619800 | -0.91200300 | -0.18901400 |
| H | -1.31045400 | -1.40623000 | -0.88694500 |
| C | 1.75915300 | 3.13124700 | 1.85977800 |
| H | 1.25450800 | 3.91065200 | 1.80201200 |
| C | 3.12990400 | -2.76521000 | 1.56252800 |
| H | 2.45986300 | -3.37545700 | 1.34898300 |
| C | 3.87186000 | 2.06300800 | 2.24844900 |
| H | 4.77739700 | 2.11479000 | 2.45189300 |
| C | 3.11252800 | 3.21314500 | 2.16800500 |
| H | 3.50502800 | 4.04102000 | 2.32127500 |
| C | 4.42461400 | -3.19858200 | 1.79337900 |
| H | 4.62612400 | -4.10432600 | 1.72271300 |
| C | 5.42260700 | -2.29793300 | 2.13030400 |
| H | 6.28601700 | -2.60394400 | 2.28845200 |
| C | -6.37498500 | -1.12899900 | -0.04914000 |
| C | -7.74857100 | -1.21820200 | -0.34449800 |
| C | -8.32554900 | 0.12488000 | -0.31719800 |
| C | -6.04805200 | 0.29920300 | 0.20947600 |
| C | -7.31219800 | 1.02698400 | 0.02676900 |
| C | -5.62679000 | -2.28863600 | 0.04382400 |
| H | -4.72212500 | -2.24508400 | 0.25510300 |
| C | -4.89332000 | 0.86480600 | 0.60190300 |
| H | -4.95939000 | 1.76353100 | 0.83270900 |
| C | -3.55234100 | 0.29802700 | 0.72336800 |
| C | -1.51299700 | 0.04167700 | 1.98530500 |
| H | -1.03452500 | 0.19813100 | 2.76798000 |
| C | -9.61818400 | 0.57715300 | -0.55113400 |
| H | -10.29291400 | -0.02356200 | -0.77496200 |
| C | -2.79452400 | 0.53039000 | 1.86984600 |
| H | -3.16026900 | 1.02461600 | 2.56777800 |
| C | -6.22987500 | -3.51286000 | -0.17894500 |
| H | -5.72523100 | -4.29226400 | -0.12117900 |
| C | -7.60062600 | 2.38359600 | 0.11830500 |

| | | | |
|---|---|---|---|
| H | -6.93058500 | 2.99384400 | 0.33184900 |
| C | -8.34258300 | -2.44462100 | -0.56761700 |
| H | -9.24811900 | -2.49640400 | -0.77106100 |
| C | -7.58324900 | -3.59475800 | -0.48717300 |
| H | -7.97575000 | -4.42263400 | -0.64044200 |
| C | -8.89533500 | 2.81696900 | -0.11254600 |
| H | -9.09684600 | 3.72271300 | -0.04188100 |
| C | -9.89332800 | 1.91631900 | -0.44947200 |
| H | -10.75673900 | 2.22233000 | -0.60762000 |

**Supplementary Table S26.** Cartesian coordinates of TAHTFPN_G used for the TD-DFT calculation at B3LYP/6-31G (d,p) level.

| | | | |
|---|---|---|---|
| C | -1.33669100 | -0.59839500 | 0.19057000 |
| H | -0.96262300 | -1.41801500 | -0.03945300 |
| C | 3.31147400 | 0.28780200 | 1.50708800 |
| C | 1.55918900 | 3.23059900 | 0.04910000 |
| H | 0.66665200 | 3.07125100 | -0.15859500 |
| C | 4.33144200 | 1.28847600 | 1.41853600 |
| C | 4.92157000 | -1.21922800 | 2.37414700 |
| H | 5.14898500 | -2.06525900 | 2.68591200 |
| C | -0.51360800 | 0.40473200 | 0.75170000 |
| C | 5.61499500 | 1.00592800 | 1.81215100 |
| H | 6.28183200 | 1.64912300 | 1.74198400 |
| C | -2.67780300 | -0.38547900 | -0.02278100 |
| H | -3.19352300 | -1.05715300 | -0.40587200 |
| C | 3.69963200 | 2.49527600 | 0.87864300 |
| C | 3.62821900 | -0.97952100 | 1.99126700 |
| H | 2.97987900 | -1.64470700 | 2.05340500 |
| C | 5.89788900 | -0.24343400 | 2.31100500 |
| H | 6.75762200 | -0.43582700 | 2.61036200 |
| C | 2.32390400 | 2.23396300 | 0.63190900 |
| C | 0.90727000 | 0.09091200 | 1.02418400 |
| H | 1.05623200 | -0.80353000 | 1.23014100 |

| | | | |
|---|---|---|---|
| C | 2.05380600 | 0.85317900 | 1.03522200 |
| C | 4.25775500 | 3.73486100 | 0.59938100 |
| H | 5.15429100 | 3.89654000 | 0.78111300 |
| C | 2.12239200 | 4.46848500 | -0.22634600 |
| H | 1.60116500 | 5.14135000 | -0.59934600 |
| C | 3.48113200 | 4.71210000 | 0.05671000 |
| H | 3.84840800 | 5.54620100 | -0.12864700 |
| C | -2.46173600 | 1.84533800 | 0.89220300 |
| H | -2.83580500 | 2.66495800 | 1.12222600 |
| C | -7.10990100 | 0.95914100 | -0.42431400 |
| C | -5.35761700 | -1.98365600 | 1.03367400 |
| H | -4.46508000 | -1.82430800 | 1.24136900 |
| C | -8.12987000 | -0.04153300 | -0.33576200 |
| C | -8.71999700 | 2.46617100 | -1.29137300 |
| H | -8.94741300 | 3.31220200 | -1.60313800 |
| C | -3.28482000 | 0.84221100 | 0.33107400 |
| C | -9.41342200 | 0.24101500 | -0.72937700 |
| H | -10.08026000 | -0.40218000 | -0.65921100 |
| C | -1.12062500 | 1.63242200 | 1.10555500 |
| H | -0.60490500 | 2.30409600 | 1.48864500 |
| C | -7.49806000 | -1.24833300 | 0.20413100 |
| C | -7.42664700 | 2.22646400 | -0.90849300 |
| H | -6.77830600 | 2.89165000 | -0.97063100 |
| C | -9.69631600 | 1.49037700 | -1.22823100 |
| H | -10.55605000 | 1.68277000 | -1.52758900 |
| C | -6.12233200 | -0.98702000 | 0.45086400 |
| C | -4.70569800 | 1.15603200 | 0.05859000 |
| H | -4.85466000 | 2.05047300 | -0.14736800 |
| C | -5.85223400 | 0.39376400 | 0.04755200 |
| C | -8.05618300 | -2.48791800 | 0.48339300 |
| H | -8.95271800 | -2.64959700 | 0.30166100 |
| C | -5.92082000 | -3.22154200 | 1.30911900 |
| H | -5.39959300 | -3.89440700 | 1.68212000 |
| C | -7.27955900 | -3.46515700 | 1.02606400 |

| | | | |
|---|---|---|---|
| H | -7.64683600 | -4.29925800 | 1.21142000 |
| F | 5.59008400 | -3.61330100 | -0.21460900 |
| F | 6.98431200 | -1.35209600 | -0.57411100 |
| F | 1.70121000 | -1.49673700 | -1.84616200 |
| F | 3.14664300 | 0.76780700 | -2.24909500 |
| N | 2.41624700 | -4.85772200 | -0.78239300 |
| N | 6.52429900 | 1.86404500 | -1.88372800 |
| C | 2.91639300 | -3.82657900 | -0.90088400 |
| C | 3.02399400 | -1.47020400 | -1.54644500 |
| C | 3.63633300 | -2.60039600 | -1.04201500 |
| C | 5.88283500 | 0.93099000 | -1.66341500 |
| C | 4.99513000 | -2.54639800 | -0.71464200 |
| C | 5.08548500 | -0.25513300 | -1.43069300 |
| C | 3.74391800 | -0.31907600 | -1.74878800 |
| C | 5.68896000 | -1.36318400 | -0.90891000 |

**Supplementary Table S27.** Cartesian coordinates of TAHTFPN_O used for the TD-DFT calculation at B3LYP/6-31G (d,p) level.

| | | | |
|---|---|---|---|
| F | 9.33991505 | -2.17941218 | -0.01933637 |
| F | 8.49587813 | -4.71810680 | 0.13040608 |
| F | 4.89415717 | -0.72459117 | -0.39469103 |
| F | 4.05254108 | -3.26459618 | -0.28671592 |
| C | 7.13181471 | -1.39957737 | -0.19126929 |
| C | 8.03328673 | -2.44104707 | -0.05364994 |
| C | 7.59863784 | -3.73678116 | 0.00823376 |
| C | 5.78828802 | -1.70376792 | -0.25854922 |
| C | 6.25385494 | -4.04447945 | -0.05510540 |
| N | 7.97728734 | 1.02946227 | -0.34273084 |
| C | 7.59012189 | -0.04052310 | -0.27462742 |
| C | 5.35139584 | -3.00067634 | -0.20227769 |
| C | 5.77742740 | -5.39697473 | 0.01990378 |
| N | 5.40954295 | -6.46405451 | 0.10723481 |
| C | 0.19510833 | 5.48696190 | 0.02212601 |

| | | | |
|---|---|---|---|
| C | -0.25386017 | 4.07098387 | 0.06025007 |
| C | 1.58838067 | 5.53739106 | 0.13987120 |
| C | 0.99605072 | 3.27179863 | 0.20017973 |
| C | 2.08680070 | 4.16440908 | 0.25053731 |
| C | -1.54088615 | 3.71044816 | -0.07896366 |
| H | -2.11716597 | 4.42027408 | -0.25153237 |
| C | -2.20660998 | 2.40592888 | -0.00867103 |
| C | 1.24432686 | 1.90375125 | 0.19347758 |
| H | 0.54211432 | 1.29845713 | 0.11894741 |
| C | -3.25105781 | 2.11781889 | -0.88349820 |
| H | -3.49889675 | 2.74975253 | -1.51878047 |
| C | -0.53759245 | 6.65999355 | -0.11293926 |
| H | -1.46392939 | 6.63307367 | -0.18559525 |
| C | 2.25654661 | 6.75456372 | 0.12342767 |
| H | 3.18218938 | 6.78779928 | 0.20780573 |
| C | 0.13502767 | 7.86738267 | -0.13888995 |
| H | -0.34576414 | 8.65728127 | -0.23685487 |
| C | -3.92250976 | 0.92459123 | -0.82995729 |
| H | -4.60559023 | 0.75810737 | -1.43770527 |
| C | 3.38381273 | 3.69805290 | 0.35151125 |
| H | 4.09574362 | 4.29480974 | 0.39661010 |
| C | 3.61087211 | 2.33804497 | 0.38438451 |
| H | 4.47882911 | 2.01481062 | 0.46493660 |
| C | 1.51634481 | 7.91997734 | -0.02035519 |
| H | 1.95054552 | 8.74224570 | -0.03812612 |
| C | 2.55202927 | 1.45347821 | 0.29916364 |
| H | 2.71956709 | 0.53901454 | 0.31329860 |
| C | -5.99952595 | -3.12762111 | 0.09137480 |
| C | -5.55055757 | -1.71164404 | 0.05325061 |
| C | -7.39279837 | -3.17805010 | -0.02637138 |
| C | -6.80046847 | -0.91245881 | -0.08667905 |
| C | -7.89121856 | -1.80506912 | -0.13703761 |
| C | -4.26353156 | -1.35110722 | 0.19246349 |
| H | -3.68725188 | -2.06093412 | 0.36503208 |

| | | | |
|---|---|---|---|
| C | -3.59780775 | -0.04658906 | 0.12217172 |
| C | -7.04874446 | 0.45558956 | -0.07997677 |
| H | -6.34653192 | 1.06088368 | -0.00544660 |
| C | -2.55335992 | 0.24152094 | 0.99699889 |
| H | -2.30552098 | -0.39041271 | 1.63228116 |
| C | -5.26682515 | -4.30065276 | 0.22644007 |
| H | -4.34048821 | -4.27373284 | 0.29909606 |
| C | -8.06096423 | -4.39522293 | -0.00992686 |
| H | -8.98660711 | -4.42845834 | -0.09430591 |
| C | -5.93944528 | -5.50804189 | 0.25239076 |
| H | -5.45865356 | -6.29794032 | 0.35035470 |
| C | -1.88190784 | 1.43474958 | 0.94345810 |
| H | -1.19882749 | 1.60123359 | 1.55120509 |
| C | -9.18823047 | -1.33871308 | -0.23801057 |
| H | -9.90016135 | -1.93546879 | -0.28311028 |
| C | -9.41528984 | 0.02129485 | -0.27088382 |
| H | -10.28324684 | 0.34452920 | -0.35143591 |
| C | -7.32076253 | -5.56063750 | 0.13385588 |
| H | -7.75496325 | -6.38290476 | 0.15162595 |
| C | -8.35644696 | 0.90586274 | -0.18566381 |
| H | -8.52398469 | 1.82032626 | -0.19979779 |

**Supplementary Table S28.** Cartesian coordinates of TAHTCNB used for the TD-DFT calculation at B3LYP/6-31G (d,p) level.

| | | | |
|---|---|---|---|
| C | 1.63148405 | 1.21532597 | 0.85740622 |
| C | 1.95876617 | -0.21160838 | 1.20535204 |
| C | 3.36749467 | -0.18823736 | 1.70545831 |
| C | 3.82059749 | 1.15423151 | 1.70411015 |
| C | -0.25017040 | -1.44115189 | 0.89192368 |
| C | 1.19878805 | -1.31775405 | 1.13176087 |
| C | -1.15276279 | -0.65435814 | 1.56306004 |
| C | 0.50575998 | 1.78261518 | 0.25299394 |
| C | 2.75880993 | 1.99678519 | 1.15424080 |

| | | | |
|---|---|---|---|
| C | 4.15822365 | -1.20230540 | 2.17310712 |
| C | -0.76886772 | -2.40566972 | 0.03041494 |
| C | 5.10704425 | 1.42298850 | 2.18774410 |
| C | 5.43895383 | -0.90060013 | 2.62961701 |
| C | 0.52274503 | 3.15627162 | -0.01220688 |
| C | 1.62849410 | 3.92454302 | 0.34505578 |
| C | 2.73325092 | 3.37223235 | 0.91263167 |
| C | 5.90059711 | 0.36812841 | 2.62681298 |
| H | 3.46281197 | 3.90344160 | 1.13724159 |
| H | 1.61032072 | 4.84149229 | 0.19085411 |
| H | -0.20598305 | 3.55743980 | -0.42803910 |
| H | -0.23297258 | 1.26201708 | 0.03354117 |
| H | 3.84575774 | -2.07813545 | 2.18591544 |
| H | 5.98572204 | -1.58504027 | 2.94184252 |
| H | 6.76493527 | 0.54002340 | 2.92387090 |
| H | 5.42691647 | 2.29582678 | 2.21505136 |
| H | 1.65260855 | -2.12098250 | 1.24910901 |
| H | -0.84166536 | -0.03151078 | 2.17965088 |
| H | -0.18812000 | -2.98618756 | -0.40620096 |
| C | -5.20909998 | 0.65499827 | -0.24284264 |
| C | -5.39773505 | -0.81419603 | 0.02212236 |
| C | -6.87707582 | -1.02739328 | 0.05770764 |
| C | -7.51415660 | 0.22577619 | -0.11898714 |
| C | -3.03547231 | -1.66930226 | 0.44393193 |
| C | -4.48729177 | -1.78351370 | 0.21720404 |
| C | -2.52374686 | -0.76665855 | 1.34254910 |
| C | -4.05612192 | 1.40894112 | -0.48074536 |
| C | -6.48174464 | 1.23987048 | -0.33202258 |
| C | -7.60270779 | -2.16566987 | 0.28146289 |
| C | -2.12809183 | -2.51700684 | -0.18820451 |
| C | -8.91273216 | 0.27459693 | -0.06721584 |
| C | -8.99270492 | -2.08272992 | 0.30840936 |
| C | -4.20735460 | 2.76881846 | -0.77300255 |
| C | -5.47651700 | 3.34255550 | -0.79772389 |

| | | | |
|---|---|---|---|
| C | -6.60075158 | 2.60766311 | -0.58866201 |
| C | -9.62563430 | -0.90366029 | 0.12955277 |
| H | -7.43852775 | 3.01048823 | -0.61614474 |
| H | -5.55693062 | 4.25440653 | -0.96193637 |
| H | -3.45871371 | 3.29100128 | -0.95121273 |
| H | -3.21304319 | 1.01791276 | -0.44577744 |
| H | -7.17499435 | -2.98087016 | 0.41332266 |
| H | -9.49364861 | -2.85299050 | 0.45211946 |
| H | -10.55524543 | -0.87871174 | 0.13806944 |
| H | -9.35940719 | 1.08464560 | -0.16320108 |
| H | -4.82159586 | -2.65129921 | 0.20778915 |
| H | -3.10611972 | -0.21699735 | 1.81543296 |
| H | -2.44406978 | -3.17097750 | -0.76905100 |
| C | 5.17760568 | 0.52991943 | -1.33330488 |
| C | 3.90737749 | 0.53616979 | -1.89971368 |
| N | 2.80989919 | 2.71575885 | -2.70620973 |
| C | 3.29591895 | 1.75404110 | -2.34564912 |
| C | 3.20805823 | -0.66848800 | -2.08640062 |
| C | 5.89494443 | 1.74801136 | -1.12814402 |
| N | 6.48103300 | 2.70149154 | -0.96080579 |
| H | 2.35998440 | -0.65495722 | -2.46779975 |
| C | 3.77262430 | -1.87863384 | -1.70625835 |
| C | 5.04285250 | -1.88488420 | -1.13984955 |
| N | 6.14033080 | -4.06447326 | -0.33335350 |
| C | 5.65431103 | -3.10275551 | -0.69391411 |
| C | 5.74217176 | -0.68022641 | -0.95316261 |
| C | 3.05528553 | -3.09672577 | -1.91141920 |
| N | 2.46919697 | -4.05020595 | -2.07875744 |
| H | 6.59024561 | -0.69375719 | -0.57176348 |

**Supplementary References**


(1) Material Studio 8.0, Acceryls Inc., San Diego, 2014.

(2) Spackman, M. A. & Jayatilaka, D. Hirshfeld surface analysis *CrystEngCom.,* ***11***, 19−32 (2009).

(3) Spackman, M. A.; McKinnon, J. J.; Jayatilaka, D. *CrystEngComm*. ***10***, 377−388 (2008).

(4) Frisch, M. J. et al. Gaussian, Inc., Wallingford CT, 2016.

(5) Salla, C. A. M. et al. *Angew. Chem., Int. Ed., 58*, 6982– 6986, (2019).
