# Supplementary material for: Highly efficient color-tunable organic co-crystals unveiling polymorphism, isomerism, delayed fluorescence for optical waveguides and cell-imaging
Source: Nat Commun. 2023 Oct 20;14:6648. doi: 10.1038/s41467-023-42017-8 (PMC10589249; doi:10.1038/s41467-023-42017-8)
Supplement: Supplementary file 6 — Reporting Summary [file 41467_2023_42017_MOESM6_ESM.pdf]

Corresponding author(s): Siddartha Sankar Ghosh, Rajadurai Chandrasekar & Parameswar Krishnan Iyer

Last updated by author(s): Sep 10, 2023

Reporting Summary

Nature Portfolio wishes to improve the reproducibility of the work that we publish. This form provides structure for consistency and transparency in reporting. For further information on Nature Portfolio policies, see our Editorial Policies and the Editorial Policy Checklist.

Statistics

For all statistical analyses, confirm that the following items are present in the figure legend, table legend, main text, or Methods section.

n/a	Confirmed	
☐	☒	The exact sample size (*n*) for each experimental group/condition, given as a discrete number and unit of measurement
☐	☒	A statement on whether measurements were taken from distinct samples or whether the same sample was measured repeatedly
☐	☒	The statistical test(s) used AND whether they are one- or two-sided<br>*Only common tests should be described solely by name; describe more complex techniques in the Methods section.*
☒	☐	A description of all covariates tested
☐	☒	A description of any assumptions or corrections, such as tests of normality and adjustment for multiple comparisons
☐	☒	A full description of the statistical parameters including central tendency (e.g. means) or other basic estimates (e.g. regression coefficient) AND variation (e.g. standard deviation) or associated estimates of uncertainty (e.g. confidence intervals)
☒	☐	For null hypothesis testing, the test statistic (e.g. *F*, *t*, *r*) with confidence intervals, effect sizes, degrees of freedom and *P* value noted<br>*Give P values as exact values whenever suitable.*
☒	☐	For Bayesian analysis, information on the choice of priors and Markov chain Monte Carlo settings
☒	☐	For hierarchical and complex designs, identification of the appropriate level for tests and full reporting of outcomes
☒	☐	Estimates of effect sizes (e.g. Cohen's *d*, Pearson's *r*), indicating how they were calculated

Our web collection on statistics for biologists contains articles on many of the points above.

Software and code

Policy information about availability of computer code

Data collection	Data were collected with various instruments and equipments such as: Bruker Advance 400 spectrometer (NMR), Perkin Elmer instrument in attenuated total reflectance (ATR) mode, Horiba Jobin Vyon,LabRam HR, Rigaku Smartlab X-ray diffractometer equipped with Oxford cryo-system (80 500K) and crystalAlisPRO software and Autochem softwares, PerkinElmer, ModelLambda-25 spectrometer and Horiba·Fluoromax4 with Varian Cary Eclipse spectrometer,Horiba Fluoromax (JobinYvon equipped with integrating sphere).Edinburgh Life Spec II instrument,liquid nitrogen·cooled opticalcryostat (Optistat,Oxford Instruments) attach to an Edinburgh FSP·920 instrument, LP980.KS spectrometer with a visible PMT detector,.JES-FA200 ESR spearometer, Zeiss, Model:Sigma-300, Asylum Cypher, Oxford Instruments JEOL JEM·2100F miroscopes, CH Instruments 7600 electrochemical workstation, Mettler·Toledo TGA/SDTA 851e thermogravimetric analyzer, Wi-Tec alpha 200 laser confocal optical microscope facility equipped with a Peltier-cooled CCD detector, ZEISS Axio Vert.A1 inverted microscope with 10X objective, multiplate reader (Infinite 200 PRO, TECAN).
Data analysis	Software used: ChemDraw Ultra 19.0, Adobe Illustrator, Origin 8.5 pro, MATLAB R2017a, Gaussiun 16 package, GaussView 05, ORCA 4.2.0. Multiwfn, Mercury, Materials Studio software, Crystal Explorer, 3.1.51, Compusyn software was used for combination index analysis, cell imaging images were processed using WITEC 2.0 software and all the figures were compiled in Microsoft PowerPoint 2016.

For manuscripts utilizing custom algorithms or software that are central to the research but not yet described in published literature, software must be made available to editors and reviewers. We strongly encourage code deposition in a community repository (e.g. GitHub). See the Nature Portfolio guidelines for submitting code & software for further information.

Data

Policy information about availability of data

All manuscripts must include a data availability statement. This statement should provide the following information, where applicable:

- Accession codes, unique identifiers, or web links for publicly available datasets
- A description of any restrictions on data availability
- For clinical datasets or third party data, please ensure that the statement adheres to our policy

Supplementary information file contains all additional experimental data, tables and results. The data that support the findings of this study are available from the authors on request. The X-ray crystallographic coordinates for structure have been deposited with the Cambridge Crystallographic Data Center (CCDC) (accession codes CCDC 2109333, CCDC 2109336, CCDC 2109335, and CCDC 2109337). These data can be obtained free of charge from The Cambridge Crystallographic Data Center via www.ccdc.cam.ac.uk/data_request/cif. The source data underlying main manuscript Figs. 3-9 and Supplementary Figs. 2–58 are provided as a Source Data file.

Human research participants

Policy information about studies involving human research participants and Sex and Gender in Research.

Reporting on sex and gender	NA
Population characteristics	NA
Recruitment	NA
Ethics oversight	NA

Note that full information on the approval of the study protocol must also be provided in the manuscript.

Field-specific reporting

Please select the one below that is the best fit for your research. If you are not sure, read the appropriate sections before making your selection.

☒ Life sciences ☐ Behavioural & social sciences ☐ Ecological, evolutionary & environmental sciences

For a reference copy of the document with all sections, see nature.com/documents/nr-reporting-summary-flat.pdf

Life sciences study design

All studies must disclose on these points even when the disclosure is negative.

Sample size	Triplicate from biologically independent samples were used to evaluate the repeatability of our experiments. Animal models were not included in the study.
Data exclusions	No data exclusions were performed.
Replication	Experiments were replicated three times (biological replicates), ensuring reproducibility. Replication attempts were successful,and precise numerical data were provided in the figure legends.
Randomization	In all experiments, samples were randomly assigned to different experimental groups.
Blinding	Blinding was not necessary for the studies included in this manuscript. Since, Animal models were not included in the study.

Reporting for specific materials, systems and methods

We require information from authors about some types of materials, experimental systems and methods used in many studies. Here, indicate whether each material, system or method listed is relevant to your study. If you are not sure if a list item applies to your research, read the appropriate section before selecting a response.

Materials & experimental systems

n/a	Involved in the study
☒	☐ Antibodies
☐	☒ Eukaryotic cell lines
☒	☐ Palaeontology and archaeology
☒	☐ Animals and other organisms
☒	☐ Clinical data
☒	☐ Dual use research of concern

Methods

n/a	Involved in the study
☒	☐ ChIP-seq
☒	☐ Flow cytometry
☒	☐ MRI-based neuroimaging

Eukaryotic cell lines

Policy information about cell lines and Sex and Gender in Research

Cell line source(s)	MCF-7 breast cancer cells (Cat.no.8R-MCF7-MEM-NEAA) were procured from the National Centre for Cell Science in Pune, India.
Authentication	Cells were authenticated with STR phenotyping by supplier. The cells were cultured in a C02 incubator using DMEM (Ou becco's Modified Eagle Medium) supplemented with 10% fetal bovine serum and a 1% penicillin and streptomycin antibiotic solution. The authenticity of the MCF-7 cells was confirmed through STR phenotyping and a test conducted on 04/07/2023 verified the absence of mycoplasma contamination.
Mycoplasma contamination	Cells were negative for mycoplasma contamination.
Commonly misidentified lines (See ICLAC register)	There are no commonly misidentified cell lines in the study.
